# Social experience and social cohabitation with mating promote spinogenesis in the nucleus accumbens of adult female prairie voles (*Microtus ochrogaster*)

**DOI:** 10.1371/journal.pone.0335626

**Published:** 2025-11-03

**Authors:** Analía E. Castro, Marco A. López-Quiroz, Daniela Ávila-González, Francisco J. Camacho, Raúl G. Paredes, Néstor F. Díaz, Wendy Portillo

**Affiliations:** 1 Instituto de Neurobiología, Universidad Nacional Autónoma de México (UNAM), Querétaro, México; 2 Escuela Nacional de Estudios Superiores, Unidad Juriquilla, UNAM, Querétaro, México; 3 Instituto de Ciencias de la Atmósfera y Cambio Climático, UNAM, México, México; 4 Instituto Nacional de Perinatología, México, México; University of Texas at Austin, UNITED STATES OF AMERICA

## Abstract

Prairie voles (*Microtus ochrogaster)* are monogamous rodents that establish life-long pair-bonds and display characteristic social and biparental care behaviors. Since social and sexual experiences modulate brain plasticity, the present study aimed to elucidate in female voles if social exposure to a male or social cohabitation with mating, which leads to pair-bonding, modulates spinogenesis processes in the medium spiny neurons (MSNs) in the nucleus accumbens (NAc). Females were randomly assigned to one of the following groups: 1) control (C), voles that cohabited with a familiar female in a clean cage; 2) social exposure (SE), voles housed in a cage divided into two equal compartments by an acrylic screen with small holes. The experimental female was placed in one of the compartments, and a male in the opposite one. Therefore, females were exposed to sensory cues from an adult male. Still, physical contact and copulation were not allowed, and 3) social cohabitation with mating (SCM) females were allowed to mate to induce pair-bonds. The NAc core and shell were processed for Golgi-Cox staining. Our results showed that MSN from SE and SCM groups had higher spine density than C females and a differential density of spine subtypes in the core and shell. Furthermore, only the SE condition induced an increment in MSN dendritic length and arborization in the core and shell regions. These findings demonstrate that males’ sexual cues and mating that promote pair-bonding modulate spinogenesis in the NAc and contribute to understanding the neuronal plasticity mechanism involved in pair-bonding in prairie voles.

## Introduction

Prairie voles (*Microtus ochrogaster*) are socially monogamous rodents that form stable pair-bonds after 24 h of cohabitation without mating or after 6 h when mating occurs, these bonds are subsequently maintained long term [1–3]. Pair-bonding is manifested by partner preference, territorial and nest defense, mate guarding, and biparental care of offspring [4,5]. These traits make prairie voles an excellent model for investigating the neural basis of social monogamy.

Social experience and mating that lead to pair-bonding formation induce neuronal plasticity in adult voles. Documented mechanisms include alterations in gene expression, synaptic activity, circuit dynamics, adult neurogenesis, and epigenetic modifications [6–10].

The mesolimbic reward system, specifically the nucleus accumbens (NAc), processes socio-sexual stimuli in mammals. Located in the ventral forebrain, the NAc regulates reward, motivation, reproductive behavior, drug addiction, food intake, stress-related behavior, among others [11–15]. Anatomically and functionally, the NAc comprises core and shell subregions. The core is associated with motivation, conditioned responses, and associative learning; whereas the shell processes primary rewards, aversive stimuli, and unconditioned aspects of motivated behavior [16–22]. Distinct medium spiny neuron (MSN) subpopulations within these subregions confer unique intrinsic properties.

Multiple lines of evidence implicate the NAc in pair-bond formation and maintenance [23–26]. Chemogenetic inhibition of the NAc via Designer Receptors Exclusively Activated by Designer Drugs (DREADDs) diminishes partner preference [27]. Dopamine in the NAc regulates pair-bond dynamics through differential expression and activity of D1- and D2-like receptors in male voles [24]. Partner seeking, anticipation of partner access and interaction, elevates NAc dopamine release, an effect not elicited by exposure to novel conspecific or food; prolonged partner separation (four weeks) decreases dopamine release [28]. In female voles, up-regulation of oxytocin receptors facilitates partner preference [29]; whereas NAc specific knock-down of the receptor disrupts partner preference formation [30]. Thus, dopaminergic and oxytocinergic signaling converge in the NAc to consolidate social monogamy in voles [31]. Resting-state functional magnetic resonance imaging further reveals functional connectivity between the NAc and other brain regions during affiliative behavior; including pair-bonding and huddling [7].

MSN constitutes the predominant neural population in both NAc core and shell and modulates reward and aversion [32]. Although dopaminergic inputs dominate, intrinsic GABAergic networks and cholinergic interneurons also shape MSN electrophysiology in rats [33,34].

Neuronal plasticity in the NAc is reflected morphologically by alterations in MSN dendritic arborization and spine density. Such plasticity underlines learning, memory [35], and addiction to opioids, cocaine, and alcohol [36,37]. For instance, in rats, cocaine administration increases dendritic spine density on D1-expressing MSN [38–40], whereas prolonged opioid self-administration reduces dendritic branching and spiny density [41]. In rats, prenatal alcohol exposure decreases dendritic length and branching, though not spine density [42].

Dendritic spines -small protrusions that form the postsynaptic component of excitatory synapses- comprise a neck and a bulbous head [43–45]. Based on head morphology and length, spines are classified as filopodia, long-thin, thin, stubby, mushroom, or branched; larger heads correlate with greater synaptic strength [44,46–49]. We investigated whether social and socio-sexual interactions that induce pair-bonding modulate spinogenesis in the NAc of adult female voles. We hypothesize that these interactions would increase dendritic spine density and favors mature morphologies on NAc MSN.

## Materials and methods

### Animals

Adult prairie voles were bred in our local colony at Instituto de Neurobiología, UNAM, from founders provided by Dr. Larry J. Young (Emory University). Animals were maintained on a 14 h/10 h light/dark cycle (lights on 08:00 h) with *ad libitum* access to food (rabbit high-fiber diet 5326 LABDIET, oats, and sunflower seeds) and water. BVSc Francisco J Camacho maintained the animal colony, who also trains our staff involved in animal research. All procedures conformed to the “Reglamento de la Ley General de Salud en Materia de Investigación para la Salud” of the Mexican Health Ministry, and NIH guidelines. The protocol was approved by the Institutional Ethics Animal Care Committee of the Instituto de Neurobiología (protocol 072) and the Committee of Ethics Animal care of the Instituto Nacional de Perinatología (protocol 2022-1-13). All surgery was performed under a solution of ketamine (60 mg/Kg) and xylazine (4 mg/Kg) diluted in 0.9% NaCl; all efforts were made to minimize suffering. Animal health and behavior were monitored daily.

### Experimental design

**Fig 1:**
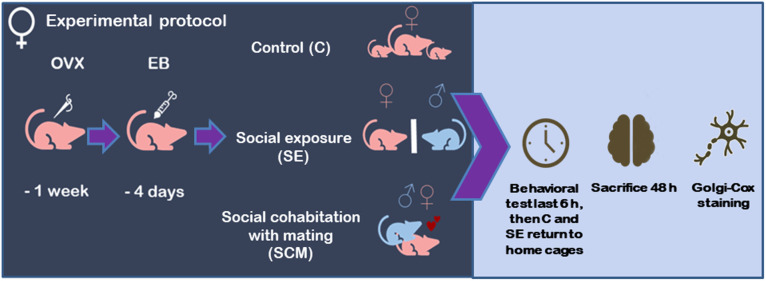
Experimental design. Sexually naive female prairie voles were bilaterally ovariectomized (OVX). After a 7-day recovery, animals received subcutaneous estradiol-benzoate injections (EB; 0.5 μg day) for four consecutive days to induce sexual receptivity. Females were then randomly assigned to one of three conditions: 1) Control (C) housed with a conspecific females; 2) Social exposure (SE), a female and an unfamiliar male were placed in opposite compartments of a divided cage, separated by a perforated acrylic barrier, permitting visual, acoustic and olfactory contact but not physical interaction; 3) Social cohabitation with mating (SCM), a female cohabited freely with a male, allowing unrestricted interaction and copulation. The behavioral session lasted six hours. Females from the SE were then returned to their original cages, whereas C and SCM pairs remained together until euthanasia. All subjects were sacrificed 48 h after the onset of the behavioral test.

Sexually naïve females (3–4 months) and stimulus males were used. Males were gonadally intact, females were bilaterally ovariectomized on Day 0, monitored and cleaned with antiseptic solution Microdacyn (Na < 55 ppm, Cl < 80 ppm. SanFer, México) and allowed to recovery for 7 days (Days 1–7). To induce sexual receptivity, females received estradiol benzoate (EB, 0.5 µg/vole, s.c., Sigma Aldrich) once daily for four consecutive days (Days 8–11) [50–52]. On Day 11, immediately after the fourth EB injection, the 6 h behavioral session began (t = 0). Females were randomly assigned to three experimental conditions (Fig 1). 1) Control (C, n = 7), housed with a familiar female in a standard clean acrylic cage (20 x 46 x 25 cm). 2) Social exposure (SE, n = 6), each female and an unfamiliar male were placed in a standard acrylic cage divided into two equal compartments by a perforated acrylic barrier that permitted visual, auditory, and olfactory, but not physical, contact. Across repeated implementations of this test in our laboratory, animals typically exhibited sustained social investigation at the divider (sniffing), with some biting or attempts to breach the barrier. While these qualitative observations suggest that hyperlocomotion is not the dominant state. 3) Social cohabitation with mating (SCM, n = 6), a female cohabited freely with an unfamiliar male, allowing interaction and mating (a condition known to accelerate pair-bonding).

Twelve males served as stimulus animals. After the 6 h session, SE and C females were returned to their home cages, SE females were returned to their sibling cages, because pair-bonds form after 24 h of non-mating cohabitation [2]. In contrast, SCM pairs remained together until euthanasia. All females were euthanized 48 h after the start of the behavioral session (Day 13) by decapitation. This was necessary to obtain the brains to process for Golgi-Cox staining. No deaths, symptoms of disease, or discomfort occurred during the 13-day experimental timeline. Stimulus males were returned to the colony for use in other experiments.

#### Pairing conditions and verification of mating.

At t = 0, each EB-primed, ovariectomized SCM female was first introduced to an unfamiliar, non-sibling male; pairs were drawn from different home cages and had no prior cohabitation or physical interactions. Male sexual behavior during the first hour was scored by a trained observer using a predefined ethogram (mount, intromission, ejaculation). For each pair, we recorded latencies to first mount, intromission, and ejaculation. And the number of mounts and intromissions. As summarized in Table 1, all males (n = 6) ejaculated at least once within the first hour, confirming consummatory interaction at session onset.

**Table 1:** Sexual behavior parameters displayed by the social cohabitation with mating group and registered during the first 1 h of the behavioral test *(n = *6)*.*

Sexual parameter	Range	Max	Min	Median	25%	75%
**MN**	**11**	**16**	**5**	**8.5**	**5.75**	**14.5**
**IN**	**2**	**7**	**5**	**5.5**	**5**	**6.25**
**EN**	**0**	**1**	**1**	**1**	**1**	**1**
**ML**	**513.6**	**694.2**	**180.6**	**264**	**184.7**	**410.3**
**IL**	**2132**	**2642**	**510**	**1451**	**552.8**	**2472**
**EL**	**637.8**	**2472**	**1834**	**2130**	**1834**	**2472**

Number of mounts (MN), intromissions (IN), and ejaculations (EN). Latency of mount (LM), intromission (LI) and ejaculation (LE).

### Tissue processing and Golgi-Cox staining

Prairie voles were euthanized by decapitation. Heads were chilled on ice for 15 min to minimize tissue degradation, brains were extracted, rinsed in ice-cold PBS (10 min), and incubated in PBS containing heparin (1000 IU ml^-1^, 1:1000; 10 min) until incubation with the staining Golgi-Cox kit solutions.

Brains were sectioned into four blocks using an adult mouse brain matrix and processed with the FD Rapid GolgiStain^TM^ kit (#PK 401, FD NeuroTechnologies, INC) following the manufacturer’s protocol with minor modifications. Blocks were immersed in impregnation solution (equal parts solution A and B; prepared 24 h in advance) for two weeks at room temperature (RT) in darkness, with solution refreshment on the second day of incubation.

Brain blocks were transferred to solution C for 72 h (solution refreshed after 24 h), then sectioned coronally at 100 μm on a vibratome (OTS-5000 Electron Microscopy Sciences). Sections were collected in 0.1 M PBS, mounted on gelatin-coated slides, and air-dried overnight. Slices were incubated in staining solution (solutions D and E mixed 1:1 and diluted 1:2 with distilled water) for 6 min at RT, rinsed twice in water (5 min each), dehydrated through graded ethanols (50%, 70%, 95%, 100%; 5 min each), cleared with xylene (5 min), cover-slipped with Permount (Fisher Chemical), and stored in darkness at room temperature.

### Spine imaging and 2D morphometry (Golgi-Cox)

**Fig 2:**
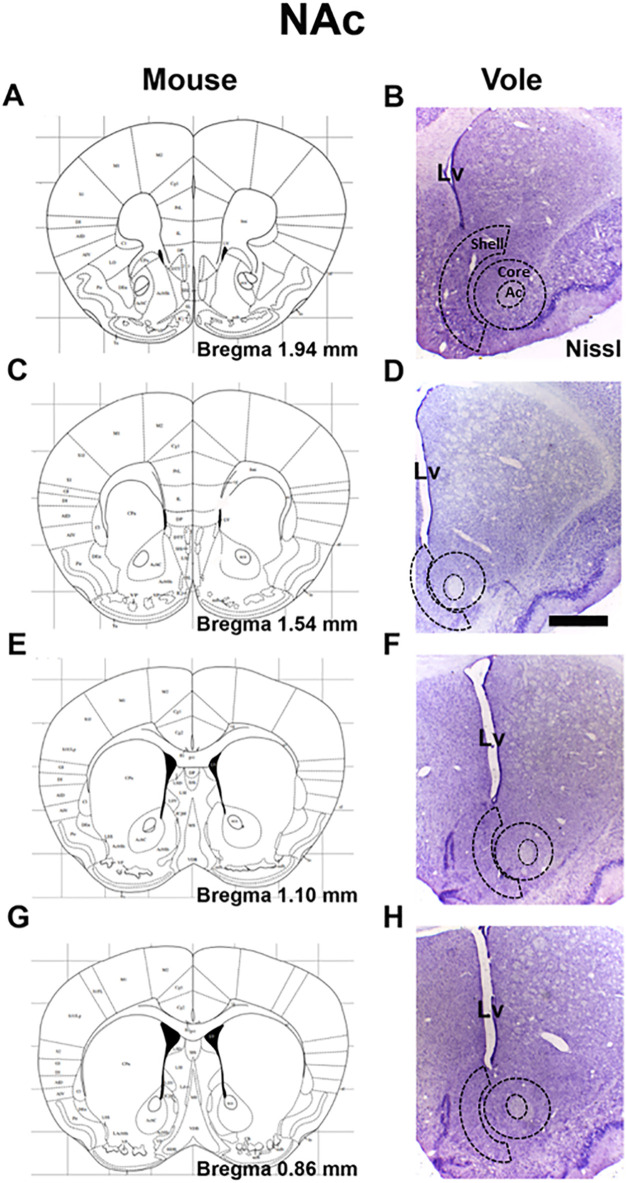
Anatomical references for Golgi-Cox analysis. A, C, E, G. Schematic coronal sections illustrating nucleus accumbens (NAc) core and shell boundaries at representative Bregma levels, adapted from [53] mouse atlas. B, D, F, H. These coordinates guided the selection of female vole brain sections for Golgi-Cox processing. Nissl staining. Ac: anterior commissure. Lv: lateral ventricle. Scale bar: 200 µm.

NAc core and shell boundaries and the Bregma levels used to guide section selection are schematized in Fig 2A, C, E and G (adapted atlas as noted in the legend). These coordinates guided the selection of brain sections to Golgi-Cox processing Fig 2B, D, F and H. Golgi-Cox impregnated coronal sections (100 μm) were imaged under an Olympus BX60 microscope using an immersion 100X oil-immersion objective. For each neuron, secondary dendrites were identified in both hemispheres and examined with fine through-focus to verify a single best-focus plane. To minimize out-of-plane artefacts, we include only dendritic segments oriented approximately parallel to the image plane and exclude segments that traversed sharply in z (e.g., entered/left focus within the 10 μm analysis window). Images were analyzed in Image J/Fiji with a pixel-to-microscope scale bar.

#### Inclusion criteria and blinding.

Only protrusions with a clearly resolvable neck and head (when present) in a single best-focus frame were measured; ambiguous profiles were excluded *a priori*. Spine tracing and subtyping were performed blind to the experimental group by a trained analyst; a second analyst performed periodic cross-checks on randomly selected fields to ensure consistency.

Measurement rules and operational definitions [47]. For each spine, we measured height (H), distance from the dendritic shaft to the distal tip along the neck axis, and the head width (W), maximum diameter of the head (when present), and computed H/W. Subtypes were assigned using a pre-specified decision tree to avoid rule overlap: 1) Branched a single neck giving rise to ≥ 2 protusions; 2) Mushroom W > 0.6 μm, irrespective of H; 3) Filopodiaum: H > 2.0 μm with no bulbous head; 4) Long-thin: H > 1.0 μm and H/W > 1.0 (head present but narrow); 5) Thin H/W > 1.0 μm with H < 1.0 μm and 6) Stubby: H/W ≤ 1.0. Spine density was quantified per 10 μm dendritic segment; percentages were computed as subtype counts divided by total spines per segment. Only spines meeting the inclusion criteria were counted (Fig 4). For spine-subtype analyses, a random subset of five females per group was analyzed and five neurons per NAc were analyzed. C (core = 25 neurons; shell = 25); SE (core = 25; shell = 25); SCM (core = 25; shell = 25).

### Morphological analyses

Dendritic length, branching, and soma diameter were measured using Image J/Fiji software analysis from images taken with a 40X objective. Dendritic length was traced from soma to segment terminus. Because prairie vole MSNs exhibit limited arborization (rarely beyond third-order branches; Fig 5A), branching was quantified as first-order bifurcations. Soma diameter was measured using the microscope scale bar as a reference. Sample sizes: C = 7 females five neurons per NAc region were analyzed (core = 35 neurons; shell = 35); SE = 6 (core = 30; shell = 30) and SCM = 6 (core = 30; shell = 30).

**Fig 3:**
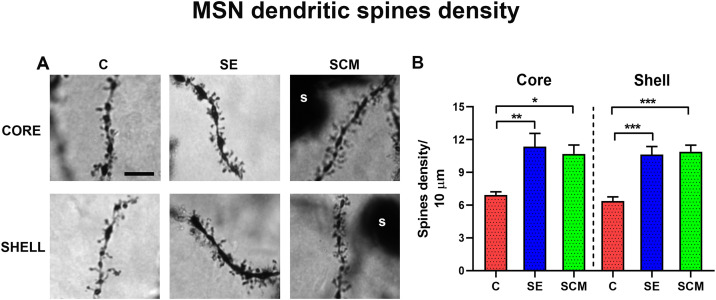
Dendritic-spine density in NAc core and shell. A. Representative Golgi-Cox micrographs (100x; scale bar = 5 µm) of medium spiny neurons (MSNs) from Control (C), Social Exposure (SE), and Social cohabitation with mating (SCM) groups. s: neuron soma. B. Quantification of spine density in secondary dendrites (10 μm segments). Data was analyzed with One-way ANOVA, followed by Bonferroni’s tests. * *p* < 0.05; ** *p* < 0.01; *** *p* < 0.001 vs C.

**Fig 4:**
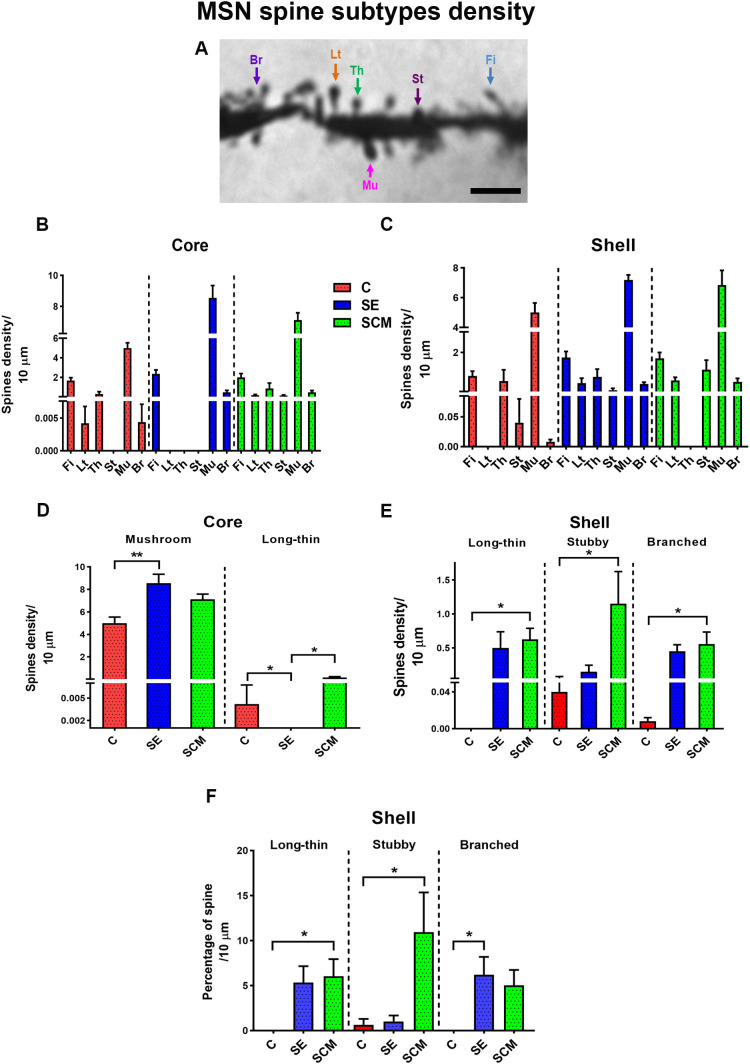
Spine-subtype distribution in MSN. A. Representative MSN dendritic segment from a SE female showing the different spine types quantified. Left to right: Br: branched. Lt: long-thin. Th: thin. Mu: mushroom. St: stubby. Fi: filopodia. Scale bar: 3 µm. B, C. Stacked-bar depiction of absolute counts for six spine subtypes (Fi, Lt, Th, St, Mu, Br) in core (B) and shell (C). D, E. Density (spines/10 µm) of spine subtypes exhibiting significant group effects. F. Percentage of spine subtypes in the shell region. One-way ANOVA followed by Bonferroni’s *post hoc* test. * *p =* 0.05; ** *p =* 0.01. Only categories with significant differences are graphed. Control (C), Social exposure (SE), and social cohabitation with mating (SCM) groups.

**Fig 5:**
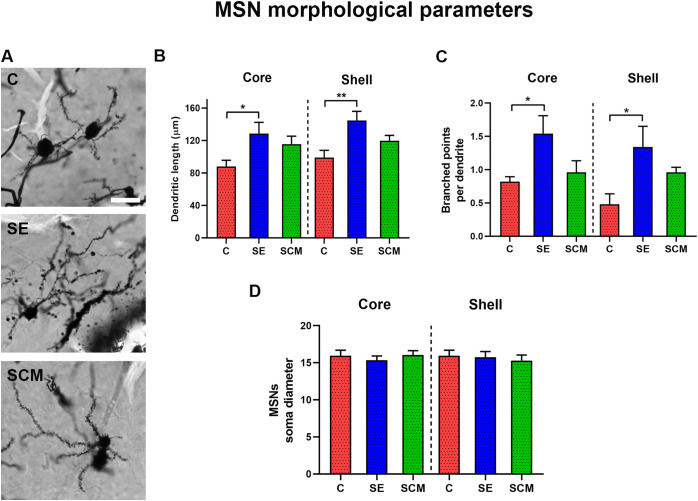
Morphometric parameters of NAc MSNs. A. Representative MSN from each group (Control (C), Social exposure (SE), and Social cohabitation with mating (SCM), scale bar = 20 µm. B-D. Mean dendritic length (B), number of branch points (arborization; C), and soma diameter (D). One-way ANOVA followed by Bonferroni’s *post hoc* test. * *p =* 0.05; ** *p =* 0.01 vs. C. Scale bar: 15 µm.

### Statistical analysis

Data were analyzed in GraphPad Prism 8 (GraphPad Software, San Diego, CA, USA). Normality was assessed with the Shapiro-Wilk test. Sexual behavior variables did not have a normal distribution; therefore, median, minimum, maximum, range, and 25th/75th percentiles are reported in Table 1. Dendritic spine density and morphological parameters had a normal distribution. They were analyzed with one-way ANOVA followed by Bonferroni’s *post hoc* tests. Data are presented as means ± SEM; p ≤ 0.05 was considered significant.

## Results

### Mating verification and dendritic spine density

To confirm sexual interaction in the SCM condition, we quantified male sexual behavior during the first hour (Table 1); all males ejaculated at least once, verifying that mating occurred at the star of the session. Representative Golgi-Cox micrographs of MSN in the NAc core and shell are shown for each experimental group in Fig 3A.

#### Total dendritic-spine density.

Across the NAc, both male sensory exposure and cohabitation with mating were associated with higher MSN spine density relative to controls (Fig 3B). In the core, one-way ANOVA detected a significant group effect on total spine density F _(2,12)_ = 7.78, p = 0.0068, ƞ^2^ = 0.56; *post hoc* test showed increased in SE (p = 0.009*)* and SCM (p = 0.027*)* vs. C*.* In the shell, spine density also differed among experimental groups F_(2,12)_ = 23.27, p < 0.0001, ƞ^2^ = 0.79, with SE and SCM females showed again higher density than C (p =* *0.0003; p = 0.0002; respectively, Fig 3B and Table 2).

**Table 2:** Summary of the differences in MSN in the total spine density, spine subtype density, and morphological parameters in the NAc.

Synaptic parameter	NAc subarea	
**MSN morphological parameter**	**Core**	**Shell**
**Total spine density**	**↑↑ SE (vs C)**	**↑↑↑ SE (vs C)**
	**↑ SCM (vs C)**	**↑↑↑ SCM (vs C)**
**Spine subtype density**		
**Long-thin**	**↑ C (vs SE)**	**↑ SCM (vs C)**
	**↑ SCM (vs SE)**	**ns**
**Stubby**	**ns**	**↑ SCM (vs C)**
**Mushroom**	**↑↑ SE (vs C)**	**ns**
**Branched**	**ns**	**↑ SCM (vs C)**
**Dendritic length**	**↑ SE (vs C)**	**↑↑ SE (vs C)**
**Arborization (branched points)**	**↑ SE (vs C)**	**↑ SE (vs C)**
**Soma diameter**	**ns**	**ns**

Arrows indicate the direction of change relative between groups within each subregion. The number of arrows encodes the adjusted p-value from one-way ANOVA with Bonferroni post-hoc test comparisons:

**↑** increase, p < 0.05 (adjusted).

**↑↑ **increase, p < 0.01 (adjusted).

**↑↑↑ **increase, p < 0.001 (adjusted).

n.s.: not significant after adjustment.

### Spine-subtype composition and density

To determine whether specific spine classes drove these effects, we classified spines as branched, mushroom, filopodia, long-thin, thin, or stubby [42]. Fig 4A illustrates subtype identification on a representative secondary dendrite. Fig 4B, C provides stacked summaries of subtype distribution in core and shell, respectively. Fig 4D, E shows density (spines per 10 μm) by subtype; Fig 4F reports percentages.

In the NAc core, two types varied by group (Fig 4D): mushroom spines, F _(2,12)_ = 8.22, p = 0.006, ƞ^2^ = 0.57 and long-thin spines, F _(2,12)_ = 4.99, p = 0.026, ƞ^2^ = 0.45. Neurons from the SE group had more mature mushroom neurons than C females (p = 0.005). Conversely, C and SCM prairie voles showed higher long-thin densities than SE (p = 0.046 and p = 0.042). Table 2.

Densities of filopodia, thin, stubby and branched spines did not differ. Similarly, spine-type percentages in the core did not vary significantly across groups.

By contrast, the shell showed selective increase in long-thin, stubby and branched spines densities (Fig 4E and Table 2; long-thin: F_(2,12)_ = 5.33, p = 0.022, ƞ^2^ = 0.47; stubby: F_(2,12)_ = 4.97, p = 0.026, ƞ^2^ = 0.45; branched: F_(2,12)_ = 4.8, p = 0.029, ƞ^2^ = 0.44. *Post-hoc* test indicated that females from the SCM had higher density than C for each subtype (p = 0.04, 0.045, 0.043). Filopodia, thin, and mushroom densities did not differ.

For percentage (Fig 4F), long-thin, stubby, and branched spines varied across group (F_(2,12)_= 4.75, p = 0.03, ƞ^2^ = 0.44; F_(2,12)_ = 5.05, p = 0.025, ƞ^2^ = 0.46; F_(2,12)_ = 4.74, p = 0.03, ƞ^2^ = 0.44). *Post hoc* analysis reported that the percentage in SCM exceeded C for long-thin and stubby (p = 0.046, p = 0.048), whereas SE exceeded C for branched (p = 0.039). Percentages of filopodia, thin and mushroom did not differ.

### MSNs dendritic morphology

Representative neurons in the NAc are shown in Fig 5A. Dendritic length increased with SE female in comparison to C voles in both subregions: core, F_(2,16)_ = 4.05, p = 0.037, ƞ^2^ = 0.34; shell, F_(2,16)_= 6.39, p = 0.009, ƞ^2^ = 0.44. Fig 5B and Table 2. Arborization (branch points) likewise increased with SE: core F_(2,16)_= 4.4, p = 0.03, ƞ^2^ = 0.35 (SE > C, p = 0.035); shell, F_(2,16)_= 4.72, p = 0.02, ƞ^2^ = 0.37 (SE > C, p = 0.02, Fig 5C and Table 2). Soma diameter did not differ among groups in either region. Fig 5D.

## Discussion

Sexual activity and pair-bonding are motivated behaviors orchestrated by decision-making network that includes the social-behavior network and the mesolimbic reward system [7]. Within this circuitry, the NAc regulates pair-bond formation, maintenance, and disruption in prairie voles, functional coupling between the NAc and medial prefrontal cortex drives affiliative behavior [28,54], and NAc connectivity remodels across bonding stages [55].

1 Global spinogenesis observed with male cues and mating

Consistent with links between spine number and synaptic plasticity [48,56], both male sensory exposure and social cohabitation with mating were associated with higher total spine density in NAc core and shell (Fig 3 and Table 2). Comparable effects have been reported after sexual experience in female Syrian hamsters, where increases were confined to the core [57,58]. Notably, SE females, who did not form pair bonds, also exhibited spine gains, indicating that exposure to male cues can coincide with accumbal spinogenesis, paralleling cue-dependent adult neurogenesis in prairie voles [9,59]. We therefore interpret SE-related spine increases as compatible with social-cue-evoked plasticity.

2 Region-specific functional implications

NAc shell plasticity has been implicated in partner-preference learning: μ-opioid receptor blockade in the dorsomedial shell prevents partner preference without affecting mating [60]. Whereas κ-opioid antagonism selectively increases aggression during bond maintenance [3]. Dopamine contributes similarly: activation of D2 receptors in the rostral shell promotes partner preference, whereas D1 receptors in both subregions support selective aggression [24,61–63]. In this context, the concomitant core and shell spine increases we observed may support both formation (shell) and consolidation/maintenance (core and shell) of the bond. We present this as a plausible alignment with prior work rather than a definitive mechanistic assignment.

3 Spine-subtype remodeling

Subtype analyses refine this picture. SE females showed more mushroom spines in the core and fewer long-thin spines, a pattern consistent with stabilization of synapses that encoding salient social cues [64,65]. However, because locomotor and arousal parameters were not quantified, we cannot distinguish incentive-salience-related plasticity from the effects of general behavioral activation. We therefore frame prior cue-only studies (e.g., on sexual receptivity and neurogenesis) as convergent evidence, rather than proof, of incentive encoding in our dataset.

SCM females exhibited higher densities of long-thin, stubby, and branched spines in the NAc shell and a greater proportion of long-thin and stubby subtypes. Long-thin spines are dynamic and can transition to mushroom forms; stubby spines are characteristic of heightened excitatory drive; branched spines represent mature, multiply innervated sites [43,64,66]. Together, these patterns suggest diversified synaptic remodeling associated with mating and nascent bonding. Similar subtype-specific changes occur with other motivated behaviors (sexual experience or aggression) in Syrian hamsters [58,67,68] and mating-related increases in mushroom spines have been reported in male rats [69]. Related morphologies also arise after exposure to addictive drugs or hormonal fluctuations [38,68,70], underscoring shared structural motifs across motivational states.

4 Dendritic growth during male-cue exposure

In our cohort, male exposure alone coincide with longer dendrites and increased branching in both NAc subregions. Because dendritic growth expand potential synaptic territory and arborization scales with synapse formation [71], SE may prepare circuits for subsequent plasticity. Crucially, in the absence of behavioral quantification, increased dendritic complexity could also reflect elevated locomotion or exploratory drive.

5 Candidate molecular mediators

Spinogenesis is orchestrated by scaffold proteins such as Postsynaptic density-95 (PSD-95), Shank and Homer [72–74]. Our unpublished observations of higher PSD-95 density in SE and SCM groups parallel the spine increase. Modulation by estradiol, dopamine, oxytocin, and opioids peptides is also well supported [37,75–78]. Although all females were ovariectomized and received identical estradiol priming, group differences persisted, suggesting contributions from non-hormonal pathways. Future work using intact females, receptor-specific manipulations (e.g., chemogenetics), or targeted gene perturbations (e.g., CRISPR interference) will be needed to delineate causal pathways.

### Limitations and future directions

Behavioral context: Partner-preference testing was not performed in this cohort; direct linking spine metrics to individual bonding strength remains essential. Moreover, behavior during the 6 h session was not quantified; therefore, distinctions between the incentive process and motor activation are provisional. Future work will implement blinded ethological scoring and/or automated tracking.

Temporal scope: The 48 h endpoint captures early pair-bond formation. Longitudinal studies should test persistence, regression or maturation of spine changes during bond maintenance and after partner loss.

Physiological relevance: Patch-clamp recordings will determine whether newly spines translate into altered excitatory drive and MSN output during social interactions.

Imaging approach: morphometry was performed on 2D bright-field Golgi-Cox images rather than 3D confocal stacks. Although 2D Golgi-Cox is established for NAc MSN analyses and has revealed robust group differences, out-of-plane geometry can bias subtype assignment. We mitigated this by restricting analyses to in-plane dendrites, requiring a clearly resolvable neck/head in a single best-focus frame, applying a rule-based decision tree for subtyping, and performing blinded analyses. Even so, future studies will pair Golgi-Cox with confocal z-stacks or super-resolution and automated spine tracking to validate subtype distributions across focal planes.

## Conclusion

Exposure to male sensory cues without physical contact coincides with increased mature mushroom spine density and greater dendritic complexity in the NAc core and shell of female prairie voles, whereas cohabitation with mating is associated with subtype-specific remodeling, particularly increased long-thin, stubby, and branched spines in the shell- suggestive of region-specific adaptations relevant to bond consolidation. We propose that the NAc integrates social exposure and mating experience as motivational signals that may stabilize social monogamy, while emphasizing that causal interpretations await behavioral quantification and mechanistic tests. Dissecting the relative roles of estradiol, dopamine, oxytocin, and opioid peptides in NAc spinogenesis remains a pivotal objective for future studies.

## Supporting information

S1 File Data base Castro et al (1). (PDF)
