# Supplementary material for: Social experience and social cohabitation with mating promote spinogenesis in the nucleus accumbens of adult female prairie voles (Microtus ochrogaster)
Source: PLoS One. 2025 Nov 3;20(11):e0335626. doi: 10.1371/journal.pone.0335626 (PMC12582430; doi:10.1371/journal.pone.0335626)
Supplement: S1 File — (PDF) [file pone.0335626.s001.pdf]

Data base Castro et al., 2025

Control
Females 48h
NAcc
CORE
Spines density

	Animal	Length	Width	Ratiio L/W	Subtype	Number
1	C2h-acc-c1-co-n2.tif	0.9702	0.8912	1.0886	MUSH	1
3	C2h-acc-c1-co-n2.tif	1.4038	0.7767	1.8074	MUSH	1
5	C2h-acc-c1-co-n2.tif	1.0554	0.6646	1.5880	MUSH	1
7	C2h-acc-c1-co-n2.tif	1.6291	0.7074	2.3029	MUSH	1
9	C2h-acc-c1-co-n2.tif	2.5259	1.0365	2.4370	FILO	1
11	C2h-acc-c1-co-n2.tif	2.2402	0.6061	3.6961	FILO	1
						**6**
13	C2h-acc-c1-co-n3.tif	1.2407	0.8954	1.3856	MUSH	1
15	C2h-acc-c1-co-n3.tif	1.8741	0.7343	2.5522	MUSH	1
17	C2h-acc-c1-co-n3.tif	2.5637	0.8684	2.9522	FILO	1
19	C2h-acc-c1-co-n3.tif	2.0005	0.8779	2.2787	FILO	1
21	C2h-acc-c1-co-n3.tif	1.5461	0.8344	1.8529	MUSH	1
23	C2h-acc-c1-co-n3.tif	1.0985	0.8295	1.3243	MUSH	1
25	C2h-acc-c1-co-n3.tif	1.3944	0.7455	1.8704	MUSH	1
27	C2h-acc-c1-co-n3.tif	0.6818	0.7741	0.8808	MUSH	1
29	C2h-acc-c1-co-n3.tif	1.14	0.8182	1.3933	MUSH	1
31	C2h-acc-c1-co-n3.tif	0.9556	1.0494	0.9106	MUSH	1
						**10**
33	C2h-acc-c3-co-n6tif.tif	1.5779	1.0255	1.5387	MUSH	1
35	C2h-acc-c3-co-n6tif.tif	1.7296	0.6364	2.7178	MUSH	1
37	C2h-acc-c3-co-n6tif.tif	1.2035	0.8636	1.3936	MUSH	1
39	C2h-acc-c3-co-n6tif.tif	0.9791	1.2605	0.7768	MUSH	1
41	C2h-acc-c3-co-n6tif.tif	0.7767	0.7767	1.0000	MUSH	1
43	C2h-acc-c3-co-n6tif.tif	1.0918	0.638	1.7113	MUSH	1
45	C2h-acc-c3-co-n6tif.tif	2.002	0.9091	2.2022	FILO	1
47	C2h-acc-c3-co-n6tif.tif	1.4098	0.8054	1.7504	MUSH	1

49	C2h-acc-c3-co-n6tif.tif	1.2239	0.8779	1.3941	MUSH	1
51	C2h-acc-c1-co-n6tif.tif	1.9969	1.001	1.9949	MUSH	1
						**10**
63	C2h-acc-c2-co-n11.tif	1.3644	1.2735	1.0714	MUSH	1
65	C2h-acc-c2-co-n11.tif	1.8323	0.8182	2.2394	MUSH	1
67	C2h-acc-c2-co-n11.tif	1.8458	1.1508	1.6039	MUSH	1
69	C2h-acc-c2-co-n11.tif	0.9791	0.6711	1.4589	MUSH	1
71	C2h-acc-c1-sh-n1.tif	2.3043	0.7273	3.1683	FILO	1
						**5**
131	C4h-acc-c1-co-n6.tif	2.2768	1.0909	2.0871	FILO	1
133	C4h-acc-c1-co-n6.tif	2.0046	1.2	1.6705	FILO	1
135	C4h-acc-c1-co-n6.tif	1.6971	0.9371	1.8110	MUSH	1
137	C4h-acc-c1-co-n6.tif	1.8547	0.8731	2.1243	MUSH	1
139	C4h-acc-c1-co-n6.tif	2.058	1.1003	1.8704	FILO	1
141	C4h-acc-c1-co-n6.tif	1.7279	1.1615	1.4876	MUSH	1
143	C4h-acc-c1-co-n6.tif	2.0555	0.9371	2.1935	FILO	1
						**7**
145	C4h-acc-c1-co-n8.tif	1.2785	1.3016	0.9823	MUSH	1
147	C4h-acc-c1-co-n8.tif	1.4972	0.9231	1.6219	MUSH	1
149	C4h-acc-c1-co-n8.tif	1.8797	1.8797	1.0000	MUSH	1
151	C4h-acc-c1-co-n8.tif	2.0009	0.7484	2.6736	FILO	1
						**4**
153	C4h-acc-c1-co-n9.tif	1.9434	1.2439	1.5623	MUSH	1
155	C4h-acc-c1-co-n9.tif	1.9196	0.7954	2.4134	MUSH	1
157	C4h-acc-c1-co-n9.tif	0.7931	0.8533	0.9295	MUSH	1
159	C4h-acc-c1-co-n9.tif	0.9201	0.7576	1.2145	MUSH	1
161	C4h-acc-c1-co-n9.tif	2.0305	0.8182	2.4817	FILO	1
163	C4h-acc-c1-co-n9.tif	1.7275	0.9335	1.8506	MUSH	1
						**6**
165	C4h-acc-c1-co-n9c.tif	1.2239	1.0641	1.1502	MUSH	1
167	C4h-acc-c1-co-n9c.tif	1.0215	1.0215	1.0000	MUSH	1
169	C4h-acc-c1-co-n9c.tif	1.9519	0.9091	2.1471	MUSH	1
171	C4h-acc-c1-co-n9c.tif	1.3674	0.6757	2.0237	MUSH	1
173	C4h-acc-c1-co-n9c.tif	2.8448	1.7487	1.6268	FILO	1
175	C4h-acc-c1-co-n9c.tif	2.2622	0.9271	2.4401	FILO	1

177	C4h-acc-c1-co-n9c.tif	1.5561	0.6953	2.2380	MUSH	1
179	C4h-acc-c1-co-n9c.tif	2.2314	0.9326	2.3927	FILO	1
						**8**
181	C4h-acc-c1-co-n10.tif	1.085	0.6653	1.6308	MUSH	1
183	C4h-acc-c1-co-n10.tif	1.0365	0.8381	1.2367	MUSH	1
185	C4h-acc-c1-co-n10.tif	1.6197	1.4057	1.1522	MUSH	1
187	C4h-acc-c1-co-n10.tif	1.2098	0.7931	1.5254	MUSH	1
189	C4h-acc-c1-co-n10.tif	0.9908	0.8381	1.1822	MUSH	1
191	C4h-acc-c1-co-n10.tif	1.4485	0.7931	1.8264	MUSH	1
193	C4h-acc-c1-co-n10.tif	1.1346	0.687	1.6515	MUSH	1
						**7**
255	C5h-acc-c1-co-n2.tif	2.9264	0.71	4.1217	FILO	1
257	C5h-acc-c1-co-n2.tif	2.2291	0.6757	3.2989	FILO	1
259	C5h-acc-c1-co-n2.tif	2.2453	1.1069	2.0285	FILO	1
261	C5h-acc-c1-co-n2.tif	1.0164	0.6555	1.5506	MUSH	1
263	C5h-acc-c1-co-n2.tif	2.1594	0.5454	3.9593	FILO	1
						**5**
265	C5h-acc-c1-co-n3.tif	1.5434	0.7741	1.9938	MUSH	1
267	C5h-acc-c1-co-n3.tif	2.0555	1.0928	1.8809	FILO	1
269	C5h-acc-c1-co-n3.tif	0.7767	1.0611	0.7320	MUSH	1
271	C5h-acc-c1-co-n3.tif	2.0914	0.9717	2.1523	FILO	1
273	C5h-acc-c1-co-n3.tif	1.963	1.1642	1.6861	MUSH	1
275	C5h-acc-c1-co-n3.tif	1.001	0.5183	1.9313	THIN	1
277	C5h-acc-c1-co-n3.tif	2.0635	1.6824	1.2265	FILO	1
279	C5h-acc-c1-co-n3.tif	0.9193	1.2035	0.7639	MUSH	1
						**8**
281	C5h-acc-c1-co-n4.tif	1.7294	0.6653	2.5994	MUSH	1
283	C5h-acc-c1-co-n4.tif	1.7591	0.7527	2.3371	MUSH	1
285	C5h-acc-c1-co-n4.tif	1.6994	0.6653	2.5543	MUSH	1
287	C5h-acc-c1-co-n4.tif	1.8305	0.6694	2.7345	MUSH	1
289	C5h-acc-c1-co-n4.tif	1.0641	1.0641	1.0000	MUSH	1
291	C5h-acc-c1-co-n4.tif	0.7203	0.6098	1.1812	MUSH	1
						**6**
343	C5h-acc-c2-co-n3.tif	2.9716	0.7287	4.0779	FILO	1
345	C5h-acc-c2-co-n3.tif	1.2239	0.7524	1.6267	MUSH	1

347	C5h-acc-c2-co-n3.tif	2.3146	0.8131	2.8466	FILO	1
349	C5h-acc-c2-co-n3.tif	1.3182	0.5821	2.2646	THIN	1
351	C5h-acc-c2-co-n3.tif	2.1709	0.9589	2.2639	FILO	1
353	C5h-acc-c2-co-n3.tif	0.9458	0.5821	1.6248	THIN	1
355	C5h-acc-c2-co-n3.tif	1.2735	0.6364	2.0011	MUSH	1
357	C5h-acc-c2-co-n3.tif	1.4952	0.5909	2.5304	THIN	1
359	C5h-acc-c2-co-n3.tif	0.6878	0.575	1.1962	THIN	1
195						**9**
375	C6h-acc-c1-co-n1.tif	0.9717	1.0804	0.8994	MUSH	1
377	C6h-acc-c1-co-n1.tif	1.9735	0.3636	5.4277	THIN	1
379	C6h-acc-c1-co-n1.tif	2.0459	1.0494	1.9496	FILO	1
381	C6h-acc-c1-co-n1.tif	1.4208	0.8743	1.6251	MUSH	1
383	C6h-acc-c1-co-n1.tif	0.9632	0.9091	1.0595	MUSH	1
385	C6h-acc-c1-co-n1.tif	0.8357	0.936	0.8928	MUSH	1
387	C6h-acc-c1-co-n1.tif	1.8323	1.0928	1.6767	MUSH	1
						**7**
389	C6h-acc-c1-co-n2.tif	1.8073	0.8	2.2591	MUSH	1
391	C6h-acc-c1-co-n2.tif	1.2479	0.7453	1.6744	MUSH	1
393	C6h-acc-c1-co-n2.tif	1.8019	0.9664	1.8645	MUSH	1
395	C6h-acc-c1-co-n2.tif	1.5251	0.71	2.1480	MUSH	1
397	C6h-acc-c1-co-n2.tif	1.5949	0.7152	2.2300	MUSH	1
399	C6h-acc-c1-co-n2.tif	2.0641	1.1142	1.8525	FILO	1
401	C6h-acc-c1-co-n2.tif	0.9091	1	0.9091	MUSH	1
403	C6h-acc-c1-co-n2.tif	1.5314	1.0222	1.4981	MUSH	1
						**8**
405	C6h-acc-c1-co-n3.tif	1.2605	0.9833	1.2819	MUSH	1
407	C6h-acc-c1-co-n3.tif	1.0305	0.9091	1.1335	MUSH	1
409	C6h-acc-c1-co-n3.tif	1.0215	1.1097	0.9205	MUSH	1
411	C6h-acc-c1-co-n3.tif	1.5007	1.1097	1.3523	MUSH	1
413	C6h-acc-c1-co-n3.tif	1.3824	1.1472	1.2050	MUSH	1
415	C6h-acc-c1-co-n3.tif	0.8826	1.0602	0.8325	MUSH	1
417	C6h-acc-c1-co-n3.tif	1.1818	1.118	1.0571	MUSH	1
						**7**
419	C6h-acc-c1-co-n4.tif	1.511	1.0728	1.4085	MUSH	1
421	C6h-acc-c1-co-n4.tif	1.667	0.9791	1.7026	MUSH	1

423	C6h-acc-c1-co-n4.tif	1.1931	1.0092	1.1822	MUSH	1
425	C6h-acc-c1-co-n4.tif	1.3674	0.8743	1.5640	MUSH	1
427	C6h-acc-c1-co-n4.tif	1.9938	1.2214	1.6324	MUSH	1
						**5**
429	C6h-acc-c1-co-n6.tif	1.2572	1.2	1.0477	MUSH	1
431	C6h-acc-c1-co-n6.tif	2.8009	1.1003	2.5456	FILO	1
433	C6h-acc-c1-co-n6.tif	1.8906	1.0947	1.7270	MUSH	1
435	C6h-acc-c1-co-n6.tif	1.5548	1.0092	1.5406	MUSH	1
437	C6h-acc-c1-co-n6.tif	1.9285	1.0804	1.7850	MUSH	1
439	C6h-acc-c1-co-n6.tif	1.6527	1.2662	1.3052	MUSH	1
441	C6h-acc-c1-co-n6.tif	1.0602	1.2506	0.8478	MUSH	1
						**7**
477	C6h-acc-c2-co-n2.tif	1.0611	0.9102	1.1658	MUSH	1
479	C6h-acc-c2-co-n2.tif	1.6294	0.8576	1.9000	MUSH	1
481	C6h-acc-c2-co-n2.tif	1.6141	0.8684	1.8587	MUSH	1
483	C6h-acc-c2-co-n2.tif	0.9204	0.7115	1.2936	MUSH	1
485	C6h-acc-c2-co-n2.tif	1.7404	0.7951	2.1889	MUSH	1
487	C6h-acc-c2-co-n2.tif	0.8636	0.8636	1.0000	MUSH	1
489	C6h-acc-c2-co-n2.tif	1.2928	1.2856	1.0056	MUSH	1
						**7**
491	C6h-acc-c2-co-n4.tif	2.2388	0.9102	2.4597	FILO	1
493	C6h-acc-c2-co-n4.tif	1.6873	1.0062	1.6769	MUSH	1
495	C6h-acc-c2-co-n4.tif	1.7308	1.1364	1.5231	MUSH	1
497	C6h-acc-c2-co-n4.tif	1.0804	1.2077	0.8946	MUSH	1
499	C6h-acc-c2-co-n4.tif	1.4237	1.0928	1.3028	MUSH	1
501	C6h-acc-c2-co-n4.tif	1.5909	0.8093	1.9658	MUSH	1
503	C6h-acc-c2-co-n4.tif	1.2365	0.936	1.3210	MUSH	1
						**7**
505	C7h-acc-c2-co-n1-.tif	1.5381	0.71	2.1663	MUSH	1
507	C7h-acc-c2-co-n1-.tif	1.4841	0.9589	1.5477	MUSH	1
509	C7h-acc-c2-co-n1-.tif	1.6477	0.8357	1.9716	MUSH	1
511	C7h-acc-c2-co-n1-.tif	0.8344	0.6711	1.2433	MUSH	1
513	C7h-acc-c2-co-n1-.tif	0.6428	0.8093	0.7943	MUSH	1
515	C7h-acc-c2-co-n1-.tif	0.6182	0.782	0.7905	MUSH	1
517	C7h-acc-c2-co-n1-.tif	0.9545	1.0994	0.8682	MUSH	1

519	C7h-acc-c2-co-n1-.tif	1.5454	0.638	2.4223	MUSH	1
						**8**
521	C7h-acc-c2-co-n2-.tif	1.4609	0.9271	1.5758	MUSH	1
523	C7h-acc-c2-co-n2-.tif	1.2407	1.0215	1.2146	MUSH	1
525	C7h-acc-c2-co-n2-.tif	1.4467	1.3016	1.1115	MUSH	1
527	C7h-acc-c2-co-n2-.tif	2.0373	0.8965	2.2725	FILO	1
529	C7h-acc-c2-co-n2-.tif	0.9147	0.7619	1.2006	MUSH	1
531	C7h-acc-c2-co-n2-.tif	1.8741	0.9091	2.0615	MUSH	1
533	C7h-acc-c2-co-n2-.tif	1.7249	0.8743	1.9729	MUSH	1
						**7**
535	C7h-acc-c2-co-n3a-.tif	2.0815	0.6331	3.2878	FILO	1
537	C7h-acc-c2-co-n3a-.tif	1.3824	0.9632	1.4352	MUSH	1
539	C7h-acc-c2-co-n3a-.tif	2.0454	0.9556	2.1404	FILO	1
541	C7h-acc-c2-co-n3a-.tif	1.7038	1.2035	1.4157	MUSH	1
543	C7h-acc-c2-co-n3a-.tif	2.3043	0.5492	4.1957	FILO	1
						**5**
545	C7h-acc-c2-co-n5-.tif	1.3757	1.0164	1.3535	MUSH	1
547	C7h-acc-c2-co-n5-.tif	1.4237	0.7187	1.9809	MUSH	1
549	C7h-acc-c2-co-n5-.tif	2.05	0.8194	2.5018	FILO	1
551	C7h-acc-c2-co-n5-.tif	1.0699	1.1818	0.9053	MUSH	1
553	C7h-acc-c2-co-n5-.tif	1.3	0.9979	1.3027	MUSH	1
555	C7h-acc-c2-co-n5-.tif	0.8194	1.3461	0.6087	MUSH	1
						**6**
557	C7h-acc-c2-co-n8-.tif	2.4851	0.8455	2.9392	FILO	1
559	C7h-acc-c2-co-n8-.tif	1.0215	1.2035	0.8488	MUSH	1
561	C7h-acc-c2-co-n8-.tif	3.0342	0.8636	3.5134	FILO	1
563	C7h-acc-c2-co-n8-.tif	2.1344	1.0164	2.1000	FILO	1
565	C7h-acc-c2-co-n8-.tif	1.8741	0.71	2.6396	MUSH	1
567	C7h-acc-c2-co-n8-.tif	1.6824	0.9091	1.8506	MUSH	1
						**6**
569	C7h-acc-c2-sh-n4.tif	2.6473	1.6689	1.5863	FILO	
571	C7h-acc-c2-sh-n4.tif	1.7273	1.3636	1.2667	MUSH	
782	C2h-acc-c1-co-n4.tif	1.276	1.3016	0.9803	MUSH	1
784	C2h-acc-c1-co-n4.tif	1.6546	0.6315	2.6201	MUSH	1

786	C2h-acc-c1-co-n4.tif	2.0954	0.6818	3.0733	FILO	1
788	C2h-acc-c1-co-n4.tif	1.0909	0.8131	1.3417	MUSH	1
790	C2h-acc-c1-co-n4.tif	0.6508	0.936	0.6953	MUSH	1
792	C2h-acc-c1-co-n4.tif	1.4374	0.8093	1.7761	MUSH	1
794	C2h-acc-c1-co-n4.tif	1.8193	0.762	2.3875	MUSH	1
796	C2h-acc-c1-co-n4.tif	2.3324	0.5927	3.9352	FILO	1
798	C2h-acc-c1-co-n4.tif	2.1551	0.6115	3.5243	FILO	1
						**9**

**Animal**	**CORE**						
**C2**	**FILO**	**MUSH**	**LONG TH**	**THIN**	**STUBBY**	**BRANCHED**	**TOTAL**
N2	2	4	0	0	0	0	6
N3	2	8	0	0	0	0	10
N6	1	9	0	0	0	0	10
N11	1	4	0	0	0	0	5
N4	3	6	0	0	0	0	9
**C2 average**	**1.8**	**6.2**	**0**	**0**	**0**	**0**	**8**
**C4**	**FILO**	**MUSH**	**LONG TH**	**THIN**	**STUBBY**	**BRANCHED**	**TOTAL**
N6	4	3	0	0	0	0	7
N8	1	3	0	0	0	0	4
N9	1	5	0	0	0	0	6
N9C	3	5	0	0	0	0	8
N10	0	7	0	0	0	0	7
**C4 average**	**1.8**	**4.6**	**0**	**0**	**0**	**0**	**6.4**
**C5**	**FILO**	**MUSH**	**LONG TH**	**THIN**	**STUBBY**	**BRANCHED**	**TOTAL**
N2	4	1	0	0	0	0	5
**N3**	3	4	0	1	0	0	8
N4	0	6	0	0	0	0	6
**N3C2**	3	2	0	4	0	0	9
N							
**C5 average**	**2.5**	**3.25**	**0**	**1.25**	**0**	**0**	**7**
**C6**	**FILO**	**MUSH**	**LONG TH**	**THIN**	**STUBBY**	**BRANCHED**	**TOTAL**

N1	1	5	0	1	0	0	7
N2	1	7	0	0	0	0	8
N3	0	7	0	0	0	0	7
N4	0	5	0	0	0	0	5
N6	1	6	0	0	0	0	7
N2C2	0	7	0	0	0	0	7
N4C2	1	6	0	0	0	0	7
**C6 average**	**0.57**	**6.14**	**0.00**	**0.14**	**0.00**	**0.00**	**6.86**

**C7**	**FILO**	**MUSH**	**LONG TH**	**THIN**	**STUBBY**	**BRANCHED**	**TOTAL**
N1	0	8	0	0	0	0	8
**N2**	1	6	0	0	0	0	7
N3A	3	2	0	0	0	0	5
**N5**	1	5	0	0	0	0	6
N8	3	3	0	0	0	0	6
**C7 average**	**1.6**	**4.8**	**0**	**0**	**0**	**0**	**6.4**

**CORE ANIMAL**	**FILO**	**MUSH**	**LONG TH**	**THIN**	**STUBBY**	**BRANCHED**	**TOTAL**
**C2**	**1.8**	**6.2**	**0**	**0**	**0**	**0**	**8**
**C4**	**1.8**	**4.6**	**0**	**0**	**0**	**0**	**6.4**
**C5**	**2.5**	**3.25**	**0**	**1.25**	**0**	**0**	**7**
**C6**	**0.57**	**6.14**	**0**	**0.14**	**0**	**0**	**6.86**
**C7**	**1.6**	**4.8**	**0**	**0**	**0**	**0**	**6.4**

**Percentage CORE ANIMAL**	**FILO**	**MUSH**	**LONG TH**	**THIN**	**STUBBY**	**BRANCHED**	**TOTAL**
**C2**	22.50	77.50	0.00	0.00	0	0	100
**C4**	28.13	71.88	0.00	0.00	0	0	100
**C5**	35.71	46.43	0.00	17.86	0	0	100
**C6**	8.33	89.58	0.00	2.08	0	0	100
**C7**	25.00	75.00	0.00	0.00	0	0	100

Control
Females 48h
NAcc
SHELL
Spines density

Animal	Length	Width	Ratio L/W	Subtype	Number
C2h-acc-c1-sh-n1.tif	4.0023	1.0375	3.8576	FILO	1
C2h-acc-c1-sh-n1.tif	1.9666	0.8731	2.2524	MUSH	1
C2h-acc-c1-sh-n1.tif	1.9418	0.6508	2.9837	MUSH	1
					**3**
C2h-acc-c2-sh-n3.tif	1.5654	0.7524	2.0805	MUSH	1
C2h-acc-c2-sh-n3.tif	1.4861	1.1792	1.2603	MUSH	1
C2h-acc-c2-sh-n3.tif	2.5491	0.7399	3.4452	FILO	1
C2h-acc-c2-sh-n3.tif	1.5857	1.4039	1.1295	MUSH	1
C2h-acc-c2-sh-n3.tif	0.9491	0.6618	1.4341	MUSH	1
C2h-acc-c2-sh-n3.tif	0.8492	0.9204	0.9226	MUSH	1
C2h-acc-c2-sh-n3.tif	1.5831	1.1254	1.4067	MUSH	1
C2h-acc-c2-sh-n3.tif	0.9371	0.71	1.3199	MUSH	1
					**8**
C2h-acc-c2-sh-n4.tif	1.9096	1.0464	1.8249	MUSH	1
C2h-acc-c2-sh-n4.tif	1.1226	0.8731	1.2858	MUSH	1
C2h-acc-c2-sh-n4.tif	2.07	0.9545	2.1687	FILO	1
C2h-acc-c2-sh-n4.tif	2.7753	0.9371	2.9616	FILO	1
C2h-acc-c2-sh-n4.tif	1.6012	0.9022	1.7748	MUSH	1
C2h-acc-c2-sh-n4.tif	0.8576	0.71	1.2079	MUSH	1
C2h-acc-c2-sh-n4.tif	1.2239	1.2239	1.0000	MUSH	1
C2h-acc-c2-sh-n4.tif	1.0385	0.9326	1.1136	MUSH	1
					**8**
C2h-acc-c3-sh-n5.tif	1.8323	0.8054	2.2750	MUSH	1
C2h-acc-c3-sh-n5.tif	1.2572	0.6182	2.0336	MUSH	1
C2h-acc-c3-sh-n5.tif	1.2872	0.9833	1.3091	MUSH	1
C2h-acc-c3-sh-n5.tif	1.0092	0.9632	1.0478	MUSH	1
C2h-acc-c3-sh-n5.tif	1.5048	1.7308	0.8694	MUSH	1
					**5**
C2h-acc-c3-sh-n6a.tif	1.4446	1.1589	1.2465	MUSH	1

C2h-acc-c3-sh-n6a.tif	1.8741	1.3742	1.3638	MUSH	1
C2h-acc-c3-sh-n6a.tif	1.5693	1.2678	1.2378	MUSH	1
C2h-acc-c3-sh-n6a.tif	2.6399	1.0918	2.4179	FILO	1
C2h-acc-c3-sh-n6a.tif	2.1988	1.0699	2.0551	FILO	1
					**5**
C4h-acc-c1-sh-n2.tif	2.6772	1.1226	2.3848	FILO	1
C4h-acc-c1-sh-n2.tif	2.3465	0.7741	3.0313	FILO	1
C4h-acc-c1-sh-n2.tif	1.6763	0.7187	2.3324	MUSH	1
C4h-acc-c1-sh-n2.tif	2.5201	1.1291	2.2320	FILO	1
C4h-acc-c1-sh-n2.tif	1.6109	0.7767	2.0740	MUSH	1
C4h-acc-c1-sh-n2.tif	1.1589	0.7329	1.5813	MUSH	1
					**6**
C4h-acc-c1-sh-n3.tif	0.9833	0.9371	1.0493	MUSH	1
C4h-acc-c1-sh-n3.tif	0.9589	0.6555	1.4629	MUSH	1
C4h-acc-c1-sh-n3.tif	2.1609	0.8636	2.5022	FILO	1
C4h-acc-c1-sh-n3.tif	0.9091	0.6364	1.4285	MUSH	1
C4h-acc-c1-sh-n3.tif	1.6294	0.9664	1.6861	MUSH	1
C4h-acc-c1-sh-n3.tif	2.3819	1.4799	1.6095	FILO	1
					**6**
C4h-acc-c1-sh-n5.tif	1.2281	1.319	0.9311	MUSH	1
C4h-acc-c1-sh-n5.tif	1.0918	1.1818	0.9238	MUSH	1
C4h-acc-c1-sh-n5.tif	1.2348	0.9193	1.3432	MUSH	1
C4h-acc-c1-sh-n5.tif	1.2727	1	1.2727	MUSH	1
C4h-acc-c1-sh-n5.tif	1.0494	1.3119	0.7999	MUSH	1
					**5**
C4h-acc-c1-sh-n7.tif	1.9844	1.0928	1.8159	MUSH	1
C4h-acc-c1-sh-n7.tif	2.4738	1.1254	2.1982	FILO	1
C4h-acc-c1-sh-n7.tif	1.4374	1.4374	1.0000	MUSH	1
C4h-acc-c1-sh-n7.tif	1.4545	1.001	1.4530	MUSH	1
C4h-acc-c1-sh-n7.tif				STUB	1
C4h-acc-c1-sh-n7.tif	1.711	1.1003	1.5550	MUSH	1
C4h-acc-c1-sh-n7.tif	0.9728	0.9326	1.0431	MUSH	1
					**7**
C4h-acc-c1-sh-n8a.tif	2.5713	1.2727	2.0204	FILO	1
C4h-acc-c1-sh-n8a.tif	1.6546	0.7847	2.1086	MUSH	1

C4h-acc-c1-sh-n8a.tif	2.3092	0.646	3.5746	FILO	1
C4h-acc-c1-sh-n8a.tif	1.9519	0.8743	2.2325	MUSH	1
C4h-acc-c1-sh-n8a.tif	1.5191	1.6122	0.9423	MUSH	1
C4h-acc-c1-sh-n8a.tif	0.5909	0.7938	0.7444	MUSH	1
C4h-acc-c1-sh-n8a.tif	0.7524	0.9371	0.8029	MUSH	1
					**7**
C5h-acc-c1-sh-n3.tif	1.22	0.4791	2.5464	THIN	1
C5h-acc-c1-sh-n3.tif	1.5757	0.7132	2.2093	MUSH	1
C5h-acc-c1-sh-n3.tif	1.2272	0.5151	2.3825	THIN	1
C5h-acc-c1-sh-n3.tif	1.085	0.6996	1.5509	MUSH	1
C5h-acc-c1-sh-n3.tif	1.2189	0.8243	1.4787	MUSH	1
C5h-acc-c1-sh-n3.tif	1.0926	0.553	1.9758	THIN	1
					**6**
C5h-acc-c1-sh-n4.tif	0.9091	1.001	0.9082	MUSH	1
C5h-acc-c1-sh-n4.tif	0.936	0.778	1.2031	MUSH	1
C5h-acc-c1-sh-n4.tif	1.0602	0.9458	1.1210	MUSH	1
C5h-acc-c1-sh-n4.tif	1.2808	0.6938	1.8461	MUSH	1
C5h-acc-c1-sh-n4.tif	0.9833	0.5926	1.6593	THIN	1
C5h-acc-c1-sh-n4.tif	1.2638	0.6331	1.9962	MUSH	1
					**6**
C5h-acc-c1-sh-n5.tif	1.7334	0.7666	2.2612	MUSH	1
C5h-acc-c1-sh-n5.tif	2.3667	1.2076	1.9598	FILO	1
C5h-acc-c1-sh-n5.tif	1.2435	0.6015	2.0673	MUSH	1
C5h-acc-c1-sh-n5.tif	0.6	0.7336	0.8179	MUSH	1
C5h-acc-c1-sh-n5.tif	0.7429	0.5571	1.3335	THIN	1
C5h-acc-c1-sh-n5.tif	0.6646	0.6	1.1077	THIN	1
					**6**
C5h-acc-c1-sh-n6.tif	1.3111	0.5183	2.5296	THIN	1
C5h-acc-c1-sh-n6.tif	1.642	0.5143	3.1927	THIN	1
C5h-acc-c1-sh-n6.tif	1.4003	0.5183	2.7017	THIN	1
C5h-acc-c1-sh-n6.tif	1.2856	0.6115	2.1024	MUSH	1
C5h-acc-c1-sh-n6.tif	0.6833	0.5926	1.1531	THIN	1
C5h-acc-c1-sh-n6.tif	1.6122	0.6182	2.6079	MUSH	1
C5h-acc-c1-sh-n6.tif	1.491	0.7524	1.9817	MUSH	1
					**7**

C5h-acc-c2-sh-n3.tif	1.711	0.4933	3.4685	THIN	1
C5h-acc-c2-sh-n3.tif	2.5142	0.8555	2.9389	FILO	1
C5h-acc-c2-sh-n3.tif	1.098	0.71	1.5465	MUSH	1
C5h-acc-c2-sh-n3.tif	0.7484	0.6247	1.1980	MUSH	1
C5h-acc-c2-sh-n3.tif	0.8243	0.3649	2.2590	THIN	1
C5h-acc-c2-sh-n3.tif	0.6392	0.5789	1.1042	THIN	1
C5h-acc-c2-sh-n3.tif	1.3016	0.5463	2.3826	THIN	1
					**7**
C6h-acc-c1-sh-n2.tif	1.2936	1.0928	1.1837	MUSH	1
C6h-acc-c1-sh-n2.tif	1.5191	1.0062	1.5097	MUSH	1
C6h-acc-c1-sh-n2.tif	1.2009	0.8743	1.3736	MUSH	1
C6h-acc-c1-sh-n2.tif	1.1896	1.1896	1.0000	MUSH	1
C6h-acc-c1-sh-n2.tif	1.0641	0.71	1.4987	MUSH	1
C6h-acc-c1-sh-n2.tif	1.0092	0.9969	1.0123	MUSH	1
					**6**
C6h-acc-c1-sh-n3.tif	1.6224	1.0041	1.6158	MUSH	1
C6h-acc-c1-sh-n3.tif	1.1364	0.8182	1.3889	MUSH	1
C6h-acc-c1-sh-n3.tif	1.5693	1.0947	1.4335	MUSH	1
C6h-acc-c1-sh-n3.tif	0.6331	0.8357	0.7576	MUSH	1
C6h-acc-c1-sh-n3.tif	0.7329	0.7115	1.0301	MUSH	1
					**5**
C6h-acc-c1-sh-n4.tif	1.3644	0.8194	1.6651	MUSH	1
C6h-acc-c1-sh-n4.tif	1.0909	0.7741	1.4092	MUSH	1
C6h-acc-c1-sh-n4.tif	1.1364	0.7727	1.4707	MUSH	1
C6h-acc-c1-sh-n4.tif	0.9632	1.2306	0.7827	MUSH	1
C6h-acc-c1-sh-n4.tif	0.8182	1.0454	0.7827	MUSH	1
C6h-acc-c1-sh-n4.tif	0.7524	0.8636	0.8712	MUSH	1
					**6**
C7h-acc-c2-sh-n4.tif	2.4018	1.223	1.9639	FILO	1
C7h-acc-c2-sh-n4.tif	1.3906	1.2129	1.1465	MUSH	1
C7h-acc-c2-sh-n4.tif	1.3422	1.5454	0.8685	MUSH	1
C7h-acc-c2-sh-n4.tif	1.3016	1.4374	0.9055	MUSH	1
C7h-acc-c2-sh-n4.tif	1.0365	1.2662	0.8186	MUSH	1
					**5**
C7h-acc-c2-sh-n5.tif	0.9204	0.8357	1.1014	MUSH	1

C7h-acc-c2-sh-n5.tif	0.8743	0.7938	1.1014	MUSH	1
C7h-acc-c2-sh-n5.tif	1.5673	1.3213	1.1862	MUSH	1
C7h-acc-c2-sh-n5.tif	1.3742	0.9728	1.4126	MUSH	1
C7h-acc-c2-sh-n5.tif	1.3368	1.065	1.2552	MUSH	1
C7h-acc-c2-sh-n5.tif	1.7727	1.0918	1.6236	MUSH	1
C7h-acc-c2-sh-n5.tif	0.9204	0.7187	1.2806	MUSH	1
C7h-acc-c2-sh-n5.tif				STUB	1
C7h-acc-c2-sh-n5.tif	0.9326	0.7399	1.2604	MUSH	1
					**9**
C7h-acc-c2-sh-n6.tif	1.3757	1.0454	1.3160	MUSH	1
C7h-acc-c2-sh-n6.tif	1.9984	0.8779	2.2763	MUSH	1
C7h-acc-c2-sh-n6.tif	1.0285	0.9326	1.1028	MUSH	1
C7h-acc-c2-sh-n6.tif	1.5654	0.9022	1.7351	MUSH	1
C7h-acc-c2-sh-n6.tif	0.9979	0.9969	1.0010	MUSH	1
C7h-acc-c2-sh-n6.tif	1.3422	0.8344	1.6086	MUSH	1
C7h-acc-c2-sh-n6.tif	1.5461	0.8344	1.8529	MUSH	1
					**7**
C7h-acc-c3-sh-n2abien.tif	1.0928	0.8093	1.3503	MUSH	1
C7h-acc-c3-sh-n2abien.tif	0.9458	0.8999	1.0510	MUSH	1
C7h-acc-c3-sh-n2abien.tif	1.0041	1.0385	0.9669	MUSH	1
C7h-acc-c3-sh-n2abien.tif	1.0365	0.9022	1.1489	MUSH	1
C7h-acc-c3-sh-n2abien.tif	1.4545	0.8232	1.7669	MUSH	1
C7h-acc-c3-sh-n2abien.tif	1	0.7727	1.2942	MUSH	1
C7h-acc-c3-sh-n2abien.tif	1.7487	0.8232	2.1243	MUSH	1
C7h-acc-c3-sh-n2abien.tif	0.8999	1.3275	0.6779	MUSH	1
					**8**
C6h-acc-c2-sh-n2.tif	1.0928	1.0365	1.0543	MUSH	1
C6h-acc-c2-sh-n2.tif	1.7279	1.065	1.6224	MUSH	1
C6h-acc-c2-sh-n2.tif	1.2281	1.2921	0.9505	MUSH	1
C6h-acc-c2-sh-n2.tif	1.5246	0.8965	1.7006	MUSH	1
C6h-acc-c2-sh-n2.tif	1.0947	0.646	1.6946	MUSH	1
C6h-acc-c2-sh-n2.tif	0.9204	0.6428	1.4319	MUSH	1
C6h-acc-c2-sh-n4.tif	2.5913	0.7767	3.3363	FILO	1
					**7**
C6h-acc-c2-sh-n4.tif	1.1373	0.8182	1.3900	MUSH	1

C6h-acc-c2-sh-n4.tif	1.5434	0.6711	2.2998	MUSH	1
C6h-acc-c2-sh-n4.tif	1.6527	0.6556	2.5209	MUSH	1
C6h-acc-c2-sh-n4.tif	1.4374	1.2	1.1978	MUSH	1
C6h-acc-c2-sh-n4.tif	2.3715	1.3252	1.7895	FILO	1
C6h-acc-c2-sh-n4.tif	1.5	0.7727	1.9412	MUSH	1
C6h-acc-c2-sh-n4.tif	1.8273	0.8194	2.2300	MUSH	1
					**7**
C7h-acc-c3-sh-n2bien.tif	0.7902	1.372	0.5759	MUSH	1
C7h-acc-c3-sh-n2bien.tif	0.8614	0.6371	1.3521	MUSH	1
C7h-acc-c3-sh-n2bien.tif	1.3323	0.7791	1.7101	MUSH	1
C7h-acc-c3-sh-n2bien.tif	0.9394	0.697	1.3478	MUSH	1
C7h-acc-c3-sh-n2bien.tif	1.1519	0.8243	1.3974	MUSH	1
C7h-acc-c3-sh-n2bien.tif	0.8359	0.904	0.9247	MUSH	1
C7h-acc-c3-sh-n2bien.tif	0.5821	0.5143	1.1318	THIN	1
					**7**

**Animal**	**CORE**						
**C2**	**FILO**	**MUSH**	**LONG THIN**	**THIN**	**STUBBY**	**BRANCHED**	**TOTAL**
N1	1	2	0	0	0	0	3
N3	1	7	0	0	0	0	8
N4	2	6	0	0	0	0	8
N5	0	5	0	0	0	0	5
N6A	2	3	0	0	0	0	5
**C2 average**	**1.2**	**4.6**	**0**	**0**	**0**	**0**	**5.8**
**C4**	**FILO**	**MUSH**	**LONG THIN**	**THIN**	**STUBBY**	**BRANCHED**	**TOTAL**
N2	3	3	0	0	0	0	6
**N3**	2	4	0	0	0	0	6
N5	0	5	0	0	0	0	5
**N7**	1	5	0	0	1	0	7
N8A	2	5	0	0	0	0	7
**C4 average**	**1.6**	**4.4**	**0**	**0**	**0.2**	**0**	**6.2**
**C5**	**FILO**	**MUSH**	**LONG THIN**	**THIN**	**STUBBY**	**BRANCHED**	**TOTAL**

N3	0	3	0	3	0	0	6
N4	0	5	0	1	0	0	6
N5	1	3	0	2	0	0	6
N6	0	3	0	4	0	0	7
N3 C2	1	2	0	4	0	0	7
**C5 average**	**0.4**	**3.2**	**0**	**2.8**	**0**	**0**	**6.4**

**C6**	**FILO**	**MUSH**	**LONG THIN**	**THIN**	**STUBBY**	**BRANCHED**	**TOTAL**
N2	0	6	0	0	0	0	6
N3	0	5	0	0	0	0	5
N4	0	6	0	0	0	0	6
N2C2	1	6	0	0	0	0	7
N4C2	1	6	0	0	0	0	7
**C6 average**	**0.40**	**5.80**	**0.00**	**0.00**	**0.00**	**0.00**	**5.67**

**C7**	**FILO**	**MUSH**	**LONG THIN**	**THIN**	**STUBBY**	**BRANCHED**	**TOTAL**
N4	2	6	0	0	0	0	8
N5	1	8	0	0	0	0	9
N6	0	7	0	0	0	0	7
N2	0	8	0	0	0	0	8
N2C3	0	6	0	1	0	0	7
**C7 average**	**0.6**	**7**	**0**	**0.2**	**0**	**0**	**7.8**

SHELL

**ANIMAL**	**FILO**	**MUSH**	**LONG THIN**	**THIN**	**STUBBY**	**BRANCHED**	**TOTAL**
**C2**	**1.2**	**4.6**	**0**	**0**	**0**	**0**	**5.8**
**C4**	**1.6**	**4.4**	**0**	**0**	**0.2**	**0**	**6.2**
**C5**	**0.4**	**3.2**	**0**	**2.8**	**0**	**0**	**6.4**
**C6**	**0.40**	**5.80**	**0**	**0**	**0**	**0**	**5.67**
**C7**	**0.6**	**7**	**0**	**0.2**	**0**	**0**	**7.8**

SHELL ANIMAL	FILO	MUSH	LONG THIN	THIN	STUBBY	BRANCHED	TOTAL
**C2**	20.69	79.31	0.00	0.00	0.00	0	100
**C4**	25.81	70.97	0.00	0.00	3.23	0	100
**C5**	6.25	50.00	0.00	43.75	0.00	0	100
**C6**	7.06	102.35	0.00	0.00	0.00	0	100
**C7**	7.69	89.74	0.00	2.56	0.00	0	100

Control
Females 48h
NAcc
CORE y SHELL
Dendritic length

CORE

	Animal	Length
1	C1-5.tif	88.197
2	C1-6.tif	68.537
3	C1-9.tif	54.6129
4	C1-10.tif	70.6309
	**C1**	**70.49**
10	C2-c1-40x1.tif	66.8527
11	C2-c1-40x2.tif	51.0931
12	C2-c2-10x3.tif	83.6567
13	C2-c2-10x3.tif	85.9026
14	C2-c2-10x3.tif	79.3958
	**C2**	**73.38**
19	C4-1-10x2.tif	56.2212
20	C4-1-10x2.tif	61.5439
21	C4-1-10x2.tif	44.825
22	C4-1-10x2.tif	90.5156
	**C4**	**63.28**
28	C5-2-10x45.tif	96.0819
29	C5-2-10x45.tif	143.451
30	C5-2-10x45.tif	151.3647
31	C5-2-10x45.tif	105.0567
32	C5-2-10x53.tif	113.1548
	**C5**	**121.82**
36	C6-2-10x76.tif	29.5598
37	C6-2-10x76.tif	57.3643
38	C6-2-10x76.tif	32.8843
39	C6-2-10x76.tif	28.4589
40	C6-2-10x76.tif	56.6274
	**C6-2-10x76.tif**	**40.98**
45	C7-1-10x110.tif	112.3085

SHELL

	Animal	Length
5	C1-100 sh.tif	110.2234
6	C1-100 sh.tif	75.6046
7	C1-100 sh2-2.tif	76.5047
8	C1-100x2.tif	67.6162
9	C1-100x6.tif	76.8997
	**C1**	**81.37**
15	C2-c4-10x7.tif	65.1995
16	C2-c6-10x13.tif	90.5259
17	C2-c6-10x13.tif	75.5957
18	C2-c6-10x13.tif	84.4524
	**C2**	**78.94**
23	C4-3-10x23.tif	97.7095
24	C4-3-10x23.tif	142.4557
25	C4-3-10x23.tif	104.1156
26	C4-3-10x23.tif	104.1156
27	C4-3-10x23.tif	140.3984
	**C4**	**117.76**
33	C5-2-10x53.tif	117.2803
34	C5-2-10x53.tif	124.3693
35	C5-2-10x53.tif	97.8592
41	C5-2-10x61.tif	137.0964
42	C5-2-10x61.tif	134.7163
43	C5-2-10x61.tif	77.375
44	C5-2-10x61.tif	120.2257
	**C5**	**115.56**
48	C7-3-10x137.tif	73.4041
49	C7-3-10x137.tif	58.6882
50	C7-3-10x137.tif	48.9741

46	C7-1-10x110.tif	93.8211		51	C7-3-10x137.tif	94.133
47	C7-1-10x110.tif	67.5233			**C7**	**68.80**
	**C7**	**91.22**				

**Average**		
**Animal**	**Core**	**Shell**
**C1**	**70.49**	**81.37**
**C2**	**73.38**	**78.94**
**C4**	**63.28**	**117.76**
**C5**	**121.82**	**115.56**
**C7**	**40.98**	**68.8**

Social exposure SE
Females 48h
NAcc
CORE
Spines density

	Animal	Length	Width	Ratio L/W	Subtype	Number
1	E1-c1-acc-co-n10a.tif	1.594	0.7727	2.06224926	MUSH	1
3	E1-c1-acc-co-n10a.tif	1.682	0.8182	2.05622097	MUSH	1
5	E1-c1-acc-co-n10a.tif	0.92	0.8381	1.09819831	MUSH	1
7	E1-c1-acc-co-n10a.tif	2.436	0.7329	3.32432801	FILO	1
9	E1-c1-acc-co-n10a.tif	1.162	1.1571	1.00380261	MUSH	1
11	E1-c1-acc-co-n10a.tif	2.616	0.8953	2.92226069	FILO	1
13	E1-c1-acc-co-n10a.tif	1.049	0.6555	1.60091533	MUSH	1
15	E1-c1-acc-co-n10a.tif	0.782	0.7399	1.05689958	MUSH	1
						**8**
17	E1-c2-acc-co-n2.tif	1.08	0.8826	1.22411058	MUSH	1
19	E1-c2-acc-co-n2.tif	0.782	0.7767	1.00682374	MUSH	1
21	E1-c2-acc-co-n2.tif	1.608	1.4889	1.08019343	MUSH	1
23	E1-c2-acc-co-n2.tif	1.793	0.7329	2.44644563	MUSH	1
25	E1-c2-acc-co-n2.tif	1.321	0.9091	1.45341547	MUSH	1
27	E1-c2-acc-co-n2.tif	0.936	0.9759	0.95911466	MUSH	1
29	E1-c2-acc-co-n2.tif	1.19	0.7741	1.53675236	MUSH	1
						**7**
31	E1-c2-acc-co-n5.tif	1.846	0.7056	2.61592971	MUSH	1
33	E1-c2-acc-co-n5.tif	1.773	0.8648	2.05053191	MUSH	1
35	E1-c2-acc-co-n5.tif	2.455	0.7187	3.4151941	FILO	1
37	E1-c2-acc-co-n5.tif	1.499	0.9371	1.59993597	MUSH	1
39	E1-c2-acc-co-n5.tif	4.184	0.5454	7.67143381	FILO	1
41	E1-c2-acc-co-n5.tif	1.423	0.9371	1.51840785	MUSH	1
43	E1-c2-acc-co-n5.tif	1.74	0.9728	1.78844572	MUSH	1
45	E1-c2-acc-co-n5.tif	1.676	0.6818	2.4586389	MUSH	1
47	E1-c2-acc-co-n5.tif	2.137	0.8182	2.61158641	FILO	1
	E1-c2-acc-co-n5.tif				BRANC	1
						**10**
49	E1-c2-acc-co-n5.tif	1.884	0.7455	2.52649229	MUSH	1
51	E1-c2-acc-co-n6.tif	1.248	0.8093	1.54219696	MUSH	1
53	E1-c2-acc-co-n6.tif	1.455	0.6508	2.23494161	MUSH	1
55	E1-c2-acc-co-n6.tif	0.858	0.9326	0.91957967	MUSH	1
57	E1-c2-acc-co-n6.tif	3.155	0.4312	7.31609462	FILO	1
59	E1-c2-acc-co-n6.tif	2.173	1.0494	2.07051649	FILO	1
61	E1-c2-acc-co-n6.tif	1.768	0.7741	2.28394264	MUSH	1
	E1-c2-acc-co-n6.tif				BRANC	1
63	E1-c2-acc-co-n6.tif	1.223	0.9664	1.26552152	MUSH	1
						**9**
65	E1-c2-acc-co-nnn.tif	1.789	0.8131	2.20022137	MUSH	1
67	E1-c2-acc-co-nnn.tif	1.248	0.7399	1.68684957	MUSH	1
69	E1-c2-acc-co-nnn.tif	2.101	0.5821	3.60968906	FILO	1
71	E1-c2-acc-co-nnn.tif	2.025	0.6938	2.9188527	FILO	1
73	E1-c2-acc-co-nnn.tif	1.095	0.6818	1.60560282	MUSH	1
75	E1-c2-acc-co-nnn.tif	1.481	0.7727	1.91613822	MUSH	1

77	E1-c2-acc-co-nnn.tif	1.21	0.6182	1.95778065	MUSH	1
79	E1-c2-acc-co-nnn.tif	2.318	0.6428	3.60563161	FILO	1
81	E1-c2-acc-co-nnn.tif	1.562	0.7847	1.99069708	MUSH	1
83	E1-c2-acc-co-nnn.tif	2.15	0.8381	2.5650877	FILO	1
85	E1-c2-acc-co-nnn.tif	1.684	1.14	1.47745614	MUSH	1
87	E1-c2-acc-co-nnn.tif	1.009	0.8965	1.1257111	MUSH	1
89	E1-c2-acc-co-nnn.tif	1.391	0.8093	1.71926356	MUSH	1
91	E1-c2-acc-co-nnn.tif	1.691	0.7187	2.35285933	MUSH	1
94	E1-c2-acc-co-nnn.tif	1.524	0.782	1.94872123	MUSH	1
96	E1-c2-acc-co-nnn.tif	1.519	0.7329	2.07272479	MUSH	1
						**16**
98	E2-c1-acc-co-n1a.tif	1.769	0.6098	2.90029518	MUSH	1
100	E2-c1-acc-co-n1a.tif	2.224	0.7399	3.00513583	FILO	1
102	E2-c1-acc-co-n1a.tif	1.765	0.6555	2.69275362	MUSH	1
104	E2-c1-acc-co-n1a.tif	1.196	0.7619	1.56936606	MUSH	1
106	E2-c1-acc-co-n1a.tif	1.548	0.7847	1.97285587	MUSH	1
108	E2-c1-acc-co-n1a.tif	0.846	0.7329	1.15363624	MUSH	1
110	E2-c1-acc-co-n1a.tif	1.511	1.0602	1.42520279	MUSH	1
112	E2-c1-acc-co-n1a.tif	0.836	0.9204	0.90797479	MUSH	1
114	E2-c1-acc-co-n1a.tif	1.351	0.71	1.90338028	MUSH	1
						**9**
116	E2-c1-acc-co-n3.tif	2.165	1.065	2.03305164	FILO	1
118	E2-c1-acc-co-n3.tif	1.455	0.9759	1.49113639	MUSH	1
120	E2-c1-acc-co-n3.tif	1.321	0.7071	1.8686183	MUSH	1
122	E2-c1-acc-co-n3.tif	1.673	1.3636	1.22660604	MUSH	1
124	E2-c1-acc-co-n3.tif	1.28	1.2936	0.9894867	MUSH	1
126	E2-c1-acc-co-n3.tif	0.909	1.1818	0.7692503	MUSH	1
128	E2-c1-acc-co-n3.tif	1.782	1.1291	1.57824816	MUSH	1
130	E2-c1-acc-co-n3.tif	2.273	0.8576	2.65006996	FILO	1
132	E2-c1-acc-co-n3.tif	1.427	0.7187	1.98594685	MUSH	1
						**9**
134	E2-c1-acc-co-n4 y n5.ti	1.183	1.4352	0.82406633	MUSH	1
136	E2-c1-acc-co-n4 y n5.ti	1.321	1.1931	1.10745118	MUSH	1
138	E2-c1-acc-co-n4 y n5.ti	1.139	0.7767	1.46658942	MUSH	1
140	E2-c1-acc-co-n4 y n5.ti	1.578	0.8684	1.81701981	MUSH	1
142	E2-c1-acc-co-n4 y n5.ti	3.259	0.6818	4.7801408	FILO	1
144	E2-c1-acc-co-n4 y n5.ti	2.182	1.3644	1.59945764	FILO	1
146	E2-c1-acc-co-n4 y n5.ti	1.391	1.1499	1.20932255	MUSH	1
149	E2-c1-acc-co-n4 y n5.ti	1.125	0.5473	2.05627627	MUSH	1
151	E2-c1-acc-co-n4 y n5.ti	1.123	0.7115	1.57779339	MUSH	1
153	E2-c1-acc-co-n4 y n5.ti	0.993	0.7496	1.3243063	MUSH	1
155	E2-c1-acc-co-n4 y n5.ti	0.591	0.8826	0.66949921	MUSH	1
						**11**
157	E2-c1-acc-co-n4 y n5.ti	1.385	0.9371	1.47839078	MUSH	1
159	E2-c1-acc-co-n4 y n5.ti	0.757	0.6428	1.17688239	MUSH	1
161	E2-c1-acc-co-n4 y n5.ti	0.902	0.71	1.27070423	MUSH	1
163	E2-c1-acc-co-n4 y n5.ti	1.099	0.8381	1.31177664	MUSH	1
165	E2-c1-acc-co-n4 y n5.ti	1.464	0.7951	1.84090052	MUSH	1

167	E2-c1-acc-co-n4 y n5.ti	1.201	0.7496	1.60205443	MUSH	1
169	E2-c1-acc-co-n4 y n5.ti	1.356	0.7329	1.8501842	MUSH	1
172	E2-c1-acc-co-n4 y n5.ti	1.009	0.7399	1.3639681	MUSH	1
174	E2-c1-acc-co-n4 y n5.ti	0.927	1.0909	0.84984875	MUSH	1
176	E2-c1-acc-co-n4 y n5.ti	1.545	0.8826	1.75096306	MUSH	1
						**10**
179	E2-c1-acc-co-n6.tif	1.248	0.9969	1.25198114	MUSH	1
181	E2-c1-acc-co-n6.tif	1.364	0.5909	2.30766627	MUSH	1
183	E2-c1-acc-co-n6.tif	1.543	0.7496	2.05896478	MUSH	1
185	E2-c1-acc-co-n6.tif	1.185	0.5183	2.28689948	MUSH	1
187	E2-c1-acc-co-n6.tif	1.185	0.6364	1.86250786	MUSH	1
189	E2-c1-acc-co-n6.tif	2.849	0.778	3.66208226	FILO	1
191	E2-c1-acc-co-n6.tif	1.374	0.8743	1.5717717	MUSH	1
193	E2-c1-acc-co-n6.tif	1.455	0.6818	2.13435025	MUSH	1
195	E2-c1-acc-co-n6.tif	0.777	0.9204	0.84387223	MUSH	1
						**9**
235	E2-c2-acc-co-n3.tif	0.777	0.5463	1.42174629	MUSH	1
237	E2-c2-acc-co-n3.tif	0.813	0.4635	1.75426106	MUSH	1
239	E2-c2-acc-co-n3.tif	0.904	0.8381	1.07863024	MUSH	1
241	E2-c2-acc-co-n3.tif	1.522	0.4791	3.17574619	MUSH	1
243	E2-c2-acc-co-n3.tif	0.879	0.8	1.0985	MUSH	1
245	E2-c2-acc-co-n3.tif	1.502	0.6247	2.4049944	MUSH	1
247	E2-c2-acc-co-n3.tif	0.824	0.4733	1.74160152	MUSH	1
249	E2-c2-acc-co-n3.tif	0.879	0.516	1.70310078	MUSH	1
251	E2-c2-acc-co-n3.tif	0.947	0.5821	1.62635286	MUSH	1
						**9**
279	E5-c1-acc-co-n1.tif	3.09	1.0816	2.85715607	FILO	1
281	E5-c1-acc-co-n1.tif	2.34	0.7879	2.97042772	FILO	1
283	E5-c1-acc-co-n1.tif	1.564	0.7373	2.12166011	MUSH	1
285	E5-c1-acc-co-n1.tif	1.222	0.7594	1.60850672	MUSH	1
287	E5-c1-acc-co-n1.tif	2.015	0.8441	2.38668404	FILO	1
289	E5-c1-acc-co-n1.tif	0.777	0.6392	1.21511264	MUSH	1
						**6**
291	E5-c1-acc-co-n2.tif	3.019	0.9413	3.20758525	FILO	1
293	E5-c1-acc-co-n2.tif	1.139	0.8788	1.29619936	MUSH	1
295	E5-c1-acc-co-n2.tif	1.279	0.8953	1.42834804	MUSH	1
297	E5-c1-acc-co-n2.tif	1.34	0.6673	2.00734302	MUSH	1
299	E5-c1-acc-co-n2.tif	1.952	1.2863	1.51721993	MUSH	1
301	E5-c1-acc-co-n2.tif	1.41	0.8835	1.59547255	MUSH	1
303	E5-c1-acc-co-n2.tif	1.825	0.7527	2.42394048	MUSH	1
						**7**
305	E5-c1-acc-co-n3.tif	2.054	0.9091	2.25882741	FILO	1
307	E5-c1-acc-co-n3.tif	1.499	0.8743	1.7148576	MUSH	1
309	E5-c1-acc-co-n3.tif	1.321	0.8965	1.47384272	MUSH	1
311	E5-c1-acc-co-n3.tif	1.642	0.9664	1.6990894	MUSH	1
313	E5-c1-acc-co-n3.tif	1.037	0.8295	1.24954792	MUSH	1
315	E5-c1-acc-co-n3.tif	1.775	0.8624	2.05820965	MUSH	1
317	E5-c1-acc-co-n3.tif	1.866	0.782	2.3859335	MUSH	1

319	E5-c1-acc-co-n3.tif	2.801	0.6115	4.579722	FILO	1
321	E5-c1-acc-co-n3.tif	1.614	1.0041	1.60750921	MUSH	1
324	E5-c1-acc-co-n3.tif	1.495	0.6923	2.15975733	MUSH	1
326	E5-c1-acc-co-n3.tif	2.199	0.7741	2.84045989	FILO	1
						**11**
329	E5-c1-acc-co-n5.tif	0.983	0.885	1.11107345	MUSH	1
331	E5-c1-acc-co-n5.tif	1.183	0.8187	1.44546232	MUSH	1
333	E5-c1-acc-co-n5.tif	1.556	0.8485	1.83394225	MUSH	1
335	E5-c1-acc-co-n5.tif	3.16	1.6994	1.85971519	FILO	1
337	E5-c1-acc-co-n5.tif	2.48	0.8069	3.07324328	FILO	1
339	E5-c1-acc-co-n5.tif	1.984	0.9782	2.02780617	MUSH	1
341	E5-c1-acc-co-n5.tif	1.605	0.76	2.11131579	MUSH	1
343	E5-c1-acc-co-n5.tif	1.37	0.7648	1.79171025	MUSH	1
345	E5-c1-acc-co-n5.tif	1.325	0.7666	1.72893295	MUSH	1
347	E5-c1-acc-co-n5.tif	0.949	0.7879	1.20395989	MUSH	1
						**10**
349	E6-c1-acc-co-n1a.tif	1.499	0.8648	1.73369565	MUSH	1
351	E6-c1-acc-co-n1a.tif	2.505	1.1589	2.1614462	FILO	1
353	E6-c1-acc-co-n1a.tif	0.849	1.1896	0.7138534	MUSH	1
355	E6-c1-acc-co-n1a.tif	2.189	0.7496	2.92075774	FILO	1
357	E6-c1-acc-co-n1a.tif	1.628	0.9969	1.63256094	MUSH	1
359	E6-c1-acc-co-n1a.tif	1.237	0.7951	1.5551503	MUSH	1
361	E6-c1-acc-co-n1a.tif	0.92	0.7455	1.23460765	MUSH	1
363	E6-c1-acc-co-n1a.tif	1.382	0.8455	1.63500887	MUSH	1
365	E6-c1-acc-co-n1a.tif	2.51	0.7329	3.42461454	FILO	1
367	E6-c1-acc-co-n1a.tif	0.873	0.8576	1.01807369	MUSH	1
369	E6-c1-acc-co-n1a.tif	1.427	0.7329	1.94651385	MUSH	1
371	E6-c1-acc-co-n1a.tif	0.591	0.8684	0.6804468	MUSH	1
373	E6-c1-acc-co-n1a.tif	0.638	0.9091	0.70179298	MUSH	1
375	E6-c1-acc-co-n1a.tif	0.633	0.7287	0.86880747	MUSH	1
						**14**
377	E6-c1-acc-co-n3.tif	1.556	0.4746	3.27876106	MUSH	1
379	E6-c1-acc-co-n3.tif	1.312	0.7056	1.85926871	MUSH	1
381	E6-c1-acc-co-n3.tif	1.972	0.5082	3.88016529	MUSH	1
383	E6-c1-acc-co-n3.tif	0.74	0.6508	1.13690842	MUSH	1
385	E6-c1-acc-co-n3.tif	1.364	0.8743	1.55964772	MUSH	1
387	E6-c1-acc-co-n3.tif	1.147	0.9204	1.2464146	MUSH	1
389	E6-c1-acc-co-n3.tif	0.973	0.6115	1.59084219	MUSH	1
391	E6-c1-acc-co-n3.tif	2.571	0.7399	3.47519935	FILO	1
393	E6-c1-acc-co-n3.tif	1.791	0.7565	2.36708526	MUSH	1
395	E6-c1-acc-co-n3.tif	1.749	0.7329	2.38668304	MUSH	1
397	E6-c1-acc-co-n3.tif	2.199	0.7187	3.05996939	FILO	1
399	E6-c1-acc-co-n3.tif	2.747	0.9556	2.87484303	FILO	1
401	E6-c1-acc-co-n3.tif	2.156	0.6953	3.10096361	FILO	1
403	E6-c1-acc-co-n3.tif	2.804	0.6923	4.04997833	FILO	1
405	E6-c1-acc-co-n3.tif	1.749	0.7455	2.34634474	MUSH	1
						**15**
407	E6-c2-acc-co-n1a.tif	1.444	0.5926	2.43638205	MUSH	1

409	E6-c2-acc-co-n1a.tif	0.836	0.8953	0.93343014	MUSH	1
411	E6-c2-acc-co-n1a.tif	1.1	1.4273	0.7708961	MUSH	1
413	E6-c2-acc-co-n1a.tif	1.495	0.5548	2.69502523	MUSH	1
415	E6-c2-acc-co-n1a.tif	2.136	0.9545	2.23813515	FILO	1
417	E6-c2-acc-co-n1a.tif	1.684	0.6182	2.72452281	MUSH	1
419	E6-c2-acc-co-n1a.tif	2.912	0.5	5.8246	FILO	1
421	E6-c2-acc-co-n1a.tif	1.708	0.5492	3.10997815	MUSH	1
423	E6-c2-acc-co-n1a.tif	1.578	0.5909	2.67033339	MUSH	1
425	E6-c2-acc-co-n1a.tif	1.129	0.45	2.50911111	MUSH	1
427	E6-c2-acc-co-n1a.tif	0.598	0.5926	1.00894364	MUSH	1
429	E6-c2-acc-co-n1a.tif	1.228	0.532	2.30845865	MUSH	1
						**12**
431	E6-c2-acc-co-n2.tif	1.049	0.9589	1.09437898	MUSH	1
433	E6-c2-acc-co-n2.tif	0.976	0.5821	1.67651606	MUSH	1
435	E6-c2-acc-co-n2.tif	0.683	0.7727	0.8843018	MUSH	1
437	E6-c2-acc-co-n2.tif	1.491	0.7714	1.93284936	MUSH	1
439	E6-c2-acc-co-n2.tif	0.818	0.5454	1.50018335	MUSH	1
441	E6-c2-acc-co-n2.tif	1.682	0.9147	1.83929157	MUSH	1
443	E6-c2-acc-co-n2.tif	2.713	0.6923	3.91853243	FILO	1
445	E6-c2-acc-co-n2.tif	1.611	0.8344	1.93060882	MUSH	1
447	E6-c2-acc-co-n2.tif	0.972	0.5473	1.77544308	MUSH	1
449	E6-c2-acc-co-n2.tif	0.638	0.6818	0.93575829	MUSH	1
451	E6-c2-acc-co-n2.tif	1.511	0.8731	1.73061505	MUSH	1
453	E6-c2-acc-co-n2.tif	1.525	0.8624	1.76785714	MUSH	1
						**12**
455	E6-c2-acc-co-n3.tif	2.586	0.8194	3.15560166	FILO	1
457	E6-c2-acc-co-n3.tif	1.185	0.7343	1.61419039	MUSH	1
459	E6-c2-acc-co-n3.tif	2.229	0.5909	3.77238111	FILO	1
461	E6-c2-acc-co-n3.tif	1.594	0.6364	2.50392835	MUSH	1
463	E6-c2-acc-co-n3.tif	1.08	0.6098	1.77172844	MUSH	1
465	E6-c2-acc-co-n3.tif	1.606	0.532	3.01954887	MUSH	1
467	E6-c2-acc-co-n3.tif	2.277	0.8295	2.74478602	FILO	1
469	E6-c2-acc-co-n3.tif	2.075	0.4545	4.56545655	FILO	1
471	E6-c2-acc-co-n3.tif	2.194	0.5082	4.31641086	FILO	1
473	E6-c2-acc-co-n3.tif	2.117	0.7187	2.94545708	FILO	1
475	E6-c2-acc-co-n3.tif	1.022	0.8576	1.19111474	MUSH	1
477	E6-c2-acc-co-n3.tif	1.673	0.7524	2.22301967	MUSH	1
479	E6-c2-acc-co-n3.tif	0.773	0.6833	1.13083565	MUSH	1
481	E6-c2-acc-co-n3.tif	0.643	0.7187	0.89439265	MUSH	1
483	E6-c2-acc-co-n3.tif	1.309	0.8492	1.5410975	MUSH	1
485	E6-c2-acc-co-n3.tif	1.241	0.7455	1.66425218	MUSH	1
487	E6-c2-acc-co-n3.tif	0.579	0.4832	1.19722682	MUSH	1
489	E6-c2-acc-co-n3.tif	1.18	0.7767	1.51937685	MUSH	1
						**18**
491	E6-c2-acc-co-n3b.tif	0.946	0.5143	1.83900447	MUSH	1
493	E6-c2-acc-co-n3b.tif	2.091	0.6818	3.06746847	FILO	1
495	E6-c2-acc-co-n3b.tif	1.77	1.3252	1.33594929	MUSH	1
497	E6-c2-acc-co-n3b.tif	1.286	0.8093	1.58952181	MUSH	1

499	E6-c2-acc-co-n3b.tif	3.049	0.7399	4.12015137	FILO	1
501	E6-c2-acc-co-n3b.tif	2.896	1.0842	2.67063272	FILO	1
503	E6-c2-acc-co-n3b.tif	2.293	0.8965	2.55783603	FILO	1
505	E6-c2-acc-co-n3b.tif	0.976	0.6818	1.43135817	MUSH	1
507	E6-c2-acc-co-n3b.tif	1.57	0.6953	2.25801812	MUSH	1
509	E6-c2-acc-co-n3b.tif	1.955	0.6923	2.82406471	MUSH	1
						**10**
511	E7-c1-acc-co-n1.tif	1.655	1.1364	1.45600141	MUSH	1
513	E7-c1-acc-co-n1.tif	1.45	1.1254	1.28807535	MUSH	1
515	E7-c1-acc-co-n1.tif	0.774	0.7565	1.02326504	MUSH	1
517	E7-c1-acc-co-n1.tif	1.151	0.7115	1.61742797	MUSH	1
519	E7-c1-acc-co-n1.tif	1.159	1.1896	0.97419301	MUSH	1
521	E7-c1-acc-co-n1.tif	2.058	0.782	2.63171355	FILO	1
523	E7-c1-acc-co-n1.tif	2.293	0.5821	3.9393575	FILO	1
525	E7-c1-acc-co-n1.tif	1.689	0.5492	3.07574654	MUSH	1
527	E7-c1-acc-co-n1.tif	1.637	0.7741	2.11471386	MUSH	1
529	E7-c1-acc-co-n1.tif	1.486	0.5695	2.609482	MUSH	1
531	E7-c1-acc-co-n1.tif	1.755	1.001	1.75284715	MUSH	1
533	E7-c1-acc-co-n1.tif	1.798	0.7727	2.32716449	MUSH	1
535	E7-c1-acc-co-n1.tif	1.248	0.8455	1.47616795	MUSH	1
537	E7-c1-acc-co-n1.tif	2.747	0.8093	3.39453849	FILO	1
539	E7-c1-acc-co-n1.tif	1.711	0.9664	1.77048841	MUSH	1
541	E7-c1-acc-co-n1.tif	1.733	1.0365	1.67158707	MUSH	1
543	E7-c1-acc-co-n1.tif	0.902	1.6363	0.55136589	MUSH	1
						**17**
545	E7-c1-acc-co-n3.tif	1.136	0.9545	1.19057098	MUSH	1
547	E7-c1-acc-co-n3.tif	0.864	0.7741	1.11561814	MUSH	1
549	E7-c1-acc-co-n3.tif	1.711	0.6711	2.54954552	MUSH	1
551	E7-c1-acc-co-n3.tif	2.791	0.8492	3.28603391	FILO	1
553	E7-c1-acc-co-n3.tif	2.569	0.7399	3.47195567	FILO	1
555	E7-c1-acc-co-n3.tif	2.521	0.6923	3.64206269	FILO	1
557	E7-c1-acc-co-n3.tif	1.4	0.6938	2.01830499	MUSH	1
559	E7-c1-acc-co-n3.tif	1.57	0.8455	1.85688941	MUSH	1
561	E7-c1-acc-co-n3.tif	2.864	0.7741	3.69978039	FILO	1
563	E7-c1-acc-co-n3.tif	0.864	1.0494	0.82294645	MUSH	1
565	E7-c1-acc-co-n3.tif	1.819	0.6711	2.71092237	MUSH	1
						**11**
567	E7-c1-acc-co-n4.tif	0.794	0.7329	1.08309456	MUSH	1
569	E7-c1-acc-co-n4.tif	1.506	0.8731	1.72431566	MUSH	1
571	E7-c1-acc-co-n4.tif	1.037	0.5301	1.95529145	MUSH	1
573	E7-c1-acc-co-n4.tif	0.612	0.6938	0.88137792	MUSH	1
575	E7-c1-acc-co-n4.tif	1.362	0.9022	1.50975393	MUSH	1
577	E7-c1-acc-co-n4.tif	1.224	0.7187	1.70293586	MUSH	1
579	E7-c1-acc-co-n4.tif	2.057	1.065	1.9314554	FILO	1
581	E7-c1-acc-co-n4.tif	1.367	0.6364	2.14739158	MUSH	1
583	E7-c1-acc-co-n4.tif	1.632	0.7847	2.07964827	MUSH	1
585	E7-c1-acc-co-n4.tif	2.436	1.0215	2.38511992	FILO	1
587	E7-c1-acc-co-n4.tif	0.712	0.7741	0.9191319	MUSH	1

589	E7-c1-acc-co-n4.tif	0.483	0.7187	0.67232503	MUSH	1
591	E7-c1-acc-co-n4.tif	1.21	0.8684	1.39371257	MUSH	1
593	E7-c1-acc-co-n4.tif	1.095	0.5183	2.11209724	MUSH	1
						**14**
595	E7-c1-acc-co-n6.tif	1.022	0.5454	1.87293729	MUSH	1
597	E7-c1-acc-co-n6.tif	1.022	0.5909	1.7287189	MUSH	1
599	E7-c1-acc-co-n6.tif	2.005	0.5301	3.78155065	FILO	1
601	E7-c1-acc-co-n6.tif	1.039	0.6953	1.49359988	MUSH	1
603	E7-c1-acc-co-n6.tif	1.778	1.3742	1.29377092	MUSH	1
605	E7-c1-acc-co-n6.tif	1.129	0.8042	1.40400398	MUSH	1
607	E7-c1-acc-co-n6.tif	1.038	0.6953	1.49216166	MUSH	1
609	E7-c1-acc-co-n6.tif	1.528	0.8042	1.90002487	MUSH	1
611	E7-c1-acc-co-n6.tif	1.049	0.5979	1.755143	MUSH	1
613	E7-c1-acc-co-n6.tif	0.795	0.532	1.49454887	MUSH	1
615	E7-c1-acc-co-n6.tif	1.248	0.646	1.93204334	MUSH	1
617	E7-c1-acc-co-n6.tif	2.58	1.0215	2.52540382	FILO	1
						**12**
619	E7-c2-acc-co-n8.tif	1.414	0.8403	1.68213733	MUSH	1
621	E7-c2-acc-co-n8.tif	1.139	0.6038	1.88655184	MUSH	1
623	E7-c2-acc-co-n8.tif	2.031	0.782	2.59654731	FILO	1
625	E7-c2-acc-co-n8.tif	1.243	0.7576	1.64044351	MUSH	1
627	E7-c2-acc-co-n8.tif	1.478	0.8006	1.84574069	MUSH	1
629	E7-c2-acc-co-n8.tif	1.744	0.8677	2.01014175	MUSH	1
631	E7-c2-acc-co-n8.tif	2.073	0.6428	3.22495333	FILO	1
634	E7-c2-acc-co-n8.tif	1.166	0.9091	1.28280717	MUSH	1
637	E7-c2-acc-co-n8.tif	2.332	0.8809	2.64672494	FILO	1
639	E7-c2-acc-co-n8.tif	0.951	0.7048	1.34931896	MUSH	1
641	E7-c2-acc-co-n8.tif	0.862	0.9116	0.94602896	MUSH	1
643	E7-c2-acc-co-n8.tif	1.841	0.9399	1.95839983	MUSH	1
645	E7-c2-acc-co-n8.tif	1.331	0.8809	1.51129527	MUSH	1
						**13**

**Animal**	**CORE**						
**E1**	**FILO**	**MUSH**	**LONG THIN**	**THIN**	**STUBBY**	**BRANCHED**	**TOTAL**
N10a	2	6	0	0	0	0	8
N2	0	7	0	0	0	0	7
N5	3	6	0	0	0	1	10
N6	2	6	0	0	0	1	9
Nnnn	4	12	0	0	0	0	16
**E1 average**	**2.2**	**7.4**	**0**	**0**	**0**	**0.4**	**10**
**E2**	**FILO**	**MUSH**	**LONG THIN**	**THIN**	**STUBBY**	**BRANCHED**	**TOTAL**
N1a	1	8	0	0	0	1	10
**N3**	2	7	0	0	0	0	9
N4	2	9	0	0	0	0	11
**N6**	1	8	0	0	0	1	10
N3C2	0	9	0	0	0	0	9

**N5**	0	10	0	0	0	0	10
**E2 average**	**1**	**8.5**	**0**	**0**	**0**	**0.33**	**9.83**

**E5**	**FILO**	**MUSH**	**LONG THIN**	**THIN**	**STUBBY**	**BRANCHED**	**TOTAL**
**N1**	3	3	0	0	0	0	6
**N2**	1	6	0	0	0	0	7
**N3**	3	8	0	0	0	0	11
**N5**	2	8	0	0	0	0	10
**N**							
**E5 average**	**2.25**	**6.25**	**0**	**0**	**0**	**0**	**8.5**

**E6**	**FILO**	**MUSH**	**LONG THIN**	**THIN**	**STUBBY**	**BRANCHED**	**TOTAL**
**N1a**	3	11	0	0	0	3	17
**N3**	5	10	0	0	0	0	15
**N1a C2**	2	10	0	0	0	0	12
**N2 C2**	1	11	0	0	0	0	12
**N3C2**	6	12	0	0	0	2	20
**N3b C2**	4	6	0	0	0	2	12
**E6 average**	**3.5**	**10**	**0**	**0**	**0**	**1.17**	**14.67**

**E7**	**FILO**	**MUSH**	**LONG THIN**	**THIN**	**STUBBY**	**BRANCHED**	**TOTAL**
**N1**	3	14	0	0	0	2	19
**N3**	4	7	0	0	0	0	11
**N4**	2	12	0	0	0	0	14
**N6**	2	10	0	0	0	0	12
**N8**	3	10	0	0	0	0	13
**E7 average**	**2.8**	**10.6**	**0**	**0**	**0**	**0.4**	**13.8**

**CORE ANIMAL**	**FILO**	**MUSH**	**LONG THIN**	**THIN**	**STUBBY**	**BRANCHED**	**TOTAL**
**E1**	**2.2**	**7.4**	**0**	**0**	**0**	**0.4**	**10**
**E2**	**1**	**8.5**	**0**	**0**	**0**	**0.33**	**9.83**
**E5**	**2.25**	**6.25**	**0**	**0**	**0**	**0**	**8.5**
**E6**	**3.5**	**10**	**0**	**0**	**0**	**1.17**	**14.67**
**E7**	**2.8**	**10.6**	**0**	**0**	**0**	**0.4**	**13.8**

Percentage

**CORE ANIMAL**	**FILO**	**MUSH**	**LONG THIN**	**THIN**	**STUBBY**	**BRANCHED**	**TOTAL**
**E1**	22.00	74.00	0.00	0.00	0.00	4.00	100
**E2**	10.17	86.44	0.00	0.00	0.00	3.39	100
**E5**	26.47	73.53	0.00	0.00	0.00	0.00	100
**E6**	23.86	68.18	0.00	0.00	0.00	7.95	100
**E7**	20.29	76.81	0.00	0.00	0.00	2.90	100

Social exposure SE
Females 48h
NAcc
SHELL
Spines density

Animal	Length	Width	Indice L/W	Subype	Number
E2-c1-acc-sh-n2.tif	0.5151	0.6061	0.84985976	MUSH	1
E2-c1-acc-sh-n2.tif	1.2731	0.8187	1.55502626	MUSH	1
E2-c1-acc-sh-n2.tif	0.681	0.697	0.97704448	MUSH	1
E2-c1-acc-sh-n2.tif	1.0976	0.7496	1.4642476	MUSH	1
E2-c1-acc-sh-n2.tif	1.4294	0.8555	1.67083577	MUSH	1
E2-c1-acc-sh-n2.tif	1.1212	0.8187	1.36948821	MUSH	1
E2-c1-acc-sh-n2.tif	1.723	0.6646	2.59253686	MUSH	1
E2-c1-acc-sh-n2.tif	0.8	0.5637	1.41919461	THIN	1
E2-c1-acc-sh-n2.tif	1.6186	1.0164	1.59248327	MUSH	1
E2-c1-acc-sh-n2.tif	2.3793	1.4786	1.60915731	FILO	1
					**10**
E2-c2-acc-.tif	1.3847	1.0255	1.35026816	MUSH	1
E2-c2-acc-.tif	2.07	1.0454	1.98010331	FILO	1
E2-c2-acc-.tif	1.7563	0.8743	2.00880705	MUSH	1
E2-c2-acc-.tif	0.9458	0.6331	1.49391881	MUSH	1
E2-c2-acc-.tif	2.058	1.0305	1.99708879	FILO	1
E2-c2-acc-.tif	1.6873	1.4552	1.15949698	MUSH	1
E2-c2-acc-.tif	2.4478	0.9022	2.71314564	FILO	1
E2-c2-acc-.tif	1.0611	0.8999	1.17913101	MUSH	1
E2-c2-acc-.tif	0.9728	0.9979	0.97484718	MUSH	1
					**9**
E2-c2-acc-sh-n1.tif	2.2985	1.7651	1.30219251	FILO	1
E2-c2-acc-sh-n1.tif	1.8323	1.065	1.72046948	MUSH	1
E2-c2-acc-sh-n1.tif	1.5481	1.3111	1.18076424	MUSH	1
E2-c2-acc-sh-n1.tif	0.8182	1.0947	0.74741938	MUSH	1
E2-c2-acc-sh-n1.tif	1.9037	1.1472	1.65943166	MUSH	1
E2-c2-acc-sh-n1.tif	2.2286	0.9545	2.33483499	FILO	1
					**6**
E2-c2-acc-sh-n1b.ti	1.0572	0.7727	1.36818947	MUSH	1
E2-c2-acc-sh-n1b.ti	1.6546	0.6428	2.57405103	MUSH	1
E2-c2-acc-sh-n1b.ti	3.3091	1.0255	3.22681619	FILO	1
E2-c2-acc-sh-n1b.ti	1.754	1.2197	1.43805854	MUSH	1
E2-c2-acc-sh-n1b.ti	1.3275	1.1226	1.18252272	MUSH	1
E2-c2-acc-sh-n1b.ti	2.5689	0.778	3.30192802	FILO	1
E2-c2-acc-sh-n1b.ti	1.0092	0.6364	1.5857951	MUSH	1
					**7**
E5-c1-acc-sh-n2.tif	1.7651	0.7343	2.40378592	MUSH	1
E5-c1-acc-sh-n2.tif	1.607	0.782	2.05498721	MUSH	1
E5-c1-acc-sh-n2.tif	0.9147	0.8042	1.13740363	MUSH	1
E5-c1-acc-sh-n2.tif	0.6833	0.7273	0.93950227	MUSH	1
E5-c1-acc-sh-n2.tif	1.5779	1.0464	1.50793196	MUSH	1
E5-c1-acc-sh-n2.tif	1.0255	0.9642	1.06357602	MUSH	1
E5-c1-acc-sh-n2.tif	1.5273	0.6315	2.41852732	MUSH	1
E5-c1-acc-sh-n2.tif	0.8381	0.6953	1.20537897	MUSH	1

E5-c1-acc-sh-n2.tif	0.7741	0.8731	0.88661093	MUSH	1
E5-c1-acc-sh-n2.tif	1.2239	0.8131	1.50522691	MUSH	1
E5-c1-acc-sh-n2.tif	1.0611	0.7187	1.47641575	MUSH	1
					**11**
E5-c1-acc-sh-n4.tif	1.607	0.6818	2.35699619	MUSH	1
E5-c1-acc-sh-n4.tif	1.1589	0.6315	1.83515439	MUSH	1
E5-c1-acc-sh-n4.tif	1.4841	0.6115	2.42698283	MUSH	1
E5-c1-acc-sh-n4.tif	0.7455	0.5548	1.34372747	THIN	1
E5-c1-acc-sh-n4.tif	0.7187	0.7071	1.01640503	MUSH	1
E5-c1-acc-sh-n4.tif	1.4785	0.8381	1.76410929	MUSH	1
E5-c1-acc-sh-n4.tif	1.528	0.5301	2.882475	LONG TH	1
					**7**
E5-c1-acc-sh-n8.tif	0.904	0.7329	1.23345613	MUSH	1
E5-c1-acc-sh-n8.tif	1.1822	1.1216	1.05402996	MUSH	1
E5-c1-acc-sh-n8.tif	0.8912	1.0018	0.88959872	MUSH	1
E5-c1-acc-sh-n8.tif	1.6319	0.963	1.69460021	MUSH	1
E5-c1-acc-sh-n8.tif	0.9413	0.7203	1.3068166	MUSH	1
E5-c1-acc-sh-n8.tif	1.7355	1.1658	1.4886773	MUSH	1
E5-c1-acc-sh-n8.tif	0.9116	0.8381	1.08769837	MUSH	1
E5-c1-acc-sh-n8.tif	1.2305	1.364	0.9021261	MUSH	1
E5-c1-acc-sh-n8.tif	1.8375	1.6675	1.10194903	MUSH	1
E5-c1-acc-sh-n8.tif	0.7398	0.6306	1.17316841	MUSH	1
					**10**
E6-c2-acc-sh-n1.tif	1.1589	1.0092	1.14833532	MUSH	1
E6-c2-acc-sh-n1.tif	1.5007	1.4003	1.07169892	MUSH	1
E6-c2-acc-sh-n1.tif	2.3461	0.6115	3.83663123	FILO	1
E6-c2-acc-sh-n1.tif	1.0728	0.6115	1.75437449	MUSH	1
E6-c2-acc-sh-n1.tif	1.3111	0.71	1.84661972	MUSH	1
E6-c2-acc-sh-n1.tif	2.0894	0.5821	3.58941763	FILO	1
E6-c2-acc-sh-n1.tif	0.5622	0.5979	0.94029102	STUB	1
E6-c2-acc-sh-n1.tif	2.0297	0.9371	2.16593747	FILO	1
E6-c2-acc-sh-n1.tif	1.1615	0.7329	1.58480011	MUSH	1
E6-c2-acc-sh-n1.tif	0.5082	0.4091	1.24223906	THIN	1
E6-c2-acc-sh-n1.tif	1.3368	0.8576	1.55876866	MUSH	1
E6-c2-acc-sh-n1.tif	1.4273	1.2936	1.10335498	MUSH	1
E6-c2-acc-sh-n1.tif	1.1651	0.646	1.80356037	MUSH	1
E6-c2-acc-sh-n1.tif	1.3368	0.8455	1.58107629	MUSH	1
E6-c2-acc-sh-n1.tif	0.5021	0.5622	0.89309854	STUB	1
E6-c2-acc-sh-n1.tif	0.6953	0.3884	1.79016478	THIN	1
					**16**
E6-c2-acc-sh-n2.tif	0.646	0.854	0.75644028	MUSH	1
E6-c2-acc-sh-n2.tif	0.6711	0.6064	1.10669525	MUSH	1
E6-c2-acc-sh-n2.tif	0.7496	0.7727	0.97010483	MUSH	1
E6-c2-acc-sh-n2.tif	2.7246	0.7524	3.62121212	FILO	1
E6-c2-acc-sh-n2.tif	1.2077	0.6953	1.73694808	MUSH	1
E6-c2-acc-sh-n2.tif	1.8284	0.6428	2.84443062	MUSH	1
E6-c2-acc-sh-n2.tif	2.0328	1.0255	1.98225256	FILO	1
E6-c2-acc-sh-n2.tif	2.2782	0.7455	3.05593561	FILO	1

E6-c2-acc-sh-n2.tif	0.7329	0.6182	1.18553866	MUSH	1
					**9**
E6-c2-acc-sh-n2a.tif	1.2279	0.7184	1.70921492	MUSH	1
E6-c2-acc-sh-n2a.tif	1.65	0.6364	2.59270899	MUSH	1
E6-c2-acc-sh-n2a.tif	1.2556	0.7861	1.59725226	MUSH	1
E6-c2-acc-sh-n2a.tif	1.3982	0.8809	1.58724032	MUSH	1
E6-c2-acc-sh-n2a.tif	0.7714	0.5301	1.45519713	THIN	1
E6-c2-acc-sh-n2a.tif	1.2335	0.5043	2.4459647	LONG TH	1
E6-c2-acc-sh-n2a.tif	1.8247	0.6442	2.83250543	MUSH	1
E6-c2-acc-sh-n2a.tif	0.8006	0.8381	0.95525594	MUSH	1
E6-c2-acc-sh-n2a.tif	1.188	0.7132	1.66573191	MUSH	1
E6-c2-acc-sh-n2a.tif	1.4824	0.5821	2.54664147	LONG TH	1
E6-c2-acc-sh-n2a.tif	1.7591	0.5213	3.37444849	LONG TH	1
					**11**
E6-c2-acc-sh-n3.tif	2.073	0.936	2.21474359	FILO	1
E6-c2-acc-sh-n3.tif	0.7285	1.0073	0.72322049	MUSH	1
E6-c2-acc-sh-n3.tif	0.7496	0.7672	0.97705944	MUSH	1
E6-c2-acc-sh-n3.tif	2.5299	0.691	3.66121563	FILO	1
E6-c2-acc-sh-n3.tif	2.2027	0.3636	6.0580308	FILO	1
E6-c2-acc-sh-n3.tif	1.6666	0.9394	1.77411113	MUSH	1
E6-c2-acc-sh-n3.tif	2.7274	0.7879	3.4616068	FILO	1
E6-c2-acc-sh-n3.tif	1.6083	0.849	1.89434629	MUSH	1
E6-c2-acc-sh-n3.tif	1.2215	0.849	1.43875147	MUSH	1
E6-c2-acc-sh-n3.tif	0.7203	0.7666	0.93960344	MUSH	1
E6-c2-acc-sh-n3.tif	2.3803	0.5563	4.2788064	FILO	1
E6-c2-acc-sh-n3.tif	0.904	0.5151	1.75499903	THIN	1
E6-c2-acc-sh-n3.tif	0.901	0.421	2.14014252	THIN	1
					**13**
E6-c2-acc-sh-n4.tif	1.9545	0.6757	2.89255587	MUSH	1
E6-c2-acc-sh-n4.tif	1.2856	0.8455	1.5205204	MUSH	1
E6-c2-acc-sh-n4.tif	1.0842	0.5909	1.83482823	LONG TH	1
E6-c2-acc-sh-n4.tif	1.3213	0.646	2.04535604	MUSH	1
E6-c2-acc-sh-n4.tif	0.6757	0.6428	1.05118233	MUSH	1
E6-c2-acc-sh-n4.tif	1.7308	0.8779	1.97152295	MUSH	1
E6-c2-acc-sh-n4.tif	2.0123	0.9271	2.17053177	FILO	1
E6-c2-acc-sh-n4.tif	0.9969	0.6953	1.4337696	MUSH	1
E6-c2-acc-sh-n4.tif	1.065	0.5473	1.94591632	LONG TH	1
					**9**
E7-c1-acc-sh-n2.tif	1.103	0.7279	1.51531804	MUSH	1
E7-c1-acc-sh-n2.tif	0.624	0.575	1.08521739	THIN	1
E7-c1-acc-sh-n2.tif	1.4699	0.9716	1.51286538	MUSH	1
E7-c1-acc-sh-n2.tif	1.385	1.0018	1.38251148	MUSH	1
E7-c1-acc-sh-n2.tif	2.3394	0.5463	4.28226249	FILO	1
E7-c1-acc-sh-n2.tif	2.4259	0.8441	2.87394858	FILO	1
E7-c1-acc-sh-n2.tif	4.2	0.6653	6.31294153	FILO	1
E7-c1-acc-sh-n2.tif	1.2494	0.9438	1.32379741	MUSH	1
E7-c1-acc-sh-n2.tif	1.2215	1.0303	1.18557702	MUSH	1
E7-c1-acc-sh-n2.tif	0.5231	0.963	0.54319834	MUSH	1

E7-c1-acc-sh-n2.tif	0.7971	0.5758	1.38433484	THIN	1
					**11**
E7-c1-acc-sh-n4.tif	0.5571	0.5693	0.97857017	STUB	1
E7-c1-acc-sh-n4.tif	1.0449	0.4545	2.2990099	LONG TH	1
E7-c1-acc-sh-n4.tif	0.901	0.4626	1.94768699	THIN	1
E7-c1-acc-sh-n4.tif	0.7576	0.5884	1.28755948	THIN	1
E7-c1-acc-sh-n4.tif	0.7273	0.6673	1.08991458	MUSH	1
E7-c1-acc-sh-n4.tif	1.2007	0.6442	1.86386215	MUSH	1
E7-c1-acc-sh-n4.tif	1.5992	0.6061	2.6385085	MUSH	1
E7-c1-acc-sh-n4.tif	1.9244	0.575	3.34678261	LONG TH	1
E7-c1-acc-sh-n4.tif	1.5323	0.7298	2.09961633	MUSH	1
E7-c1-acc-sh-n4.tif	1.0584	0.5852	1.80861244	LONG TH	1
E7-c1-acc-sh-n4.tif	0.7094	0.5178	1.37002704	THIN	1
E7-c1-acc-sh-n4.tif	2.1683	0.6371	3.40339036	FILO	1
E7-c1-acc-sh-n4.tif	1.5454	0.6485	2.38303778	MUSH	1
E7-c1-acc-sh-n4.tif	1.3156	0.5231	2.51500669	LONG TH	1
					**14**
E7-c1-acc-sh-n5.tif	1.2369	0.9668	1.27937526	MUSH	1
E7-c1-acc-sh-n5.tif	1.6186	1.0748	1.5059546	MUSH	1
E7-c1-acc-sh-n5.tif	1.9273	0.9251	2.08334234	MUSH	1
E7-c1-acc-sh-n5.tif	0.9236	0.5789	1.59543963	THIN	1
E7-c1-acc-sh-n5.tif	0.7152	0.7453	0.95961358	MUSH	1
E7-c1-acc-sh-n5.tif	1.238	0.681	1.81791483	MUSH	1
E7-c1-acc-sh-n5.tif	0.691	0.5588	1.23657838	THIN	1
E7-c1-acc-sh-n5.tif	0.9866	0.7184	1.37332962	MUSH	1
E7-c1-acc-sh-n5.tif	0.7931	0.6857	1.15662826	MUSH	1
E7-c1-acc-sh-n5.tif	0.6478	0.6392	1.01345432	MUSH	1
					**10**
E7-c1-acc-sh-n6.tif	1.6642	0.7203	2.31042621	MUSH	1
E7-c1-acc-sh-n6.tif	0.7453	0.4616	1.61460139	THIN	1
E7-c1-acc-sh-n6.tif	2.2952	0.8741	2.62578652	FILO	1
E7-c1-acc-sh-n6.tif	1.4126	1.1834	1.19367923	MUSH	1
E7-c1-acc-sh-n6.tif	2.1894	1.2365	1.77064294	FILO	1
E7-c1-acc-sh-n6.tif	1.2215	0.7576	1.61232841	MUSH	1
E7-c1-acc-sh-n6.tif	1.5514	1.0073	1.54015685	MUSH	1
E7-c1-acc-sh-n6.tif	1.0356	1.2571	0.82380081	MUSH	1
E7-c1-acc-sh-n6.tif	1.89	1.2155	1.55491567	MUSH	1
E7-c1-acc-sh-n6.tif	0.8999	0.5463	1.64726341	THIN	1
E7-c1-acc-sh-n6.tif	2.2352	0.7931	2.81830791	FILO	1
					**11**
E7-c1-acc-sh-n8.tif	1.6186	1.0073	1.60686985	MUSH	1
E7-c1-acc-sh-n8.tif	1.6542	1.0841	1.525874	MUSH	1
E7-c1-acc-sh-n8.tif	1.0748	0.7576	1.4186906	MUSH	1
E7-c1-acc-sh-n8.tif	2.023	0.8683	2.32983992	FILO	1
E7-c1-acc-sh-n8.tif	1.6763	0.9394	1.78443687	MUSH	1
E7-c1-acc-sh-n8.tif	1.6478	0.8741	1.885139	MUSH	1
E7-c1-acc-sh-n8.tif	1.7654	0.6428	2.7464219	MUSH	1
E7-c1-acc-sh-n8.tif	1.1658	0.6728	1.73275862	MUSH	1

E7-c1-acc-sh-n8.tif	1.1097	0.6218	1.78465745	MUSH	1
E7-c1-acc-sh-n8.tif	2.0946	0.9111	2.29897926	FILO	1
E7-c1-acc-sh-n8.tif	2.5371	1.1598	2.18753233	FILO	1
E7-c1-acc-sh-n8.tif	0.8131	0.624	1.30304487	MUSH	1
					**12**
E2-c2-acc-co-n6.tif	1.1933	0.6933	1.72118852	MUSH	1
E2-c2-acc-co-n6.tif	0.6684	0.6209	1.07650185	MUSH	1
E2-c2-acc-co-n6.tif	1.0859	0.833	1.30360144	MUSH	1
E2-c2-acc-co-n6.tif	1.4005	0.7678	1.82404272	MUSH	1
E2-c2-acc-co-n6.tif	1.7149	0.8095	2.11846819	MUSH	1
E2-c2-acc-co-n6.tif	0.6615	0.8781	0.75333106	MUSH	1
E2-c2-acc-co-n6.tif	1.0701	0.6023	1.77668936	MUSH	1
E2-c2-acc-co-n6.tif	0.9817	0.7529	1.30389162	MUSH	1
E2-c2-acc-co-n6.tif	1.1359	0.7693	1.47653711	MUSH	1
E2-c2-acc-co-n6.tif	0.9488	0.8896	1.06654676	MUSH	1
E2-c2-acc-co-n6.tif	1.0476	1.1208	0.93468951	MUSH	1
E2-c2-acc-co-n6.tif	0.619	0.7982	0.77549486	MUSH	1
E2-c2-acc-co-n6.tif				BRANC	2
					**14**
E5-c1-acc-sh-n3.tif	1.8579	0.7841	2.36946818	MUSH	1
E5-c1-acc-sh-n3.tif	0.8548	0.7937	1.07698123	MUSH	1
E5-c1-acc-sh-n3.tif	2.1776	0.7197	3.02570515	FILO	1
E5-c1-acc-sh-n3.tif	1.7916	0.7937	2.25727605	MUSH	1
E5-c1-acc-sh-n3.tif	1.1586	0.8912	1.30004488	MUSH	1
E5-c1-acc-sh-n3.tif	1.0471	0.6629	1.5795746	MUSH	1
E5-c1-acc-sh-n3.tif	1.0495	0.9294	1.12922315	MUSH	1
E5-c1-acc-sh-n3.tif	0.6962	0.6065	1.14789777	MUSH	1
E5-c1-acc-sh-n3.tif	1.5886	0.6667	2.38278086	MUSH	1
E5-c1-acc-sh-n3.tif	1.3204	0.7027	1.879038	MUSH	1
E5-c1-acc-sh-n3.tif	2.3194	0.9096	2.54991205	FILO	1
E5-c1-acc-sh-n3.tif				BRANC	2
					**13**
E5-c1-acc-sh-n1.tif	1.0859	1.175	0.92417021	MUSH	1
E5-c1-acc-sh-n1.tif	0.9923	0.9923	1	MUSH	1
E5-c1-acc-sh-n1.tif	2.6418	0.7404	3.56807131	FILO	1
E5-c1-acc-sh-n1.tif	1.3487	0.9096	1.48273967	MUSH	1
E5-c1-acc-sh-n1.tif	1.5771	0.8278	1.90517033	MUSH	1
E5-c1-acc-sh-n1.tif	0.7962	1.0812	0.736404	MUSH	1
E5-c1-acc-sh-n1.tif	1.4758	0.6065	2.43330585	MUSH	1
E5-c1-acc-sh-n1.tif	2.5979	0.8758	2.96631651	FILO	1
E5-c1-acc-sh-n1.tif	4.5214	0.9024	5.01041667	FILO	1
				BRANC	1
					**10**

	**Animal**	**CORE**						
**E2**		**FILO**	**MUSH**	**LONG THIN**	**THIN**	**STUBBY**	**BRANCHI**	**TOTAL**
N2		1	8	0	1	0	0	10
Nacc		3	6	0	0	0	1	10

N1	2	4	0	0	0	0	6
N1b	2	5	0	0	0	0	7
N6	0	12	0	0	0	2	14
**E2 average**	**1.6**	**7**	**0**	**0.2**	**0**	**0.6**	**9.4**

**E5**	**FILO**	**MUSH**	**LONG THIN**	**THIN**	**STUBBY**	**BRANCHI**	**TOTAL**
N2	0	11	0	0	0	0	11
**N4**	0	5	1	1	0	0	7
N8	0	10	0	0	0	0	10
**N3**	2	9	0	0	0	2	13
N	3	6	0	0	0	1	10
**E5 average**	**1.00**	**8.20**	**0.20**	**0.20**	**0.00**	**0.60**	**9.33**

**E6**	**FILO**	**MUSH**	**LONG THIN**	**THIN**	**STUBBY**	**BRANCHI**	**TOTAL**
N1	3	9	0	2	2	0	16
**N2**	3	6	0	0	0	0	9
N2A	0	7	3	1	0	0	11
**N3 C2**	5	6	0	2	0	1	14
N4 C2	1	6	2	0	0	1	10
**E6 average**	**2.4**	**6.8**	**1**	**1**	**0.4**	**0.4**	**12**

**E7**	**FILO**	**MUSH**	**LONG THIN**	**THIN**	**STUBBY**	**BRANCHI**	**TOTAL**
N2	3	6	0	2	0	1	12
N4	1	5	4	3	1	0	14
N5	0	8	0	2	0	0	10
N6	3	6	0	2	0	0	11
N8	3	9	0	0	0	0	12
**E7 average**	**2**	**6.8**	**0.8**	**1.8**	**0.2**	**0.2**	**11.8**

SHELL

**ANIMAL**	**FILO**	**MUSH**	**LONG THIN**	**THIN**	**STUBBY**	**BRANCHI**	**TOTAL**
**E2**	**1.6**	**7**	**0**	**0.2**	**0**	**0.6**	**9.4**
**E5**	**1.00**	**8.20**	**0.20**	**0.20**	**0.00**	**0.60**	**9.33**
**E6**	**2.4**	**6.8**	**1**	**1**	**0.4**	**0.4**	**12**
**E7**	**2**	**6.8**	**0.8**	**1.8**	**0.2**	**0.2**	**11.8**

Percentage
SHELL

**ANIMAL**	**FILO**	**MUSH**	**LONG THIN**	**THIN**	**STUBBY**	**BRANCHI**	**TOTAL**
**E2**	17.02	74.47	0.00	2.13	0.00	6.38	100
**E5**	10.71	87.86	2.14	2.14	0.00	6.43	100
**E6**	20.00	56.67	8.33	8.33	3.33	3.33	100
**E7**	16.95	57.63	6.78	15.25	1.69	1.69	100

Social exposure SE
Females 48h
NAcc
CORE
Dendritic length

	ANIMAL	Length
1	E1-c1-core.tif	101.3879
2	E1-c1-core.tif	98.6142
3	E1-c1-core.tif	97.1567
	**E1**	**99.053**
7	E2-3.tif	85.9018
8	E2-3.tif	100.4063
9	E2-3.tif	159.4937
10	E2-3.tif	98.9262
	**E2**	**111.18**
15	E4-c2-co.tif	98.7421
16	E4-c2-co.tif	138.0153
17	E4-c2-co.tif	78.4934
18	E4-c2-co.tif	84.8407
19	E4-c2-co.tif	93.0329
	**E4**	**98.62**
23	E5-c1-co004.t	141.9772
24	E5-c1-co004.t	154.0812
25	E5-c1-co004.t	98.6136
	**E5**	**131.56**
31	E6-c1-co002.t	152.2095
32	E6-c1-co002.t	241.8291
33	E6-c1-co005.t	177.3155
34	E6-c1-co005.t	181.9086
	**E6**	**188.32**
39	E7h-c1-co.tif	153.8729
40	E7h-c1-co.tif	155.5796
41	E7h-c1-co.tif	107.5634
42	E7h-c1-co.tif	149.2949
	**E7**	**141.58**

	ANIMAL	Length
4	E1-c1-sh3.tif	139.8297
5	E1-c1-sh3.tif	119.0283
6	E1-c1-sh3.tif	89.8492
	**E1**	**116.24**
11	E2-8.tif	141.7105
12	E2-8.tif	111.6923
13	E2-8.tif	128.5661
14	E2-8.tif	125.4419
	**E2**	**126.85**
20	E4-c3-sh.tif	172.0588
21	E4-c3-sh.tif	131.9322

22	E4-c3-sh.tif	201.0809
	**E4**	**168.36**
26	E5-c1-sh003.t	120.101
27	E5-c1-sh003.t	96.2104
28	E5-c1-sh004.t	190.3444
29	E5-c1-sh004.t	122.4341
30	E5-c1-sh004.t	143.4164
	**E5**	**134.50**
35	E6-c2-sh002.t	74.0972
36	E6-c2-sh007.t	240.9838
37	E6-c2-sh007.t	213.8731
38	E6-c2-sh007.t	224.4426
	**E6**	**188.35**
43	E7h-c3-sh003.	129.5456
44	E7h-c3-sh003.	155.4435
45	E7h-c3-sh003.	116.5377
	E7	133.84

Average

**ANIMAL**	**CORE**	**SHELL**
**E1**	**99.05**	**116.24**
**E2**	**111.18**	**126.85**
**E4**	**98.62**	**168.36**
**E5**	**131.56**	**134.5**
**E6**	**188.32**	**188.35**
**E7**	**141.58**	**133.84**

SCM
Females 48h
NAcc
CORE
Spines subtypes

	Animal	Length	Width	Indice L/W	Subtype	Number
139	V2-c1-acc-co-n1.tif	1.0947	0.8826	1.240	MUSH	1
141	V2-c1-acc-co-n1.tif	1.6141	1.001	1.612	MUSH	1
143	V2-c1-acc-co-n1.tif	1.5514	1.0842	1.431	MUSH	1
145	V2-c1-acc-co-n1.tif	2.1252	1.2539	1.695	FILO	1
147	V2-c1-acc-co-n1.tif	2.0914	1.3182	1.587	FILO	1
149	V2-c1-acc-co-n1.tif	1.801	0.8381	2.149	MUSH	1
151	V2-c1-acc-co-n1.tif	1.1097	0.9204	1.206	MUSH	1
153	V2-c1-acc-co-n1.tif	1.5027	1.6818	0.894	MUSH	1
155	V2-c1-acc-co-n1.tif	2.1609	1.0918	1.979	FILO	1
157	V2-c1-acc-co-n1.tif	1.7779	0.8194	2.170	MUSH	1
159	V2-c1-acc-co-n1.tif	1.8835	0.7343	2.565	MUSH	1
161	V2-c1-acc-co-n1.tif	0.9458	0.9371	1.009	MUSH	1
	V2-c1-acc-co-n1.tif	3.5214	0.8357	4.214	FILO	1
						**13**
373	V2-c2-acc-co-n1a.tif	1.0114	0.7484	1.351	MUSH	1
375	V2-c2-acc-co-n1a.tif	1.7641	0.8809	2.003	MUSH	1
378	V2-c2-acc-co-n1a.tif	1.4852	0.763	1.947	MUSH	1
380	V2-c2-acc-co-n1a.tif	1.1359	0.6	1.893	LONG THIN	1
382	V2-c2-acc-co-n1a.tif	1.5529	1.3992	1.110	MUSH	1
384	V2-c2-acc-co-n1a.tif	1.0519	0.8154	1.290	MUSH	1
386	V2-c2-acc-co-n1a.tif	1.3423	0.681	1.971	MUSH	1
						**7**
163	V2-c1-acc-co-n3				BRANC	2
275	V2-c1-acc-co-n3.tif	1.9118	1.3	1.471	MUSH	1
277	V2-c1-acc-co-n3.tif	1.2239	0.646	1.895	MUSH	1
279	V2-c1-acc-co-n3.tif	4.0268	0.9791	4.113	FILO	1
281	V2-c1-acc-co-n3.tif	1.3213	1.511	0.874	MUSH	1
283	V2-c1-acc-co-n3.tif	2.1988	1.3369	1.645	FILO	1
285	V2-c1-acc-co-n3.tif	1.8636	1.2035	1.548	MUSH	1
287	V2-c1-acc-co-n3.tif	3.0086	0.8344	3.606	FILO	1
289	V2-c1-acc-co-n3.tif	2.623	1.0994	2.386	FILO	1
291	V2-c1-acc-co-n3.tif	1.5779	0.7847	2.011	MUSH	1
293	V2-c1-acc-co-n3.tif	1.4266	0.5082	2.807	LONG THIN	1
295	V2-c1-acc-co-n3.tif	1.7651	1.2281	1.437	MUSH	1
297	V2-c1-acc-co-n3.tif	0.8344	1.2239	0.682	MUSH	1
						**14**
299	V2-c1-acc-co-n4.tif	1.6769	1.4439	1.161	MUSH	1
301	V2-c1-acc-co-n4.tif	0.9204	1.1391	0.808	MUSH	1
303	V2-c1-acc-co-n4.tif	0.9193	0.9371	0.981	MUSH	1
305	V2-c1-acc-co-n4.tif	3.6692	1.356	2.706	FILO	1
307	V2-c1-acc-co-n4.tif	1.3492	1.0464	1.289	MUSH	1
309	V2-c1-acc-co-n4.tif	2.623	1.0572	2.481	FILO	1

						**6**
311	V2-c1-acc-co-n5.tif	2.3461	1.3877	1.691	FILO	1
313	V2-c1-acc-co-n5.tif	2.7638	0.8131	3.399	FILO	1
315	V2-c1-acc-co-n5.tif	1.9735	0.9969	1.980	MUSH	1
317	V2-c1-acc-co-n5.tif	1.0804	1.1364	0.951	MUSH	1
319	V2-c1-acc-co-n5.tif	2.6084	1.1897	2.192	FILO	1
321	V2-c1-acc-co-n5.tif	2.1728	0.7938	2.737	FILO	1
						**6**
323	V2-c1-acc-co-n6.tif	1.7297	0.7273	2.378	MUSH	1
325	V2-c1-acc-co-n6.tif	1.1515	0.7885	1.460	MUSH	1
327	V2-c1-acc-co-n6.tif	2.6612	1.1113	2.395	FILO	1
329	V2-c1-acc-co-n6.tif	3.2708	0.9116	3.588	FILO	1
331	V2-c1-acc-co-n6.tif	3.8486	0.8271	4.653	FILO	1
333	V2-c1-acc-co-n6.tif	0.9583	1.4358	0.667	MUSH	1
335	V2-c1-acc-co-n6.tif	1.3212	1.0343	1.277	MUSH	1
337	V2-c1-acc-co-n6.tif	1.7102	0.9758	1.753	MUSH	1
339	V2-c1-acc-co-n6.tif	1.6336	0.9511	1.718	MUSH	1
341	V2-c1-acc-co-n6.tif	0.6923	0.7648	0.905	MUSH	1
343	V2-c1-acc-co-n6.tif	1.106	0.7576	1.460	MUSH	1
345	V2-c1-acc-co-n6.tif	0.8403	0.8539	0.984	MUSH	1
						**12**
388	V3-c1-acc-co-n3a.tif	1.2348	0.8232	1.500	MUSH	1
390	V3-c1-acc-co-n3a.tif	1.0947	0.8743	1.252	MUSH	1
392	V3-c1-acc-co-n3a.tif	0.9556	0.6757	1.414	MUSH	1
394	V3-c1-acc-co-n3a.tif	1.1651	0.8455	1.378	MUSH	1
396	V3-c1-acc-co-n3a.tif	1.2	0.8131	1.476	MUSH	1
398	V3-c1-acc-co-n3a.tif	1.8741	1.2921	1.450	MUSH	1
400	V3-c1-acc-co-n3a.tif	0.6818	0.8455	0.806	MUSH	1
402	V3-c1-acc-co-n3a.tif	1.0994	0.8194	1.342	MUSH	1
404	V3-c1-acc-co-n3a.tif	2.507	1.0728	2.337	FILO	1
406	V3-c1-acc-co-n3a.tif	1.2	0.7329	1.637	MUSH	1
408	V3-c1-acc-co-n3a.tif	1.513	0.8042	1.881	MUSH	1
410	V3-c1-acc-co-n3a.tif	1.6763	1.5779	1.062	MUSH	1
						**12**
412	V3-c1-acc-co-n4.tif	0.8743	0.8357	1.046	MUSH	1
414	V3-c1-acc-co-n4.tif	1.1391	0.8684	1.312	MUSH	1
416	V3-c1-acc-co-n4.tif	1.9609	0.8576	2.286	MUSH	1
418	V3-c1-acc-co-n4.tif	1.3914	0.9589	1.451	MUSH	1
420	V3-c1-acc-co-n4.tif	0.9979	0.762	1.310	MUSH	1
422	V3-c1-acc-co-n4.tif	1.1245	0.8194	1.372	MUSH	1
424	V3-c1-acc-co-n4.tif	0.8295	0.6508	1.275	MUSH	1
426	V3-c1-acc-co-n4.tif	1.9719	0.9371	2.104	MUSH	1
428	V3-c1-acc-co-n4.tif	1.6676	1.0641	1.567	MUSH	1
						**9**
430	V3-c1-acc-co-n7.tif	1.2629	0.8344	1.514	MUSH	1
432	V3-c1-acc-co-n7.tif	1.4142	1.1615	1.218	MUSH	1
434	V3-c1-acc-co-n7.tif	0.9556	1.0365	0.922	MUSH	1
436	V3-c1-acc-co-n7.tif	1.3252	0.7781	1.703	MUSH	1

438	V3-c1-acc-co-n7.tif	1.5381	1.1827	1.300	MUSH	1
440	V3-c1-acc-co-n7.tif	1.1589	1.2481	0.929	MUSH	1
442	V3-c1-acc-co-n7.tif	2.8995	0.8194	3.539	FILO	1
						**7**
444	V3-c1-acc-co-n7a.tif	2.8669	0.8182	3.504	FILO	1
446	V3-c1-acc-co-n7a.tif	0.8381	0.575	1.458	THIN	1
448	V3-c1-acc-co-n7a.tif	1.4039	0.8381	1.675	MUSH	1
450	V3-c1-acc-co-n7a.tif	1.6916	0.6818	2.481	MUSH	1
452	V3-c1-acc-co-n7a.tif	0.9589	0.9556	1.003	MUSH	1
454	V3-c1-acc-co-n7a.tif	0.9642	0.5473	1.762	THIN	1
456	V3-c1-acc-co-n7a.tif	1.6389	0.6115	2.680	MUSH	1
458	V3-c1-acc-co-n7a.tif	2.1993	0.8131	2.705	FILO	1
460	V3-c1-acc-co-n7a.tif	2.0419	0.6938	2.943	FILO	1
462	V3-c1-acc-co-n7a.tif	1.9183	0.6923	2.771	MUSH	1
464	V3-c1-acc-co-n7a.tif	2.0939	0.8194	2.555	FILO	1
466	V3-c1-acc-co-n7a.tif	0.8381	0.6115	1.371	MUSH	1
						**12**
247	V3-c1-acc-co-n5.tif	0.1347	0.0299	4.505	THIN	1
249	V3-c1-acc-co-n5.tif	0.0868	0.1046	0.830	STUB	1
251	V3-c1-acc-co-n5.tif	0.1115	0.0541	2.061	THIN	1
253	V3-c1-acc-co-n5.tif	0.0497	0.0328	1.515	THIN	1
255	V3-c1-acc-co-n5.tif	0.1477	0.0456	3.239	THIN	1
257	V3-c1-acc-co-n5.tif	0.0834	0.0521	1.601	THIN	1
259	V3-c1-acc-co-n5.tif	0.1935	0.0409	4.731	THIN	1
261	V3-c1-acc-co-n5.tif	0.18	0.0734	2.452	THIN	1
263	V3-c1-acc-co-n5.tif	0.0899	0.0629	1.429	THIN	1
265	V3-c1-acc-co-n5.tif	0.1174	0.0677	1.734	THIN	1
267	V3-c1-acc-co-n5.tif	0.1742	0.0429	4.061	THIN	1
269	V3-c1-acc-co-n5.tif	0.1022	0.0742	1.377	THIN	1
271	V3-c1-acc-co-n5.tif	0.0731	0.0534	1.369	THIN	1
273	V3-c1-acc-co-n5.tif	0.0745	0.0501	1.487	THIN	1
						**14**
1	V4-c1-acc-co-n2.tif	2.1608	1.1331	1.907	FILO	1
3	V4-c1-acc-co-n2.tif	2.3391	1.2167	1.922	FILO	1
5	V4-c1-acc-co-n2.tif	2.3111	1.1643	1.985	FILO	1
7	V4-c1-acc-co-n2.tif	1.4689	1.2385	1.186	MUSH	1
9	V4-c1-acc-co-n2.tif	2.4146	1.1933	2.023	FILO	1
11	V4-c1-acc-co-n2.tif	2.0732	0.9255	2.240	FILO	1
13	V4-c1-acc-co-n2.tif	2.034	1.0223	1.990	FILO	1
15	V4-c1-acc-co-n2.tif	1.8006	1.0798	1.668	MUSH	1
17	V4-c1-acc-co-n2.tif	1.8372	1.6584	1.108	MUSH	1
19	V4-c1-acc-co-n2.tif	0.7302	0.8912	0.819	MUSH	1
21	V4-c1-acc-co-n2.tif	0.8636	0.7937	1.088	MUSH	1
23	V4-c1-acc-co-n2.tif	1.2385	1.0553	1.174	MUSH	1
25	V4-c1-acc-co-n2.tif	0.8624	0.9342	0.923	MUSH	1
						**13**
27	V4-c1-acc-co-n6.tif	1.3798	1.2017	1.148	MUSH	1
29	V4-c1-acc-co-n6.tif	1.1746	0.7724	1.521	MUSH	1

31	V4-c1-acc-co-n6.tif	1.6218	1.0836	1.497	MUSH	1
33	V4-c1-acc-co-n6.tif	0.9212	0.9846	0.936	MUSH	1
35	V4-c1-acc-co-n6.tif	1.3427	0.7943	1.690	MUSH	1
37	V4-c1-acc-co-n6.tif	1.0553	0.8665	1.218	MUSH	1
39	V4-c1-acc-co-n6.tif	1.3867	0.9337	1.485	MUSH	1
41	V4-c1-acc-co-n6.tif	1.0223	0.7134	1.433	MUSH	1
43	V4-c1-acc-co-n6.tif	0.7099	0.7384	0.961	MUSH	1
45	V4-c1-acc-co-n6.tif	1.5412	0.6164	2.500	MUSH	1
47	V4-c1-acc-co-n6.tif	1.208	0.7329	1.648	MUSH	1
49	V4-c1-acc-co-n6.tif	0.9337	0.9096	1.026	MUSH	1
						**12**
51	V4-c1-acc-co-n7.tif	2.0955	0.7308	2.867	FILO	1
53	V4-c1-acc-co-n7.tif	1.8546	0.955	1.942	MUSH	1
55	V4-c1-acc-co-n7.tif	1.2381	0.938	1.320	MUSH	1
57	V4-c1-acc-co-n7.tif	1.175	0.6749	1.741	MUSH	1
59	V4-c1-acc-co-n7.tif	1.3914	0.7546	1.844	MUSH	1
61	V4-c1-acc-co-n7.tif	2.1785	1.2213	1.784	FILO	1
63	V4-c1-acc-co-n7.tif	3.9397	0.9255	4.257	FILO	1
65	V4-c1-acc-co-n7.tif	1.3397	0.7546	1.775	MUSH	1
67	V4-c1-acc-co-n7.tif	0.9655	0.5965	1.619	THIN	1
69	V4-c1-acc-co-n7.tif	2.4281	0.7438	3.264	FILO	1
						**10**
72	V4-c2-acc-co-n1.tif	1.9759	1.5055	1.312	MUSH	1
74	V4-c2-acc-co-n1.tif	1.8773	0.9862	1.904	MUSH	1
76	V4-c2-acc-co-n1.tif	0.8012	0.8236	0.973	MUSH	1
78	V4-c2-acc-co-n1.tif	1.198	0.8278	1.447	MUSH	1
80	V4-c2-acc-co-n1.tif	1.4569	1.2797	1.138	MUSH	1
82	V4-c2-acc-co-n1.tif	1.126	1.0084	1.117	MUSH	1
84	V4-c2-acc-co-n1.tif	0.8945	0.914	0.979	MUSH	1
86	V4-c2-acc-co-n1.tif	1.5399	0.9228	1.669	MUSH	1
						**8**
88	V4-c2-acc-co-n5.tif	1.9384	1.2572	1.542	MUSH	1
90	V4-c2-acc-co-n5.tif	1.6829	0.8896	1.892	MUSH	1
92	V4-c2-acc-co-n5.tif	4.5125	0.8585	5.256	FILO	1
94	V4-c2-acc-co-n5.tif	2.5754	1.1527	2.234	FILO	1
96	V4-c2-acc-co-n5.tif	1.3418	1.0859	1.236	MUSH	1
98	V4-c2-acc-co-n5.tif	4.2775	0.8947	4.781	FILO	1
100	V4-c2-acc-co-n5.tif	1.3102	1.3006	1.007	MUSH	1
102	V4-c2-acc-co-n5.tif	2.0887	1.2499	1.671	FILO	1
						**8**
247	V5-c1-acc-co-n1a.tif	1.225	0.7937	1.543	MUSH	1
249	V5-c1-acc-co-n1a.tif	2.5208	0.8665	2.909	FILO	1
251	V5-c1-acc-co-n1a.tif	1.9767	0.6749	2.929	MUSH	1
253	V5-c1-acc-co-n1a.tif	1.6215	1.0596	1.530	MUSH	1
255	V5-c1-acc-co-n1a.tif	1.4799	1.3706	1.080	MUSH	1
257	V5-c1-acc-co-n1a.tif	1.9927	1.0572	1.885	MUSH	1
259	V5-c1-acc-co-n1a.tif	1.2217	0.8671	1.409	MUSH	1
261	V5-c1-acc-co-n1a.tif	2.8964	1.175	2.465	FILO	1

263	V5-c1-acc-co-n1a.tif	1.0812	0.8542	1.266	MUSH	1
265	V5-c1-acc-co-n1a.tif	1.6465	1.0238	1.608	MUSH	1
267	V5-c1-acc-co-n1a.tif	2.0327	0.9846	2.064	FILO	1
269	V5-c1-acc-co-n1a.tif	1.2511	0.8826	1.418	MUSH	1
271	V5-c1-acc-co-n1a.tif	1.4907	1.1929	1.250	MUSH	1
273	V5-c1-acc-co-n1a.tif	1.9074	0.6984	2.731	MUSH	1
						**14**
275	V5-c1-acc-co-n5.tif	0.7937	0.9024	0.880	MUSH	1
277	V5-c1-acc-co-n5.tif	1.6218	0.5089	3.187	LONG THIN	1
279	V5-c1-acc-co-n5.tif	1.4482	0.7049	2.054	MUSH	1
281	V5-c1-acc-co-n5.tif	0.8895	0.5854	1.519	THIN	1
283	V5-c1-acc-co-n5.tif	1.7925	0.9805	1.828	MUSH	1
285	V5-c1-acc-co-n5.tif	0.6023	0.6933	0.869	MUSH	1
287	V5-c1-acc-co-n5.tif	1.8192	0.9228	1.971	MUSH	1
289	V5-c1-acc-co-n5.tif	1.1508	0.6428	1.790	MUSH	1
291	V5-c1-acc-co-n5.tif	2.0974	1.0129	2.071	FILO	1
293	V5-c1-acc-co-n5.tif	1.4158	0.8242	1.718	MUSH	1
295	V5-c1-acc-co-n5.tif	1.7272	0.6984	2.473	MUSH	1
297	V5-c1-acc-co-n5.tif	0.7013	0.5406	1.297	THIN	1
299	V5-c1-acc-co-n5.tif	0.7841	0.8012	0.979	MUSH	1
301	V5-c1-acc-co-n5.tif	2.0357	0.604	3.370	FILO	1
						**14**
303	V5-c1-acc-co-n2.tif	0.5553	0.5553	1.000	STUB	1
305	V5-c1-acc-co-n2.tif	1.2923	1.138	1.136	MUSH	1
307	V5-c1-acc-co-n2.tif	1.9736	0.9887	1.996	MUSH	1
309	V5-c1-acc-co-n2.tif	1.5867	0.7239	2.192	MUSH	1
311	V5-c1-acc-co-n2.tif	1.4172	1.0164	1.394	MUSH	1
313	V5-c1-acc-co-n2.tif	1.624	0.8665	1.874	MUSH	1
315	V5-c1-acc-co-n2.tif	1.5122	1.126	1.343	MUSH	1
317	V5-c1-acc-co-n2.tif	1.2579	0.7841	1.604	MUSH	1
319	V5-c1-acc-co-n2.tif	2.0887	0.899	2.323	FILO	1
321	V5-c1-acc-co-n2.tif	1.4863	0.8895	1.671	MUSH	1
						**10**
323	V5-c1-acc-co-n3.tif	0.8717	0.6349	1.373	MUSH	1
325	V5-c1-acc-co-n3.tif	1.9018	0.8435	2.255	MUSH	1
327	V5-c1-acc-co-n3.tif	0.9676	0.5019	1.928	THIN	1
329	V5-c1-acc-co-n3.tif	0.5425	0.7411	0.732	MUSH	1
331	V5-c1-acc-co-n3.tif	1.2418	0.6734	1.844	MUSH	1
333	V5-c1-acc-co-n3.tif	0.7492	0.4846	1.546	THIN	1
335	V5-c1-acc-co-n3.tif	0.9545	0.5723	1.668	THIN	1
337	V5-c1-acc-co-n3.tif	2.8104	0.7956	3.532	FILO	1
339	V5-c1-acc-co-n3.tif	2.0446	0.8895	2.299	FILO	1
341	V5-c1-acc-co-n3.tif	0.8081	0.6749	1.197	MUSH	1
343	V5-c1-acc-co-n3.tif	1.8404	0.8542	2.155	MUSH	1
						**11**
345	V5-c1-acc-co-n4.tif	1.0224	1.2169	0.840	MUSH	1
347	V5-c1-acc-co-n4.tif	3.0791	0.9712	3.170	FILO	1
349	V5-c1-acc-co-n4.tif	2.6104	1.0994	2.374	FILO	1

351	V5-c1-acc-co-n4.tif	2.0028	0.9817	2.040	FILO	1
353	V5-c1-acc-co-n4.tif	0.9771	0.8425	1.160	MUSH	1
355	V5-c1-acc-co-n4.tif	1.9524	1.2866	1.517	MUSH	1
357	V5-c1-acc-co-n4.tif	1.2463	0.7079	1.761	MUSH	1
359	V5-c1-acc-co-n4.tif	1.8375	1.0011	1.835	MUSH	1
361	V5-c1-acc-co-n4.tif	1.1238	0.9098	1.235	MUSH	1
363	V5-c1-acc-co-n4.tif	1.9437	1.8547	1.048	MUSH	1
365	V5-c1-acc-co-n4.tif	1.2363	0.9583	1.290	MUSH	1
367	V5-c1-acc-co-n4.tif	1.381	1.1468	1.204	MUSH	1
369	V5-c1-acc-co-n4.tif	1.653	0.822	2.011	MUSH	1
371	V5-c1-acc-co-n4.tif	1.2997	0.6406	2.029	MUSH	1
373	V5-c1-acc-co-n4.tif	1.0224	0.8585	1.191	MUSH	1
						**15**
375	V5-c1-acc-co-n6.tif	1.6274	0.8112	2.006	MUSH	1
377	V5-c1-acc-co-n6.tif	1.3783	0.9035	1.526	MUSH	1
379	V5-c1-acc-co-n6.tif	2.1192	0.8979	2.360	FILO	1
381	V5-c1-acc-co-n6.tif	1.4482	1.1712	1.237	MUSH	1
383	V5-c1-acc-co-n6.tif	0.7841	0.5168	1.517	THIN	1
385	V5-c1-acc-co-n6.tif	1.1075	0.6537	1.694	MUSH	1
387	V5-c1-acc-co-n6.tif	1.1508	0.8381	1.373	MUSH	1
389	V5-c1-acc-co-n6.tif	2.002	0.9035	2.216	FILO	1
391	V5-c1-acc-co-n6.tif	1.5816	0.7302	2.166	MUSH	1
393	V5-c1-acc-co-n6.tif	1.4799	0.8577	1.725	MUSH	1
						**10**
530	V7-c1-acc-co-n1.tif	2.6608	0.98	2.715	FILO	1
532	V7-c1-acc-co-n1.tif	3.4696	1.1107	3.124	FILO	1
534	V7-c1-acc-co-n1.tif	1.1707	0.8333	1.405	MUSH	1
536	V7-c1-acc-co-n1.tif	1.3246	0.599	2.211	LONG THIN	1
538	V7-c1-acc-co-n1.tif	0.6868	0.6537	1.051	MUSH	1
540	V7-c1-acc-co-n1.tif	1.1002	0.8094	1.359	MUSH	1
542	V7-c1-acc-co-n1.tif	1.7155	0.5749	2.984	LONG THIN	1
544	V7-c1-acc-co-n1.tif	2.3346	0.8278	2.820	FILO	1
546	V7-c1-acc-co-n1.tif	1.2969	0.5723	2.266	LONG THIN	1
548	V7-c1-acc-co-n1.tif	1.9541	0.7438	2.627	MUSH	1
550	V7-c1-acc-co-n1.tif	1.4822	0.8548	1.734	MUSH	1
						**11**
552	V7-c1-acc-co-n3.tif	1.4537	0.9452	1.538	MUSH	1
554	V7-c1-acc-co-n3.tif	1.4514	1.1761	1.234	MUSH	1
556	V7-c1-acc-co-n3.tif	1.4945	1.1761	1.271	MUSH	1
558	V7-c1-acc-co-n3.tif	1.2381	0.7678	1.613	MUSH	1
560	V7-c1-acc-co-n3.tif	1.8547	0.8316	2.230	MUSH	1
562	V7-c1-acc-co-n3.tif	1.9846	0.7678	2.585	MUSH	1
564	V7-c1-acc-co-n3.tif	0.8755	1.0487	0.835	MUSH	1
566	V7-c1-acc-co-n3.tif	2.2902	0.9536	2.402	FILO	1
568	V7-c1-acc-co-n3.tif	1.5829	1.0102	1.567	MUSH	1
						**9**
570	V7-c1-acc-co-n4.tif	2.3294	0.9471	2.460	FILO	1
572	V7-c1-acc-co-n4.tif	0.8548	0.8453	1.011	MUSH	1

574	V7-c1-acc-co-n4.tif	2.2762	0.7253	3.138	FILO	1
576	V7-c1-acc-co-n4.tif	1.2856	0.6428	2.000	MUSH	1
578	V7-c1-acc-co-n4.tif	1.5321	0.8012	1.912	MUSH	1
580	V7-c1-acc-co-n4.tif	1.2217	0.9963	1.226	MUSH	1
582	V7-c1-acc-co-n4.tif	1.8181	0.8577	2.120	MUSH	1
584	V7-c1-acc-co-n4.tif	1.9889	0.5236	3.799	LONG THIN	1
586	V7-c1-acc-co-n4.tif	1.5357	0.5434	2.826	LONG THIN	1
						**9**
588	V7-c1-acc-co-n7.tif	1.3469	0.9098	1.480	MUSH	1
590	V7-c1-acc-co-n7.tif	0.9524	0.8624	1.104	MUSH	1
592	V7-c1-acc-co-n7.tif	0.9712	0.7143	1.360	MUSH	1
594	V7-c1-acc-co-n7.tif	1.1359	1.0191	1.115	MUSH	1
596	V7-c1-acc-co-n7.tif	1.6176	0.9524	1.698	MUSH	1
598	V7-c1-acc-co-n7.tif	0.9642	1.5193	0.635	MUSH	1
600	V7-c1-acc-co-n7.tif	1.4483	1.0224	1.417	MUSH	1
						**7**
602	V7-c1-acc-co-n8.tif	1.3744	1.4785	0.930	MUSH	1
604	V7-c1-acc-co-n8.tif	1.7619	1.3076	1.347	MUSH	1
606	V7-c1-acc-co-n8.tif	1.6007	0.6389	2.505	MUSH	1
608	V7-c1-acc-co-n8.tif	1.7202	0.6209	2.770	MUSH	1
610	V7-c1-acc-co-n8.tif	1.2019	0.6061	1.983	MUSH	1
612	V7-c1-acc-co-n8.tif	0.916	0.822	1.114	MUSH	1
614	V7-c1-acc-co-n8.tif	1.4915	1.0994	1.357	MUSH	1
616	V7-c1-acc-co-n8.tif	0.8316	0.7882	1.055	MUSH	1
618	V7-c1-acc-co-n8.tif	0.963	0.7619	1.264	MUSH	1
						**9**
620	V7-c3-acc-co-n1.tif	1.9937	1.4144	1.410	MUSH	1
622	V7-c3-acc-co-n1.tif	2.0163	1.0553	1.911	FILO	1
624	V7-c3-acc-co-n1.tif	1.2511	1.0515	1.190	MUSH	1
626	V7-c3-acc-co-n1.tif	1.3983	0.9294	1.505	MUSH	1
628	V7-c3-acc-co-n1.tif	1.5429	0.955	1.616	MUSH	1
630	V7-c3-acc-co-n1.tif	2.3774	0.9805	2.425	FILO	1
632	V7-c3-acc-co-n1.tif	0.7197	0.8758	0.822	MUSH	1
						7

**Animal**	**CORE**						
**V2**	FILO	MUSH	LONG THIN	THIN	STUBBY	BRANCHED	TOTAL
N1	4	9	0	0	0	0	13
N1A	0	6	1	0	0	0	7
N3	4	7	1	0	0	2	14
N4	2	4	0	0	0	0	6
N5	4	2	0	0	0	0	6
N6	3	9	0	0	0	0	12
**V2 average**	**2.83**	**6.17**	**0.33**	**0.00**	**0.00**	**0.33**	**9.67**

**V3**	FILO	MUSH	LONG THIN	THIN	STUBBY	BRANCHED	TOTAL

N3A	1	11	0	0	0	0	12
N4	0	9	0	0	0	0	9
N7	1	6	0	0	1	0	8
N7A	4	6	0	2	0	0	12
N5	0	0	0	13	1	0	14
**V3 average**	**1.2**	**6.4**	**0**	**3**	**0.4**	**0**	**11**

**V4**	FILO	MUSH	LONG THIN	THIN	STUBBY	BRANCHED	TOTAL
N2	6	7	0	0	0	0	13
**N6**	0	12	0	0	0	0	12
N7	4	5	0	1	0	1	11
**N1**	0	8	0	0	0	1	9
N5	4	4	0	0	0	2	10
**V4 average**	**2.8**	**7.2**	**0**	**0.2**	**0**	**0.8**	**11**

**V5**	FILO	MUSH	LONG THIN	THIN	STUBBY	BRANCHED	TOTAL
N1A	3	11	1	0	0	0	15
**N5**	2	9	0	2	0	2	15
N2	1	8	0	0	1	2	12
**N3**	2	6	0	3	0	1	12
N4	3	12	0	0	0	0	15
**N6**	2	7	0	1	0	1	11
**V5 average**	**2.17**	**8.83**	**0.17**	**1.00**	**0.17**	**1.00**	**13.33**

**V7**	FILO	MUSH	LONG THIN	THIN	STUBBY	BRANCHED	TOTAL
**N1**	1	8	0	0	0	0	9
**N4**	2	5	2	0	0	0	9
**N3**	1	8	0	0	0	1	10
**N7**	0	7	0	0	0	0	7
**N8**	0	9	0	0	0	0	9
**N1C3**	2	5	0	0	0	0	7
**V7 average**	**1.00**	**7.00**	**0.33**	**0.00**	**0.00**	**0.17**	**8.50**

CORE

**ANIMAL**	**FILO**	**MUSH**	**LONG THIN**	**THIN**	**STUBBY**	**BRANCHED**	**TOTAL**
**V2**	**2.83**	**6.17**	**0.33**	**0.00**	**0.00**	**0.33**	**9.67**
**V3**	**1.2**	**6.4**	**0**	**3**	**0.4**	**0**	**11**
**V4**	**2.8**	**7.2**	**0**	**0.2**	**0**	**0.8**	**11**
**V5**	**2.17**	**8.83**	**0.17**	**1.00**	**0.17**	**1.00**	**13.33**
**V7**	**1.00**	**7.00**	**0.33**	**0.00**	**0.00**	**0.17**	**8.50**

Percentage
CORE

**ANIMAL**	**FILO**	**MUSH**	**LONG THIN**	**THIN**	**STUBBY**	**BRANCHED**	**TOTAL**

**V2**	######	63.793	3.448	0.000	0.000	3.448	100
**V3**	######	58.182	0.000	27.273	3.636	0.000	100
**V4**	######	65.455	0.000	1.818	0.000	7.273	100
**V5**	######	66.250	1.250	7.500	1.250	7.500	100
**V7**	######	82.353	3.922	0.000	0.000	1.961	100

SCM
Females 48h
NAcc
SHELL
Spines density

	Animal	Length	Width	Indice L/W	Subtype	Number
347	V2-c1-acc-sh-n1.tif	0.9296	0.763	1.218	MUSH	1
349	V2-c1-acc-sh-n1.tif	1.38	0.6837	2.018	MUSH	1
351	V2-c1-acc-sh-n1.tif	1.4662	0.8912	1.645	MUSH	1
353	V2-c1-acc-sh-n1.tif	1.7102	1.1803	1.449	MUSH	1
355	V2-c1-acc-sh-n1.tif	1.2305	0.7094	1.735	MUSH	1
357	V2-c1-acc-sh-n1.tif	0.9296	1.5323	0.607	MUSH	1
359	V2-c1-acc-sh-n1.tif	0.8741	1.7734	0.493	MUSH	1
361	V2-c1-acc-sh-n1.tif	1.103	1.7238	0.640	MUSH	1
363	V2-c1-acc-sh-n1.tif	0.7273	1.8985	0.383	MUSH	1
365	V2-c1-acc-sh-n1.tif	1.0602	1.6559	0.640	MUSH	1
367	V2-c1-acc-sh-n1.tif	1.0761	1.6492	0.652	MUSH	1
369	V2-c1-acc-sh-n1.tif	0.9296	2.4367	0.381	MUSH	1
	V2-c1-acc-sh-n1.tif				BRANCH	1
371	V2-c1-acc-sh-n1.tif	0.9216	0.8788	1.049	MUSH	1
						**14**
165	V2-c1-acc-sh-n2.tif	1.1571	1.3514	0.856	MUSH	1
167	V2-c1-acc-sh-n2.tif	2.4159	1.4157	1.707	FILO	1
169	V2-c1-acc-sh-n2.tif	2.8622	0.7727	3.704	FILO	1
171	V2-c1-acc-sh-n2.tif	3.0458	0.8054	3.782	FILO	1
173	V2-c1-acc-sh-n2.tif	2.3222	0.778	2.985	FILO	1
175	V2-c1-acc-sh-n2.tif	0.7619	0.7115	1.071	MUSH	1
177	V2-c1-acc-sh-n2.tif	1.3644	0.8455	1.614	MUSH	1
179	V2-c1-acc-sh-n2.tif	1.8741	0.6711	2.793	MUSH	1
181	V2-c1-acc-sh-n2.tif	2.4638	1.356	1.817	FILO	1
						**9**
183	V2-c1-acc-sh-n3.tif	0.7287	0.5926	1.230	STUB	1
185	V2-c1-acc-sh-n3.tif	0.8381	0.5082	1.649	STUB	1
187	V2-c1-acc-sh-n3.tif	2.2827	1.6521	1.382	FILO	1
189	V2-c1-acc-sh-n3.tif	1.3824	0.9979	1.385	MUSH	1
191	V2-c1-acc-sh-n3.tif	1.3636	0.9969	1.368	MUSH	1
193	V2-c1-acc-sh-n3.tif	1.6122	1.3514	1.193	MUSH	1
195	V2-c1-acc-sh-n3.tif	0.9556	1.001	0.955	MUSH	1
197	V2-c1-acc-sh-n3.tif	1.7207	0.9759	1.763	MUSH	1
199	V2-c1-acc-sh-n3.tif	1.1589	0.6818	1.700	MUSH	1
201	V2-c1-acc-sh-n3.tif	1.7704	1.0164	1.742	MUSH	1
						**10**
203	V2-c2-acc-sh-n2.tif	3.6142	0.8321	4.343	FILO	1
205	V2-c2-acc-sh-n2.tif	1.2361	0.6837	1.808	MUSH	1
207	V2-c2-acc-sh-n2.tif	2.0319	0.7582	2.680	FILO	1
209	V2-c2-acc-sh-n2.tif	2.5887	0.5588	4.633	FILO	1
211	V2-c2-acc-sh-n2.tif	0.8912	0.7902	1.128	MUSH	1

213	V2-c2-acc-sh-n2.tif	1.8184	0.9968	1.824	MUSH	1
215	V2-c2-acc-sh-n2.tif	0.8187	0.5454	1.501	STUB	1
217	V2-c2-acc-sh-n2.tif	2.0599	0.7954	2.590	FILO	1
219	V2-c2-acc-sh-n2.tif	1.0739	0.4704	2.283	LONG THIN	1
221	V2-c2-acc-sh-n2.tif	0.5213	0.5292	0.985	STUB	1
223	V2-c2-acc-sh-n2.tif	1.1346	0.8159	1.391	MUSH	1
225	V2-c2-acc-sh-n2.tif	1.1113	0.7666	1.450	MUSH	1
	V2-c2-acc-sh-n2				BRANCH	1
227	V2-c2-acc-sh-n2.tif	0.8142	0.5463	1.490	STUB	1
						**14**
229	V2-c2-acc-sh-n3.tif	0.1018	0.0825	1.234	STUB	1
231	V2-c2-acc-sh-n3.tif	0.1304	0.0575	2.268	STUB	1
233	V2-c2-acc-sh-n3.tif	0.194	0.0792	2.449	STUB	1
235	V2-c2-acc-sh-n3.tif	0.1108	0.0593	1.868	STUB	1
237	V2-c2-acc-sh-n3.tif	0.0967	0.085	1.138	STUB	1
239	V2-c2-acc-sh-n3.tif	0.1341	0.0731	1.834	STUB	1
241	V2-c2-acc-sh-n3.tif	0.0713	0.082	0.870	STUB	1
243	V2-c2-acc-sh-n3.tif	0.1413	0.1612	0.877	STUB	1
245	V2-c2-acc-sh-n3.tif	0.2315	0.0877	2.640	STUB	1
						**9**
468	V3-c1-acc-sh-n2.tif	2.0954	1.0611	1.975	FILO	1
470	V3-c1-acc-sh-n2.tif	1.914	1.1686	1.638	MUSH	1
472	V3-c1-acc-sh-n2.tif	2.8895	0.9458	3.055	FILO	1
474	V3-c1-acc-sh-n2.tif	2.9148	0.893	3.264	FILO	1
476	V3-c1-acc-sh-n2.tif	1.754	0.6923	2.534	MUSH	1
478	V3-c1-acc-sh-n2.tif	2.0939	0.9148	2.289	FILO	1
480	V3-c1-acc-sh-n2.tif	1.4673	0.7951	1.845	MUSH	1
482	V3-c1-acc-sh-n2.tif	2.2768	0.9458	2.407	FILO	1
						**8**
484	V3-c1-acc-sh-n5.tif	2.1156	1.2305	1.719	FILO	1
486	V3-c1-acc-sh-n5.tif	1.2494	0.7861	1.589	MUSH	1
488	V3-c1-acc-sh-n5.tif	2.208	0.7228	3.055	FILO	1
490	V3-c1-acc-sh-n5.tif	2.3335	0.7931	2.942	FILO	1
492	V3-c1-acc-sh-n5.tif	0.9438	0.5395	1.749	STUB	1
494	V3-c1-acc-sh-n5.tif	0.8667	0.7068	1.226	MUSH	1
496	V3-c1-acc-sh-n5.tif	2.1871	0.5758	3.798	FILO	1
498	V3-c1-acc-sh-n5.tif	1.3667	0.7879	1.735	MUSH	1
						**8**
500	V3-c1-acc-sh-n7.tif	1.1118	0.7329	1.517	MUSH	1
502	V3-c1-acc-sh-n7.tif	1.1018	0.6646	1.658	MUSH	1
504	V3-c1-acc-sh-n7.tif	1.38	0.8159	1.691	MUSH	1
508	V3-c1-acc-sh-n7.tif	1.7481	0.8994	1.944	MUSH	1
510	V3-c1-acc-sh-n7.tif	1.0041	0.8271	1.214	MUSH	1
512	V3-c1-acc-sh-n7.tif	1.1249	0.763	1.474	MUSH	1
514	V3-c1-acc-sh-n7.tif	1.217	0.4412	2.758	LONG THIN	1
516	V3-c1-acc-sh-n7.tif	1.3528	0.5301	2.552	LONG THIN	1
						**8**
518	V3-c2-acc-sh-n5.tif	0.9486	0.7582	1.251	MUSH	1

520	V3-c2-acc-sh-n5.tif	1.4246	0.5969	2.387	LONG THIN	1
522	V3-c2-acc-sh-n5.tif	2.0608	0.8667	2.378	FILO	1
524	V3-c2-acc-sh-n5.tif	2.9488	0.7454	3.956	FILO	1
526	V3-c2-acc-sh-n5.tif	1.4799	0.5421	2.730	LONG THIN	1
528	V3-c2-acc-sh-n5.tif	1	1.2216	0.819	MUSH	1
530	V3-c2-acc-sh-n5.tif	2.1104	0.8576	2.461	FILO	1
532	V3-c2-acc-sh-n5.tif	1.2439	0.8006	1.554	MUSH	1
534	V3-c2-acc-sh-n5.tif	2.1619	0.7329	2.950	FILO	1
						**9**
1	V3-c2-acc-sh-n4.tif	0.7879	0.4848	1.625	STUB	1
3	V3-c2-acc-sh-n4.tif	0.8243	0.5954	1.384	STUB	1
5	V3-c2-acc-sh-n4.tif	0.5231	0.5231	1.000	STUB	1
7	V3-c2-acc-sh-n4.tif	1.248	0.553	2.257	LONG THIN	1
9	V3-c2-acc-sh-n4.tif	1.6983	0.691	2.458	MUSH	1
11	V3-c2-acc-sh-n4.tif	0.8918	0.5463	1.632	STUB	1
13	V3-c2-acc-sh-n4.tif	0.6976	0.4307	1.620	STUB	1
15	V3-c2-acc-sh-n4.tif	0.8485	0.6371	1.332	MUSH	1
17	V3-c2-acc-sh-n4.tif	0.8187	0.6128	1.336	MUSH	1
19	V3-c2-acc-sh-n4.tif	0.6976	0.5455	1.279	STUB	1
21	V3-c2-acc-sh-n4.tif	0.4886	0.5152	0.948	STUB	1
23	V3-c2-acc-sh-n4.tif	0.8793	0.7931	1.109	MUSH	1
25	V3-c2-acc-sh-n4.tif	1.0791	0.4066	2.654	LONG THIN	1
						**13**
104	V4-c2-acc-sh-n5.tif	1.1853	1.4565	0.814	MUSH	1
106	V4-c2-acc-sh-n5.tif	1.7633	1.1088	1.590	MUSH	1
108	V4-c2-acc-sh-n5.tif	1.2639	0.9841	1.284	MUSH	1
110	V4-c2-acc-sh-n5.tif	1.2675	0.8162	1.553	MUSH	1
112	V4-c2-acc-sh-n5.tif	0.8636	0.7329	1.178	MUSH	1
114	V4-c2-acc-sh-n5.tif	1.0947	0.599	1.828	LONG THIN	1
116	V4-c2-acc-sh-n5.tif	2.8018	0.6734	4.161	FILO	1
118	V4-c2-acc-sh-n5.tif	1.3499	0.7573	1.783	MUSH	1
						**8**
120	V4-c2-acc-sh-n6.tif	1.5569	0.6606	2.357	MUSH	1
122	V4-c2-acc-sh-n6.tif	1.1057	0.8381	1.319	MUSH	1
124	V4-c2-acc-sh-n6.tif	0.8912	0.7645	1.166	MUSH	1
126	V4-c2-acc-sh-n6.tif	3.1659	0.7239	4.373	FILO	1
128	V4-c2-acc-sh-n6.tif	0.8855	0.6428	1.378	MUSH	1
130	V4-c2-acc-sh-n6.tif	0.9862	0.7619	1.294	MUSH	1
132	V4-c2-acc-sh-n6.tif	0.8387	0.6261	1.340	MUSH	1
134	V4-c2-acc-sh-n6.tif	1.1183	0.8094	1.382	MUSH	1
136	V4-c2-acc-sh-n6.tif	1.0965	1.0794	1.016	MUSH	1
138	V4-c2-acc-sh-n6.tif	0.7626	0.7049	1.082	MUSH	1
140	V4-c2-acc-sh-n6.tif	1.386	0.6991	1.983	MUSH	1
142	V4-c2-acc-sh-n6.tif	1.3367	0.8979	1.489	MUSH	1
144	V4-c2-acc-sh-n6.tif	0.5625	0.5723	0.983	STUB	1
						**13**
146	V4-c2-acc-sh-n7a.tif	1.8674	0.8758	2.132	MUSH	1
148	V4-c2-acc-sh-n7a.tif	1.0794	0.8381	1.288	MUSH	1

150	V4-c2-acc-sh-n7a.tif	1.1251	1.2698	0.886	MUSH	1
152	V4-c2-acc-sh-n7a.tif	1.2063	0.9887	1.220	MUSH	1
154	V4-c2-acc-sh-n7a.tif	0.8843	0.7432	1.190	MUSH	1
156	V4-c2-acc-sh-n7a.tif	1.5291	0.7632	2.004	MUSH	1
158	V4-c2-acc-sh-n7a.tif	1.3588	1.1233	1.210	MUSH	1
160	V4-c2-acc-sh-n7a.tif	1.1446	0.6868	1.667	MUSH	1
162	V4-c2-acc-sh-n7a.tif	0.6389	0.8236	0.776	MUSH	1
164	V4-c2-acc-sh-n7a.tif	1.5762	0.7438	2.119	MUSH	1
						**10**
	V5-c1-acc-sh-n4.tif	1.0487	0.7529	1.393	MUSH	1
168	V5-c1-acc-sh-n4.tif	1.1359	0.8755	1.297	MUSH	1
170	V5-c1-acc-sh-n4.tif	1.9143	0.8571	2.233	MUSH	1
172	V5-c1-acc-sh-n4.tif	1.812	1	1.812	MUSH	1
174	V5-c1-acc-sh-n4.tif	1.5357	1.1107	1.383	MUSH	1
176	V5-c1-acc-sh-n4.tif	1.2572	0.7925	1.586	MUSH	1
178	V5-c1-acc-sh-n4.tif	1.341	0.6615	2.027	MUSH	1
180	V5-c1-acc-sh-n4.tif	2.2981	0.7619	3.016	FILO	1
182	V5-c1-acc-sh-n4.tif	1.2499	0.6477	1.930	MUSH	1
184	V5-c1-acc-sh-n4.tif	1.3342	0.7752	1.721	MUSH	1
186	V5-c1-acc-sh-n4.tif	1.7779	1.1448	1.553	MUSH	1
188	V5-c1-acc-sh-n4.tif	0.9943	1.2581	0.790	MUSH	1
190	V5-c1-acc-sh-n4.tif	1.0859	1.0796	1.006	MUSH	1
						**13**
192	V5-c1-acc-sh-n7.tif	2.4248	0.8278	2.929	FILO	1
194	V5-c1-acc-sh-n7.tif	1.9759	0.6514	3.033	MUSH	1
196	V5-c1-acc-sh-n7.tif	1.4634	1.0519	1.391	MUSH	1
198	V5-c1-acc-sh-n7.tif	0.7943	0.7943	1.000	MUSH	1
201	V5-c1-acc-sh-n7.tif	0.7632	0.7253	1.052	MUSH	1
202	V5-c1-acc-sh-n7.tif	0.7937	0.5679	1.398	STUB	1
204	V5-c1-acc-sh-n7.tif	1.4229	0.8131	1.750	MUSH	1
206	V5-c1-acc-sh-n7.tif	1.4634	0.6853	2.135	MUSH	1
208	V5-c1-acc-sh-n7.tif	1.1006	0.7937	1.387	MUSH	1
210	V5-c1-acc-sh-n7.tif	2.8089	0.7404	3.794	FILO	1
212	V5-c1-acc-sh-n7.tif	1.3925	0.7404	1.881	MUSH	1
214	V5-c1-acc-sh-n7.tif	1.3472	0.6106	2.206	MUSH	1
						**12**
216	V4-c2-acc-sh-n1.tif	2.4323	1.0487	2.319	FILO	1
219	V4-c2-acc-sh-n1.tif	1.0859	0.8193	1.325	MUSH	1
221	V4-c2-acc-sh-n1.tif	1.5547	1.2499	1.244	MUSH	1
223	V4-c2-acc-sh-n1.tif	1.8529	1.0102	1.834	MUSH	1
225	V4-c2-acc-sh-n1.tif	3.4539	0.9805	3.523	FILO	1
227	V4-c2-acc-sh-n1.tif	2.716	0.7982	3.403	FILO	1
229	V4-c2-acc-sh-n1.tif	0.8438	1.0701	0.789	MUSH	1
231	V4-c2-acc-sh-n1.tif	0.9536	1.0952	0.871	MUSH	1
233	V4-c2-acc-sh-n1.tif	1.4325	0.5151	2.781	LONG THIN	1
235	V4-c2-acc-sh-n1.tif	0.6933	0.9571	0.724	MUSH	1
237	V4-c2-acc-sh-n1.tif	2.9063	0.8947	3.248	FILO	1
239	V4-c2-acc-sh-n1.tif	1.7951	0.9817	1.829	MUSH	1

241	V4-c2-acc-sh-n1.tif	0.7079	0.9642	0.734	MUSH	1
243	V4-c2-acc-sh-n1.tif	1.009	0.9571	1.054	MUSH	1
245	V4-c2-acc-sh-n1.tif	1.4134	1.0701	1.321	MUSH	1
						**15**
395	V5-c1-acc-sh-n5.tif	1.0413	0.8381	1.242	MUSH	1
397	V5-c1-acc-sh-n5.tif	1.2954	0.8333	1.555	MUSH	1
399	V5-c1-acc-sh-n5.tif	1.0336	0.6734	1.535	MUSH	1
401	V5-c1-acc-sh-n5.tif	1.0648	0.7134	1.493	MUSH	1
403	V5-c1-acc-sh-n5.tif	0.7246	0.6098	1.188	MUSH	1
405	V5-c1-acc-sh-n5.tif	1.1165	0.6349	1.759	MUSH	1
407	V5-c1-acc-sh-n5.tif	0.8577	0.6962	1.232	MUSH	1
409	V5-c1-acc-sh-n5.tif	1.2954	0.955	1.356	MUSH	1
412	V5-c1-acc-sh-n5.tif	1.5291	0.7363	2.077	MUSH	1
414	V5-c1-acc-sh-n5.tif	1.0104	0.5905	1.711	LONG THIN	1
416	V5-c1-acc-sh-n5.tif	0.8671	0.8518	1.018	MUSH	1
418	V5-c1-acc-sh-n5.tif	0.9228	0.4318	2.137	STUB	1
						**12**
420	V5-c1-acc-sh-n7.tif	1.705	1.1448	1.489	MUSH	1
422	V5-c1-acc-sh-n7.tif	1.689	0.6263	2.697	MUSH	1
424	V5-c1-acc-sh-n7.tif	1.2866	0.7079	1.817	MUSH	1
426	V5-c1-acc-sh-n7.tif	2.5256	1.0011	2.523	FILO	1
428	V5-c1-acc-sh-n7.tif	2.5644	1.3908	1.844	FILO	1
430	V5-c1-acc-sh-n7.tif	0.8095	0.6667	1.214	MUSH	1
432	V5-c1-acc-sh-n7.tif	1.0487	0.8781	1.194	MUSH	1
434	V5-c1-acc-sh-n7.tif	3.1432	1.8596	1.690	FILO	1
						**8**
436	V5-c1-acc-sh-n4.tif	1.0796	0.781	1.382	MUSH	1
438	V5-c1-acc-sh-n4.tif	0.9817	1.1157	0.880	MUSH	1
440	V5-c1-acc-sh-n4.tif	1.7169	0.8095	2.121	MUSH	1
442	V5-c1-acc-sh-n4.tif	1.9143	0.8571	2.233	MUSH	1
444	V5-c1-acc-sh-n4.tif	1.4142	1.1157	1.268	MUSH	1
446	V5-c1-acc-sh-n4.tif	1.84	0.7529	2.444	MUSH	1
448	V5-c1-acc-sh-n4.tif	2.2059	0.8585	2.569	FILO	1
450	V5-c1-acc-sh-n4.tif	1.8133	0.7925	2.288	MUSH	1
452	V5-c1-acc-sh-n4.tif	1.2289	0.5734	2.143	LONG THIN	1
454	V5-c1-acc-sh-n4.tif	0.8742	0.6389	1.368	MUSH	1
456	V5-c1-acc-sh-n4.tif	1.0124	0.5574	1.816	LONG THIN	1
458	V5-c1-acc-sh-n4.tif	0.5062	0.6406	0.790	MUSH	1
						**12**
460	V5-c3-acc-sh-n2a.tif	1.488	1.5636	0.952	MUSH	1
462	V5-c3-acc-sh-n2a.tif	1.2068	0.9374	1.287	MUSH	1
464	V5-c3-acc-sh-n2a.tif	1.9582	1.3265	1.476	MUSH	1
467	V5-c3-acc-sh-n2a.tif	2.1421	1.0104	2.120	FILO	1
469	V5-c3-acc-sh-n2a.tif	3.7811	0.7841	4.822	FILO	1
471	V5-c3-acc-sh-n2a.tif	1.1273	0.5625	2.004	LONG THIN	1
473	V5-c3-acc-sh-n2a.tif	2.3172	0.4804	4.823	FILO	1
475	V5-c3-acc-sh-n2a.tif	1.2068	0.6962	1.733	MUSH	1
						**8**

503	V5-c1-acc-sh-n6.tif	1.5664	0.7454	2.101	MUSH	1
505	V5-c1-acc-sh-n6.tif	0.9817	0.8985	1.093	MUSH	1
507	V5-c1-acc-sh-n6.tif	1.5246	0.8109	1.880	MUSH	1
509	V5-c1-acc-sh-n6.tif	1.8596	0.6667	2.789	MUSH	1
511	V5-c1-acc-sh-n6.tif	1.1761	1.2141	0.969	MUSH	1
513	V5-c1-acc-sh-n6.tif	1.8784	0.8858	2.121	MUSH	1
515	V5-c1-acc-sh-n6.tif	2.3685	1.0045	2.358	FILO	1
517	V5-c1-acc-sh-n6.tif	1.268	0.7079	1.791	MUSH	1
519	V5-c1-acc-sh-n6.tif	0.7925	0.9583	0.827	MUSH	1
522	V5-c1-acc-sh-n6.tif	1.4317	0.8478	1.689	MUSH	1
524	V5-c1-acc-sh-n6.tif	1.6836	0.6406	2.628	MUSH	1
526	V5-c1-acc-sh-n6.tif	2.3338	0.963	2.423	FILO	1
528	V5-c1-acc-sh-n6.tif	0.5387	0.7079	0.761	MUSH	1
						**13**
477	V5-c2-acc-sh-n3.tif	1.5186	0.7031	2.160	MUSH	1
479	V5-c2-acc-sh-n3.tif	1.0573	0.6406	1.650	MUSH	1
481	V5-c2-acc-sh-n3.tif	0.9098	0.7408	1.228	MUSH	1
483	V5-c2-acc-sh-n3.tif	0.9035	0.8985	1.006	MUSH	1
485	V5-c2-acc-sh-n3.tif	1.0573	0.7925	1.334	MUSH	1
487	V5-c2-acc-sh-n3.tif	1.8596	0.7269	2.558	MUSH	1
489	V5-c2-acc-sh-n3.tif	1.5246	0.6098	2.500	MUSH	1
491	V5-c2-acc-sh-n3.tif	1.4884	0.9098	1.636	MUSH	1
493	V5-c2-acc-sh-n3.tif	1.3006	0.8896	1.462	MUSH	1
495	V5-c2-acc-sh-n3.tif	2.2638	0.5129	4.414	FILO	1
497	V5-c2-acc-sh-n3.tif	1.1359	0.5734	1.981	LONG THIN	1
499	V5-c2-acc-sh-n3.tif	0.7253	0.5967	1.216	STUB	1
501	V5-c2-acc-sh-n3.tif	0.8585	0.5553	1.546	STUB	1
						**13**
660	V7-c1-acc-sh-n8.tif	0.7492	0.7239	1.035	MUSH	1
662	V7-c1-acc-sh-n8.tif	1.0104	0.7492	1.349	MUSH	1
664	V7-c1-acc-sh-n8.tif	0.7438	0.6098	1.220	MUSH	1
666	V7-c1-acc-sh-n8.tif	1.4541	0.6606	2.201	MUSH	1
668	V7-c1-acc-sh-n8.tif	2.3035	0.7685	2.997	FILO	1
670	V7-c1-acc-sh-n8.tif	1.2262	0.4772	2.570	LONG THIN	1
672	V7-c1-acc-sh-n8.tif	0.7645	0.6962	1.098	MUSH	1
						**7**
674	V7-c1-acc-sh-n1a.tif	0.9524	1.0648	0.894	MUSH	1
676	V7-c1-acc-sh-n1a.tif	1.5829	1.1674	1.356	MUSH	1
678	V7-c1-acc-sh-n1a.tif	0.6061	0.6477	0.936	MUSH	1
680	V7-c1-acc-sh-n1a.tif	1.0102	0.8478	1.192	MUSH	1
682	V7-c1-acc-sh-n1a.tif	1.0775	0.7269	1.482	MUSH	1
684	V7-c1-acc-sh-n1a.tif	1.2499	3.6889	0.339	MUSH	1
686	V7-c1-acc-sh-n1a.tif	2.1592	0.9909	2.179	FILO	1
688	V7-c1-acc-sh-n1a.tif	2.7685	0.6615	4.185	FILO	1
691	V7-c1-acc-sh-n1a.tif	1.0476	0.6684	1.567	MUSH	1
						**9**
693	V7-c1-acc-sh-n4.tif	2.1484	0.8636	2.488	FILO	1
695	V7-c1-acc-sh-n4.tif	1.2213	0.8895	1.373	MUSH	1

697	V7-c1-acc-sh-n4.tif	1.7509	0.8381	2.089	MUSH	1
699	V7-c1-acc-sh-n4.tif	1.2155	0.8577	1.417	MUSH	1
701	V7-c1-acc-sh-n4.tif	1.0947	0.7404	1.479	MUSH	1
703	V7-c1-acc-sh-n4.tif	0.7841	0.5616	1.396	STUB	1
705	V7-c1-acc-sh-n4.tif	0.4719	0.4512	1.046	STUB	1
707	V7-c1-acc-sh-n4.tif	0.6545	0.4512	1.451	STUB	1
709	V7-c1-acc-sh-n4.tif	0.6697	0.8636	0.775	MUSH	1
711	V7-c1-acc-sh-n4.tif	1.2856	0.5397	2.382	LONG THIN	1
						**10**
713	V7-c1-acc-sh-n6.tif	1.5055	0.5406	2.785	LONG THIN	1
715	V7-c1-acc-sh-n6.tif	1.5042	0.8094	1.858	MUSH	1
717	V7-c1-acc-sh-n6.tif	1.1088	1.0924	1.015	MUSH	1
719	V7-c1-acc-sh-n6.tif	1.5122	0.6301	2.400	MUSH	1
721	V7-c1-acc-sh-n6.tif	1.3231	0.9963	1.328	MUSH	1
723	V7-c1-acc-sh-n6.tif	0.7632	0.6816	1.120	MUSH	1
725	V7-c1-acc-sh-n6.tif	0.6962	0.6697	1.040	MUSH	1
						7
729	V7-c1-acc-sh-n7.tif	1.1803	1.0519	1.122	MUSH	1
731	V7-c1-acc-sh-n7.tif	0.9669	1.3313	0.726	MUSH	1
733	V7-c1-acc-sh-n7.tif	2.3617	0.5758	4.102	FILO	1
735	V7-c1-acc-sh-n7.tif	0.6996	0.6506	1.075	MUSH	1
737	V7-c1-acc-sh-n7.tif	0.5563	0.8337	0.667	MUSH	1
739	V7-c1-acc-sh-n7.tif	1.0414	0.6327	1.646	MUSH	1
741	V7-c1-acc-sh-n7.tif	0.8381	0.7527	1.113	MUSH	1
743	V7-c1-acc-sh-n7.tif	1.4126	0.6136	2.302	MUSH	1
745	V7-c1-acc-sh-n7.tif	1.5949	0.6485	2.459	MUSH	1
						**9**
747	V7-c1-acc-sh-n8a.tif	1.5007	1.1589	1.295	MUSH	1
749	V7-c1-acc-sh-n8a.tif	1.065	1.0464	1.018	MUSH	1
751	V7-c1-acc-sh-n8a.tif	0.9664	0.6428	1.503	MUSH	1
753	V7-c1-acc-sh-n8a.tif	1.1931	0.7071	1.687	MUSH	1
755	V7-c1-acc-sh-n8a.tif	1.065	0.5821	1.830	LONG THIN	1
757	V7-c1-acc-sh-n8a.tif	1.2365	0.7565	1.635	MUSH	1
759	V7-c1-acc-sh-n8a.tif	1.7751	0.9371	1.894	MUSH	1
761	V7-c1-acc-sh-n8a.tif	0.6508	0.7399	0.880	MUSH	1
763	V7-c1-acc-sh-n8a.tif	0.5785	0.7071	0.818	MUSH	1
						**9**

Animal **SHELL**

**V2**	**FILO**	**MUSH**	**LONG THIN**	**THIN**	**STUBBY**	**BRANCHEI**	**TOTAL**
N1	0	13	0	0	0	1	14
N2	5	4	0	0	0	0	9
N3	1	7	0	0	2	0	10
N2 C2	4	5	1	0	3	1	14
N3 C3	0	0	0	0	9	0	9
**V2 average**	**2.000**	**5.8**	**0.2**	**0**	**2.800**	**0.4**	**11.2**

**V3**	**FILO**	**MUSH**	**LONG THIN**	**THIN**	**STUBBY**	**BRANCHEI**	**TOTAL**

N2	5	3	0	0	0	0	8
N5	4	3	0	**0**	1	0	8
N7	0	6	2	0	0	0	8
N5 C2	4	3	2	**0**	0	0	9
N6	0	4	2		7	0	13
**V3 average**	**2.6**	**3.8**	**1.2**	**0**	**1.6**	**0**	**9.2**

**V4**	**FILO**	**MUSH**	**LONG THIN**	**THIN**	**STUBBY**	**BRANCHEI**	**TOTAL**
N5	1	6	1	0	0	0	8
**N6**	1	11	0	0	1	**0**	13
N7A	0	10	0	0	0	2	12
**N1**	4	10	1	0	0	**0**	15
N							
**V4 average**	**1.5**	**9.25**	**0.5**	**0**	**0.25**	**0.5**	**12**

**V5**	**FILO**	**MUSH**	**LONG THIN**	**THIN**	**STUBBY**	**BRANCHEI**	**TOTAL**
N4	1	12	0	0	0	2	15
**N7**	2	9	0	0	1	0	12
N5	0	10	1	0	1	1	13
**N7 C2**	3	5	0	0	0	3	11
N4	1	9	2	0	0	0	12
**N2A**	3	4	1	0	0	0	8
N6	2	11	0	0	0	0	13
**N3**	1	9	1	0	2	1	14
V5 average	**1.625**	**8.625**	**0.625**	**0**	**0.5**	**0.875**	**12.25**

**V7**	**FILO**	**MUSH**	**LONG THIN**	**THIN**	**STUBBY**	**BRANCHEI**	**TOTAL**
N1A	2	7	0	0	0	0	9
N4	1	5	1	0	3	0	10
N6	0	6	1	0	0	2	9
N	1	8	0	0	0	2	11
N	0	8	1	0	0	1	10
**V7 average**	**0.80**	**6.80**	**0.60**	**0.00**	**0.60**	**1.00**	**9.80**

**SHELL ANIMAL**	**FILO**	**MUSH**	**LONG THIN**	**THIN**	**STUBBY**	**BRANCHEI**	**TOTAL**
**V2**	**2.000**	**5.8**	**0.2**	**0**	**2.800**	**0.4**	**11.2**
**V3**	**2.6**	**3.8**	**1.2**	**0**	**1.6**	**0**	**9.2**
**V4**	**1.5**	**9.25**	**0.5**	**0**	**0.25**	**0.5**	**12**
**V5**	**1.625**	**8.625**	**0.625**	**0**	**0.5**	**0.875**	**12.25**
**V7**	**0.80**	**6.80**	**0.60**	**0.00**	**0.60**	**1.00**	**9.80**

Percentage

**SHELL ANIMAL**	**FILO**	**MUSH**	**LONG THIN**	**THIN**	**STUBBY**	**BRANCHEI**	**TOTAL**

**V2**	17.86	51.79	1.79	0.00	25.00	3.57	100
**V3**	28.26	41.30	13.04	0.00	17.39	0.00	100
**V4**	12.50	77.08	4.17	0.00	2.08	4.17	100
**V5**	13.27	70.41	5.10	0.00	4.08	7.14	100
**V7**	8.16	69.39	6.12	0.00	6.12	10.20	100

SCM
Females 48h
NAcc
Core y Shell
Dendritic length

CORE

13	V2h-c1-co004	89.2908
14	V2h-c1-co004	55.2306
15	V2h-c1-co006	114.6793
	**V2**	**86.4002333**
17	V3h-c1-co001	147.0158
18	V3h-c1-co004	93.2559
19	V3h-c1-co004	92.332
20	V3h-c1-co005	148.8324
	**V3**	**120.359025**
30	V5h-c2-co003	135.9566
31	V5h-c2-co003	170.2129
32	V5h-c2-co.tif	126.7199
	**V5**	**144.296467**
33	V6h-c1-co3.tif	144.395
34	V6h-c1-co3.tif	129.1564
35	V6h-c1-co3.tif	104.1625
36	V6h-c1-co008	186.8752
37	V6h-c1-co008	157.5856
41	V6h-c2-co.tif	102.2997
42	V6h-c2-co.tif	127.5342
43	V6h-c2-co.tif	172.6503
44	V6h-c2-co.tif	93.4282
49	V6h-c2-co007	88.1952
50	V6h-c2-co007	140.1384
51	V6h-c2-co007	79.3989
52	V6h-c2-co007	169.1509
	**V6**	**130.382346**
63	V7h-c3-co002	119.9163
64	V7h-c3-co002	121.7509
65	V7h-c3-co008	103.1967
66	V7h-c2-co.tif	133.1828
67	V7h-c1-co002	95.3827
68	V7h-c1-co002	177.4666
	**V7**	**125.149333**

SHELL

21	V3h-c1-sh010	182.4287
22	V3h-c1-sh010	116.958
23	V3h-c1-sh009	114.2137
24	V3h-c1-sh009	104.0841
	**V3**	**129.421125**
25	V5h-c1-sh003	89.2958
26	V5h-c1-sh003	78.102
27	V5h-c2-sh001	180.2408

28	V5h-c2-sh001	202.8136
29	V5h-c2-sh001	112.6915
	**V5**	**132.62874**
38	V6h-c1-sh.tif	93.5826
39	V6h-c1-sh.tif	116.5811
40	V6h-c1-sh.tif	88.0368
45	V6h-c2-sh.tif	142.844
46	V6h-c2-sh.tif	153.4787
47	V6h-c2-sh.tif	109.1993
48	V6h-c2-sh.tif	78.4174
53	V6h-c2-sh2.tif	94.1147
54	V6h-c2-sh2.tif	80.5344
55	V6h-c2-sh2.tif	105.5944
56	V6h-c2-sh2.tif	130.1269
57	V6h-c3-sh004	125.0153
58	V6h-c3-sh004	210.569
59	V6h-c3-sh004	216.0405
60	V6h-c3-sh004	240.1715
	**V6**	**132.287107**
61	V7h-c2-sh2.tif	109.8434
62	V7h-c2-sh2.tif	63.777
69	V7h-c1-sh002	106.8426
	**V7**	**93.4876667**

13	V2h-c1-sh004	127.4
14	V2h-c1-sh004	124.21
15	V2h-c1-sh006	120.8593
	V2	**124.156433**

**ANIMAL**	**CORE**	**SHELL**
**V2**	**86.4**	**124.16**
**V3**	**120.36**	**129.42**
**V5**	**144.3**	**132.63**
**V6**	**130.38**	**132.29**
**V7**	**125.15**	**93.49**

Morfometric parameters
Control
Females
Core and Shel

CORE

ANIMAL	BRANCHED POINTS	SOMA DIAMETER
C1-5	1	18.85
C1-6	2	18.52
C1-9	0	18.2
C1-10	1	18
**C1**	**1**	**18.39**
C2-C1	1	14
C2-C1 N2	1	11.81
C2-C1-N3	1	15.2
C2-C2 N1	1	11.45
N2	0	10.24
N3	1	12.71
**C2**	**0.83**	**12.57**
C3-1-13 N1	0	14.18
N2	1	14.15
N3	1	18.45
N4	1	14.3
**C3**	**0.75**	**15.27**
C4-1 N1	1	16.45
N2	1	13.77
N3	1	13.8
N4	1	15.2
**C4**	**1.00**	**14.81**
C5-2 N1	1	15.2
N2	1	18.14
N3	0	15.04
**C5**	**0.67**	**16.13**
C6-1 N1	1	19.14
N2	1	15.18
N4	1	22.01
C6-2 N1	1	14.58
**C6**	**1.00**	**17.73**
C7 N1	1	17.73
N2	0	18.55
N3	0	15.3
N4	1	15.49
	1	18.45
**C7**	**0.60**	**17.10**

CORE ANIMAL	RANCHED POINT	SOMA DIAMETER	DENDRITIC LENGTH
**C1**	1	18.39	70.49
**C2**	0.83	12.57	73.38
**C3**	0.75	15.27	63.28
**C4**	1	14.81	121.82
**C5**	0.67	16.13	91.22
**C6**	1	17.73	99.14
**C7**	0.6	17.1	95.96

SHELL

ANIMAL	BRANCHED POINTS	SOMA DIAMETER
C1-100-N1	0	12.86
C1-100-N2	0	16.59
C1-2	1	16
C1-100-2	1	16.2
C1-100-6	1	12.7
**C1**	**0.6**	**14.87**
C2-C4 N1	0	14.79
N2	0	16.69
N3	1	18.2
C2-C6	1	18.6
**C2**	**0.5**	**17.07**
C3-1 N1	0	18.2
N2	0	13.2
N3	0	16.1
**C3**	**0.00**	**15.83**
C4-C3	1	12.08
N2	1	14.17
N3	1	16.65
N4	1	13.43
**C4**	**1.00**	**14.08**
C5-2 N1	1	19.5
N2	1	16.11
N3	1	12.9
N4	1	13.65
**C5**	**1**	**15.54**
C6N1	0	17.65
N2	0	12.01

N3	0	15.7
N4	0	12.94
**C6**	**0**	**14.58**
C7N2	0	23.19
C7N1	0	19.15
C7N3	0	18.26
**C7**	**0**	**20.2**

SHELL<br>ANIMAL	RANCHED POINT	SOMA DIAMETER	DENDRITIC LENGTH
**C1**	0.6	14.87	81.37
**C2**	0.5	17.07	78.94
**C3**	0	15.83	117.76
**C4**	1	14.08	115.56
**C5**	1	15.54	68.8
**C6**	0	14.58	132.1
**C7**	0	20.2	97.77

Morfometric parameters
Social Exposure
Females
Core and Shel

	CORE		
	ANIMAL	BRANCHED POINTS	SOMA DIAMETER
E1-c1		2	18.09
		1	14.45
		1	17.21
		1	13.48
**E1**		**1.25**	**15.81**
E2-c3		1	16.48
		1	13.99
		1	14.59
		0	13.67
**E2**		**0.75**	**14.6825**
E4-c2		1	12
		1	11.63
		1	16.72
		1	11.25
**E4**		**1**	**12.9**
E5-c1		2	18.83
		2	15.44
		2	16.2
		1	12.98
**E5**		**1.75**	**15.86**
E6-c1		3	16.32
		3	15.94
		2	18.1
		2	17.66
**E6**		**2.50**	**17.01**
E7-c1		2	15.62
		2	15.82
		1	16.66
		3	14.87
**E7**		**2.00**	**15.74**

CORE			
ANIMAL	BRANCHED POINTS	SOMA DIAMETER	DENDRITIC LENGTH
E1	0.25	15.81	99.06
E2	0.75	14.68	111.18
E4	1	12.9	98.62

E5	1.75	15.86	131.56
E6	2.5	17.01	188.32
E7	2	15.74	141.58

SHELL

ANIMAL	BRANCHED POINTS	SOMA DIAMETER
E1-c1	1	14.57
	0	19.68
	0	13.64
	1	9.87
**E1**	**0.5**	**14.44**
E2-c3	1	13.41
	1	12.59
	0	14.57
	1	12.49
**E2**	**0.75**	**13.265**
E4-c2	1	21.87
	0	19.31
	1	18.81
	1	15.21
**E4**	**0.75**	**18.8**
E5-c2	2	14.99
	2	16.21
	2	16.3
	3	15.61
E5	2.25	15.78
E6-c1	2	14.21
	1	17.85
	1	15.55
	2	14.13
	3	20.44
**E6**	**1.8**	**16.44**
E7-c1	2	20.93
	2	15.95
	3	13.37
	2	12.76
**E7**	**2.25**	**15.75**

SHELL

ANIMAL	BRANCHED POINTS	SOMA DIAMETER	DENDRITIC LENGTH
E1	0.5	14.44	116.24
E2	0.75	13.26	126.85

E4	0.75	18.8	168.36
E5	2.25	15.78	134.5
E6	1.8	16.44	188.35
E7	2.25	15.75	133.84

Morfometric parameters
Social cohabitation with mating
Females
Core and Shell

CORE

ANIMAL	BRANCHED POINTS	SOMA DIAMETER
V1-c1	0	10.55
	0	10.92
	1	16.56
	1	16.12
**V1**	**0.5**	**13.54**
V2-c2	1	16.3
	0	14.59
	0	16.25
	1	14.57
**V2**	**0.5**	**15.43**
V3-c1	2	16.33
	2	19.45
	1	16.11
	1	16.35
**V3**	**1.5**	**17.06**
V5-c1	1	17.21
	2	13.6
V5-c2	1	17.92
	1	19.63
**V5**	**1.25**	**17.09**
V6-c1	0	19.56
	1	15.02
V6-c2	1	14.37
	1	15.36
**V6**	**0.75**	**16.08**
V7-c1	0	16.03
	2	15.33
V7-c2	2	18.21
	1	18.73
**V7**	**1.25**	**17.08**

ANIMAL	BRANCHED POINTS	SOMA DIAMETER	DENDRITIC LENGTH
V1	0.5	13.54	86.4
V2	0.5	15.43	120.36
V3	1.5	17.06	144.3
V5	1.25	17.09	130.38

V6	0.75	16.08	125.15
V7	1.25	17.08	86.97

SHELL

ANIMAL	BRANCHED POINTS	SOMA DIAMETER
V1-c1	1	16.22
	1	13.61
	1	15.54
	0	15.43
**V1**	**0.75**	**15.2**
V2-c2	1	11.97
	1	11.24
	1	11.34
	1	15.47
**V2**	**1**	**12.51**
V3-c1	2	16.28
	0	11.58
**V3**	**1**	**13.93**
V5-c1	1	15.54
	2	17.45
	1	16.2
	1	16.48
**V5**	**1.25**	**16.42**
V6-c2	1	19.2
	1	17.39
	1	16.02
	1	18.38
**V6**	**1**	**17.75**
V7-c2	1	14.5
	1	17.56
	0	17
	1	14.62
**V7**	**0.75**	**15.92**

ANIMAL	BRANCHED POINTS	SOMA DIAMETER	DENDRITIC LENGTH
V1	0.75	15.2	124.16
V2	1	12.51	129.42
V3	1	13.93	132.63
V5	1.25	16.42	132.29
V6	1	17.75	93.49

V7	0.75	15.92	106.09
